# Regulatory Mechanisms of Free Umami Amino Acid Accumulation in Fresh Waxy Kernels: Insights from Transcriptome and Metabolomics Analyses

**DOI:** 10.3390/foods14213628

**Published:** 2025-10-24

**Authors:** Lin Zhao, Kaimei Huang, Letan Luo, Xiangqun Yu, Ning Shen, Yifan Wu, Jianguo Wu, Jiang Shi, Erkui Yue

**Affiliations:** 1 Institute of Crop and Ecology, Hangzhou Academy of Agricultural Sciences, Hangzhou 310024, China; zhaolin0227@163.com (L.Z.); llt15858164408@163.com (L.L.); yxqunhz@hotmail.com (X.Y.); wuyifan9877@163.com (Y.W.); 2 Hangzhou Agricultural Technology Extension Center, Hangzhou 310020, China; haungkm@163.com; 3 Hangzhou Rural Revitalization Service Center, Hangzhou 310016, China; s87071229@163.com; 4 College of Horticulture Science, Zhejiang A&F University, Hangzhou 311300, China; jianguowu@zafu.edu.cn

**Keywords:** fresh sweet waxy corn, umami amino acid, *O2*, *bZIPs*, small nucleolar RNA U3, ribosomal rRNA 28S

## Abstract

Free amino acids play a key role as flavor components and metabolic precursors in fresh corn, with umami taste largely attributing to their concentration, particularly the umami amino acid glutamate and aspartate. While several genes involved in the biosynthesis of these amino acids have been identified, their regulatory pathways remain poorly understood. Sweet waxy corn ‘Qianjiangnuo No.3’ (Q3) and waxy corn ‘Huayunhuanuo 402’ (H402), with contrasting umami taste, were used in this study. Transcriptomic, metabolomic, and targeted metabolite profiling were conducted at kernel-filling stage. Our analysis showed that Q3 ears possessed significantly higher levels of free amino acids than H402, and its umami amino acids were highly enriched. Diverse enrichment of amino acids was observed across the isolated kernels distribution in ears. The *O2* (*bZIP1*), *Naked endosperm1* (*NKD1*), and *bZIP* genes were found significantly downregulated in Q3 compared to H402. Conversely, genes involved in glutamate and aspartate biosynthesis showed higher expression in Q3 than H402 while translation-associated genes *snoRNA U3-2* and *rRNA28S* exhibited lower expression in Q3 than H402, correlating with a notable enrichment of free umami amino acids. Genes promoter analysis revealed an abundance of *bZIPs* binding motifs. These findings suggest that *bZIP* members may act as central regulators modulating free amino acid synthesis and accumulation in fresh kernels.

## 1. Introduction

Fresh corn is widely appreciated by consumers for its comprehensive advantages, including rich nutritional value, tender texture, and a diverse flavor profile. They are usually harvested at milky stage (19−26 days after pollination (DAP) and cooked by boiling or roasting [1]. It is generally classified into sweet corn and waxy corn. Especially, the sweet waxy corn, which belongs to waxy corn, possesses the excellent characteristics and nutritional value of both sweet corn and waxy corn. The same ear of sweet waxy corn has both sweet and waxy kernels, endowing with the sweetness and juiciness of sweet corn as well as the aroma and softness of waxy corn [2]. Due to its rich nutritional value and unique taste, sweet waxy corn has great potential for development. In addition, sweet waxy corn contains various free amino acids, such as umami amino acids, which serve as key flavor constituents and metabolic precursors, contributing to its multi-layered sensory experience [3]. The glutamate and aspartate are the primary contributors to umami taste. Glutamate forms monosodium glutamate in the presence of sodium ions, imparting a pronounced savory flavor, while aspartate enhances umami perception, particularly after thermal processing [4]. Consequently, free amino acids are considered essential flavored compounds that enhance both the sensory and nutritional qualities of fresh corn, and they are important consideration for human health and nutrition. Despite their importance, considerable heritable variation exists for amino acid concentrations in physiologically mature kernel of diverse maize panels [2,5,6,7,8], but a comparable level of genetic understanding is severely lacking for fresh-eating kernels (immature stage) of diverse fresh corn germplasm, particularly the genetic mechanisms underlying umami amino acid accumulation in sweet waxy corn kernels remain poorly understood.

In endosperm, a variety of primary metabolites such as sugars, RNAs, amino acids, and proteins are actively synthesized during kernel development [9,10,11]. Protein accumulation relies heavily on the availability of free amino acids, which serve as direct substrates for protein biosynthesis. The synthesis and accumulation of umami amino acids involve multiple genes. Glutamine synthetase (GS, Gln1 family) and glutamate synthase (GOGAT, Glu1 family) are critical for maintaining glutamate homeostasis [12,13], while aspartate aminotransferase (AspAT family) plays a central role in aspartate biosynthesis [14,15].

It is well known that the glutamate is the primary amino acid component in maize kernel protein. Its utilization efficiency is directly related to protein synthesis; that is, the translation efficiency of these amino acids also affects their accumulation, including glutamate and aspartate. Small nucleolar RNAs (snoRNAs), particularly U3 and U14, are essential for rRNA maturation, including 28S, 18S, and 5.8S subunits [16,17,18,19]. The rRNA 28S, in particular, functions as a catalytic core within the ribosome, playing crucial roles in peptide bond formation, translational fidelity, and overall protein synthesis [20,21]. As free amino acids are preferentially utilized for protein production during development, the proportion and absolute content of specific amino acids, such as glutamate typically decreases in mature kernels.

Transcription factors (TFs) are key regulators of plant growth, development, and metabolic processes, including amino acid metabolism. The basic leucine zipper (*bZIP*) family, for instance, participates in various eukaryotic metabolic pathways. In maize, the *Opaque2* (*O2*) gene, a central *bZIP* family member, regulates zein protein expression in the endosperm [22,23]. Mutations in *O2* reduce zein protein levels and result in elevated concentrations of free amino acids, including lysine, tryptophan, asparagine, aspartate and glutamate [24]. *O2* also interacts with other regulatory factors, such as *NKD1*, *NAC128/130*, *PBF1*, and *bZIP22*, forming a transcriptional network that governs protein synthesis [25,26,27,28,29,30,31]. Disruption of this network can reduce protein production efficiency and lead to increased free amino acid accumulation. However, whether *O2* and other *bZIPs* also regulate snoRNA U3, umami amino acid biosynthesis genes, and rRNA 28S expression in sweet waxy corn remain unclear. Furthermore, in view of most of the above genes, studies have focused on the quality traits in homozygous maize lines, where limitations are existed on sweet waxy corn correlative studies. Here, we distinguished sweet and waxy kernels in sweet waxy corn F2 ears, thus, utilizing kernel-type separated traits analysis in F2 ears with integrated omics is a better way to reveal the probable mechanism of *bZIPs* in sweet waxy corn in this study.

In this study, we used a sweet waxy corn variety Q3 (Zhejiang Approved Maize Variety:2017008), its amylose content is 1.9%, with high sensory and cooking qualities, and it emits a distinctive aroma and umami flavor after being steamed and cooked. In contrast, the control waxy variety H402 (National Approved Maize Variety: 20170043) exhibits comparatively lower quality in flavor, and its amylose content is 2%. To investigate the genetic basis of these differences, transcriptomic and metabolomic analyses were conducted on separated sweet and waxy kernels from both hybrids. This study aims to elucidate the genetic regulatory networks governing free amino acid metabolism in maize kernels, providing valuable insights for breeding new sweet waxy corn varieties with improved and distinctive flavor profiles.

## 2. Materials and Methods

The sweet waxy corn variety ‘Qianjiangnuo No.3’ (Q3), approved in Zhejiang Province, and the nationally approved waxy variety ‘Huayunhuanuo 402’ (H402) were selected as experimental materials in this study.

### 2.1. Sampling of Test Materials

The experiment was conducted at the Zhijiang Base of the Hangzhou Academy of Agricultural Sciences. To ensure uniformity in grain filling within each variety, plants exhibiting consistent and vigorous growth were selected for labeling and bagging. Artificial pollination was performed uniformly during the full flowering stage. Sweet waxy corn was sampled 22 days after pollination at milky stage. Six ears were taken from individual plants of each variety, and the kernels in the middle of each ear were collected. Then, they were immediately flash-frozen in liquid nitrogen and stored at −80 °C. Six ears were taken as one biological replication, and three biological duplicate samples were used in this experiment.

Additionally, six more ears were collected per variety. From each cob, sweet kernels (Q3-T and H402-T) and waxy kernels (Q3-N and H402-N) were separately identified from the central portion of the ear based on difference of light permeability, thoroughly mixed within each group, and stored at −80 °C for subsequent detection and sequencing.

### 2.2. Determination of Free Amino Acid Contents

Metabolite profiling in corn kernels was performed using liquid chromatography–tandem mass spectrometry (LC-MS/MS). A Shimadzu LC-30AD high-performance liquid chromatography (HPLC) system coupled with a SCIEX 6500 QTrap tandem mass spectrometer was employed for both qualitative and quantitative analysis. Unless otherwise specified, all reagents used were of analytical grade. Key reagents included hydrochloric acid, chromatographic-grade acetonitrile, ammonium formate, and a mixed standard solution containing 17 amino acids. Additionally, individual standards for asparagine, glutamine, and tryptophan were used for calibration and quantification

#### 2.2.1. Sample Extraction

Free amino acids of maize kernels were determined by Ruiyuan Biotechnology Co., Ltd. (Nanjing, China). A 0.5 g portion of sample powder was accurately weighed and transferred into a 10 mL centrifuge tube. Subsequently, 2 mL of 0.1 mol/L hydrochloric acid was added, and the mixture was thoroughly vortexed before being placed in an ice-water bath. The tube, along with its rack, was then placed in an ultrasonic cleaner for extraction at room temperature for 40 min, with manual shaking performed three times during the process. After extraction, the sample was centrifuged at 10,000 rpm for 10 min at 4 °C. The supernatant was collected, and the extraction was repeated with an additional 2 mL of 0.1 mol/L hydrochloric acid. Supernatants from both extractions were combined, filtered through a membrane filter, and subjected to LC-MS/MS analysis.

For standard curve preparation, a mixed standard solution containing 20 amino acids was prepared and serially diluted to five concentration levels: 0.015625 μmol/L, 0.03125 μmol/L, 0.0625 μmol/L, 0.125 μmol/L, and 0.25 μmol/L.

#### 2.2.2. Liquid Chromatography Conditions

Chromatographic separation was performed using an ACQUITY UPLC BEH Amide column (1.7 μm, 2.1 × 100 mm) (Waters, Milford, MA, USA). The mobile phase consisted of: (A) 20 mmol/L ammonium formate in water and (B) 20 mmol/L ammonium formate in acetonitrile. The flow rate was set at 0.25 mL/min, with an injection volume of 5 μL. The column temperature was maintained at 30 °C. The elution procedure for liquid chromatography tandem mass spectrometry was in Table 1.

#### 2.2.3. Mass Spectrum Reference Conditions

Mass spectrometric detection was conducted using an electrospray ionization (ESI) source operating in positive ion mode. Data were acquired in Multiple Reaction Monitoring (MRM) mode. The ion source temperature was set to 450 °C, with an ion spray voltage of 5500 V. The collision gas was maintained at medium intensity. The curtain gas was set at 30 psi, while both nebulizer gas and auxiliary gas were set to 50 psi. The reference conditions for liquid chromatography tandem mass spectrometry parameters are shown below (Table 2).

#### 2.2.4. Qualitative Determination and Limit of Quantification (LOQ)

Under the same instrument conditions, if the retention time of the chromatographic peak of the substance to be tested in the sample is the same as that of the standard working solution, and the relative abundance of the detected ions of the tested component in the sample mass spectrum is consistent with that of the standard, and the allowable deviation does not exceed the range specified in Table 3, it can be judged that there are corresponding amino acids in the sample.

Prepare quality control samples spiked with a calibrated mixture of amino acids at low, medium, and high concentrations to validate method linearity, repeatability, and detection limits (Table 4). The RSD value of the measured concentration is the precision. The ratio of the measured concentration to the added concentration is the accuracy. The absolute difference between the results of two independent determinations obtained under repeatability conditions shall not exceed 12% of the arithmetic mean. Each amino acid detection calibration linearity and R^2^ values are provided in Supplementary File S1, and the determination data of each amino acid content is in Supplementary Table S1 (Table S1).

### 2.3. Detection of Non-Targeted Metabolites [32]

#### 2.3.1. Reagents and Instruments

Methanol, acetonitrile (Merck, Darmstadt, Germany) and formic acid (Aladdin, Beijing, China) were used in this experiment. The instruments are used as follows: Mass spectrometer (TripleTOF 6600+, SCIEX, Foster City, CA, USA), ultra-performance liquid chromatograph (LC-30A, Kyoto City, Japan), centrifuge (5424R, Eppendorf, Hamburg, Germany), constant temperature metal mixer (MU-G02-0448, Hangzhou Miu Instrument Co., Ltd., Hangzhou, China), one hundred thousandth electronic balance (MS105DΜ, METTLER TOLEDO, Zurich, Switzerland), centrifugal concentrator (CentriVap, LABCONCO, Kansas, MO, USA), vortexers (VORTEX-5, Kyllin-Bell, Haimen, China), ultrasonic cleaner (KQ5200E, Kun Shan Ultrasonic Instruments Co., Ltd., Kunshan, China), pipette (Research plus, Eppendorf, Hamburg, Germany), automation workstation (Biomek i5, Beckman Coulter, Brea, CA, USA), sealing instrument (Mini HES, Monad, Suzhou, China).

#### 2.3.2. Dry Sample Extraction

Using vacuum freeze-drying technology, the biological samples were placed in a lyophilizer (Scientz-100F, Ningbo, China), then the samples were ground (30 Hz, 1.5 min) to powder form using a grinder (MM 400, Retsch, Haan, Germany). Next, 50 mg of sample powder were weighed using an electronic balance (MS105DΜ) and 1200 μL of pre-cooled 70% methanolic aqueous internal standard extract were added (less than 50 mg added at the rate of 1200 μL extractant per 50 mg sample). The solution was vortexed once every 30 min for 30 sec, for a total of 6 times. After centrifugation (rotation speed 12,000 rpm, 3 min), the supernatant was aspirated, and the sample was filtered through a microporous membrane (pore size: 0.22 μm) and stored in an injection vial for UPLC-MS/MS analysis.

#### 2.3.3. HPLC Conditions

All samples were acquired by the LC-MS system following the machine orders. The analytical conditions were as follows, UPLC: column, ACQUITY UPLC HSS T3 (Waters, Milford, MA, USA) 1.8 µm, 2.1 mm × 100 mm; column temperature, 40 °C; flow rate, 0.40 mL/min; injection volume, 4 μL; solvent system, water (0.1% formic acid):acetonitrile (0.1% formic acid); Sample measurements were performed with a gradient program that employed the starting conditions of 95% A, 5% B. Within 5 min, a linear gradient to 35% A, 65% B was programmed. Within 1 min, a linear gradient to 1% A, 99% B was programmed, and kept for 1.5 min. Subsequently, a composition of 95% A, 5.0% B was adjusted within 0.1 min and kept for 2.4 min (Table 5).

#### 2.3.4. MS Conditions (AB)

The data acquisition was operated using the information-dependent acquisition (IDA) mode using Analyst TF 1.7.1 Software (Sciex, Concord, ON, Canada). The source parameters were set as follows: ion source gas 1 (GAS1), 50 psi; ion source gas 2 (GAS2), 60 psi; curtain gas (CUR), 35 psi; temperature (TEM), 550 °C, or 550 °C; de-clustering potential (DP), 80 V, or −80 V in positive or negative modes, respectively; and ion spray voltage floating (ISVF), 5500 V or −4500 V in positive or negative modes, respectively. The TOF MS scan parameters were set as follows: mass range, 50–1250 Da; accumulation time, 200 ms; and dynamic background subtract, on. The product ion scan parameters were set as follows: mass range, 50–1250 Da; accumulation time, 40 ms; collision energy, 30 or −30 V in positive or negative modes, respectively; collision energy spread, 15; resolution, UNIT; charge state, 1-to-1; intensity, 100 cps; exclude isotopes within 4 Da; mass tolerance, 50 mDa; maximum number of candidate ions to monitor per cycle, 12 (Table 6).

#### 2.3.5. Data Analysis

The original data file acquired by LC-MS was converted into mzXML format by ProteoWizard software. Peak extraction, peak alignment, and retention time correction were respectively performed by the XCMS program, respectively. The “SVR” method was used to correct the peak area. The peaks with a detection rate lower than 50% in each group of samples were excluded. Identification was supported by theoretical fragment analysis, with mass accuracy deviations controlled within 25 ppm. After that, metabolic identification information was obtained by searching the laboratory’s self-built database, integrated public database, AI database, and metDNA. Finally, the substances with a comprehensive identification score of more than 0.5 and the coefficient of variation (CV) value of QC sample less than 0.3 were extracted, and then the positive and negative modes (retain the substances with the highest qualitative grade and the lowest CV value) were combined to obtain the all-sample data. The evaluation of detection data results is analyzed in Supplementary File S2, and the identified metabolites are listed in Table S2.

#### 2.3.6. PCA and Differential Metabolites Selected

Unsupervised principal component analysis (PCA) was performed by statistics function prcomp within R (www.r-project.org, accessed on 20 December 2023). The data was unit variance scaled before unsupervised PCA. For a two-group analysis, differential metabolites were identified by VIP (VIP > 1) and absolute Log2FC (|Log2FC| ≥ 1.0). VIP values were extracted from the OPLS-DA result, which also contains score plots and permutation plots, and was generated using the R package (version 4.3.2) MetaboAnalystR. The data was log-transform (log2) and mean centering before OPLS-DA (Table S2). In order to avoid overfitting, a permutation test (200 permutations) was performed.

### 2.4. RNA Extraction, Library Preparation and Sequencing

Total RNA was extracted from 22-day-after-pollination (DAP) endosperms of the Q3 F_2_ hybrid kernels (Q3-T and Q3-N), as well as from the control variety H402 (H402-T and H402-N). Each sample group consisted of three biological replicates, with each replicate derived from a pool of three endosperms collected from six independent ears. RNA extraction was performed using the TRIzol Reagent (Thermo Fisher Scientific, Waltham, MA, USA), following the manufacturer’s protocol. RNA concentration and purity were assessed using a NanoDrop 2000 spectrophotometer (Thermo Fisher Scientific, Wilmington, DE, USA), and RNA integrity was evaluated using the RNA Nano 6000 Assay Kit on the Agilent Bioanalyzer 2100 system (Agilent, Santa Clara, CA, USA). Only high-quality RNA samples (OD260/280 = 1.8~2.2, OD260/230 ≥ 2.0, RQN ≥ 6.5, 28S:18S ≥ 1.0, >1 g) were used to construct sequencing library.

For library preparation, RNA purification, reverse transcription, library construction and sequencing were performed at Hangzhou Cosmos Wisdom Biotech Co., Ltd. (Hangzhou, China) according to the manufacturer’s instructions (Illumina, San Diego, CA, USA). The RNA-seq transcriptome library was prepared following Illumina^®^ Stranded mRNA Prep Ligation from Illumina (San Diego, CA, USA) using 1 μg of total RNA. Shortly, messenger RNA was isolated according to polyA selection method by oligo(dT) beads and then fragmented by the fragmentation buffer firstly. Secondly, double-stranded cDNA was synthesized using a SuperScript double-stranded cDNA synthesis kit (Waltham, MA, USA) with random hexamer primers (Illumina). Then, the synthesized cDNA was subjected to end-repair, phosphorylation, and ‘A’ base addition according to Illumina’s library construction protocol. Libraries were size-selected for cDNA target fragments of 300 bp on 2% Low Range Ultra Agarose, followed by PCR amplification using Phusion DNA polymerase (NEB) for 15 PCR cycles. After quantified by Qubit 4.0, the paired-end RNA-seq sequencing library was sequenced with the NovaSeq X plus sequencer (2 × 150 bp read length).

### 2.5. Quality Control (QC) and Read Mapping

To ensure the accuracy of subsequent bioinformatics analysis, the raw sequencing data is first filtered to obtain high-quality sequencing data (clean data) to ensure the smooth progress of subsequent analysis. Thus, the raw paired-end reads were trimmed and quality controlled by fastp (https://github.com/OpenGene/fastp, accessed on 14 October 2024) with default parameters. The QC data was supplied in Table S3.

The clean reads were separately aligned to the reference genome (GCF_902167145.1, https://www.ncbi.nlm.nih.gov/genome/12?genome_assembly_id=838253, accessed on 14 October 2024) using HISAT software (version 2.2.1) with orientation mode. The mapped reads of each sample were assembled by StringTie (v2.2.1 release) in a reference-based approach. At the same time, evaluate the quality of the transcriptome sequencing alignment results, which mainly include sequencing saturation, gene coverage, the distribution of reads across different regions of the reference genome, and the analysis of reads distribution across different chromosomes. Only reads with a perfect match or a single mismatch were retained for further analysis and annotation.

The gene expression level is calculated by the number of reads (reads counts) of sequences located in the genome region. The expression levels of genes and transcripts abundances were quantitatively analyzed by using the RSEM software (version v1.3.3). Then, the read counts of each sample gene/transcript were obtained (Table S4).

Fragments per kilobase of transcript per million mapped reads (FPKM) and transcripts per million reads (TPM) can be used as indicators to measure the expression. Since TPM is more suitable for comparing gene expression levels between different samples, gene expression levels were then quantified using the TPM method, calculated using the following formula:

FPKM = [cDNA Fragments\over (Mapped Fragments (Millions) × Transcript Length (kb))] TPM_i_ = FPKMi/∑FPKM_j_ × 10^6^ (1)

Differential gene expression analysis between experimental groups was conducted using the DESeq2 package, which applies to a statistical model based on the negative binomial distribution to digital gene expression data. *p*-values were adjusted using the Benjamini–Hochberg method to control the false discovery rate (FDR). DEGs with |log2FC| ≧ 1, FDR ≤ 0.05, and *p*-value < 0.01 were considered to be significantly different expressed genes (DEGs) (Table S4). In addition, functional-enrichment analysis, including GO and KEGG, was performed to identify which DEGs were significantly enriched in GO terms and metabolic pathways at a Bonferroni-corrected *p*-value ≤ 0.05 compared with the whole-transcriptome background. GO functional enrichment and KEGG pathway analysis were carried out by Goatools and KOBAS, respectively. Transcription factors (TFs) were predicted from the DEG sets using the TFDB database, with TF annotation performed via HMMER-based hmmsearch, referencing the TF database PlantRegMap/PlantTFDB v5.0 (https://planttfdb.gao-lab.org/, accessed on 14 October 2024).

### 2.6. Co-Expression Correlation Analysis

For the correlation analysis, two gene expression abundance files were required, with sample headers matched one-to-one across both datasets. The expression data for reported and candidate TFs (Table S5) and their corresponding target genes (Table S6), derived from sweet kernels and waxy kernels from Q3 and H402, respectively, were used for this analysis. Bioinformatic analysis was conducted using OECloud tools (https://cloud.oebiotech.com, accessed on 12 June 2025). The resulting correlation data are provided in Table S7, along with the corresponding correlation heatmap and gene regulatory network visualization.

### 2.7. bZIPs Binding Motifs Analysis

The Plant Promoter Analysis Navigator (PlantPAN 4.0-NCKU) (https://plantpan.itps.ncku.edu.tw/plantpan4/index.html, accessed on 14 July 2025) provides an informative resource for detecting transcription factor binding sites (TFBSs). We selected the promoter regions (−3 kb upstream and 5’UTR) of genes related to the synthesis and accumulation of umami amino acids to analyze. The guide for promoter analysis of TFBS scanning in the promoter sequence was performed by follwing the insturctions in website: https://plantpan.itps.ncku.edu.tw/plantpan4/guide.html#promoter, accessed on 14 July 2025. Select transcription factors from the following species: *Brachypodium distachyon*, *Sorghum bicolor*, and *Zea mays*, The sequence similarity score of transcription factor binding motifs in the promoter region was over 75, indicating that TF binding at this site was more credible. The tandem repeat and CpNpG of the optional promoter elements were not selected. The promoter sequences were supplied in Supplementary File S3.

### 2.8. Quantitative PCR

Newly harvested kernels from Q3 (Q3-T and Q3-N) and H402 (H402-T and H402-N) were collected for RNA extraction and gene expression analysis. Total RNA was isolated using RNAiso Plus reagent (Takara, Kusatsu City, Japan), and reverse transcription was performed using reverse transcriptase (Promega) with Oligo(dT) primers to synthesize cDNA. Quantitative real-time PCR (qRT-PCR) was conducted using the StepOnePlus Real-Time PCR System (Roche, Basel, Switzerland) and PowerUp SYBR Green Master Mix (Roche), with three biological replicates and technical triplicates for each sample. The PCR cycling conditions were as follows: initial denaturation at 95 °C for 2 min; 40 cycles of 95 °C for 10 s and 65 °C for 40 s; followed by a melting curve analysis (95 °C for 15 s, 60 °C for 1 min, and 95 °C for 15 s). Tubulin was used as the internal reference gene. Relative gene expression levels were calculated using the 2^−ΔΔCt^ method. Primer sequences used for gene expression analysis and Ct values are listed in Table S8.

## 3. Results

### 3.1. Free Amino Acid Content in Kernels of Q3 and H402 Varieties

**Figure 1:**
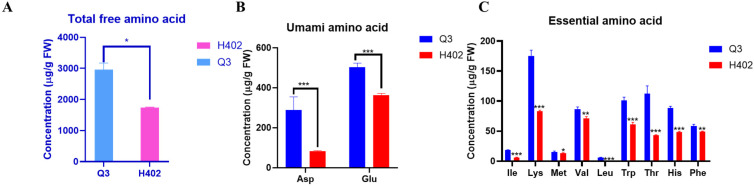
Variation in amino acid contents between Q3 and H402 corn kernels. (**A**) Total free amino acids in Q3 and H402 kernels. (**B**) Umami amino acid levels in Q3 and H402 kernels. (**C**) Essential amino acid levels in Q3 and H402 kernels. Results represent three independent experiments. Data are presented as means ± SD (*n* = 3). Statistical significance was determined by *t*-test (* *p* < 0.05, ** *p* < 0.01, *** *p* < 0.001).

Based on artificial sensory evaluation, Q3 exhibited a pronounced umami flavor after cooking. To investigate the biochemical basis of this sensory trait, the free amino acid composition of whole sweet waxy kernels was analyzed using LC-MS/MS. The analysis identified 20 types of free amino acids in Q3, with the total free amino acid content significantly higher than that of H402 (Figure 1A). In particular, the levels of the key umami-contributing amino acids, glutamate and aspartate, were markedly elevated in Q3 compared to H402. The glutamate content in Q3 kernels is 1.52 times higher than that in H402, while the aspartate is 3.32 times higher than that in H402 kernels (Figure 1B).

Corn quality is influenced not only by its flavor profile but also by its nutritional composition. Targeted analysis revealed that Q3 kernels contained all nine essential amino acids, each present at significantly higher levels than in H402. Other amino acids displayed a similar trend (Figure 1C and Figure S1A). In contrast, H402 kernels lacked detectable levels of leucine, resulting in only eight essential amino acids being identified (Figure 1C). Additionally, the crude protein content in Q3 kernels was significantly lower than that of H402 (Figure S1B), suggesting that a greater proportion of nitrogen in Q3 is retained as free amino acids. This indicates that Q3 possesses a more balanced amino acid profile, potentially enhancing its nutritional value and digestibility for human consumption.

### 3.2. Non-Targeted Metabolomic Analysis of Amino Acids in Isolated Sweet and Waxy Kernels from Hybrid Varieties

In the F_2_ generation, sweet and waxy kernels are distributed on corn cobs following Mendelian inheritance, typically at a 1:3 ratio. Quality evaluation of hybrid varieties is generally based on the overall performance of the whole kernel encompassing both sweet and waxy types. However, whether sweet and waxy kernels exhibit distinct distribution patterns or compositional differences on the same cob remains unclear. To explore this, the free amino acid content of sweet and waxy kernels was further analyzed.

**Figure 2:**
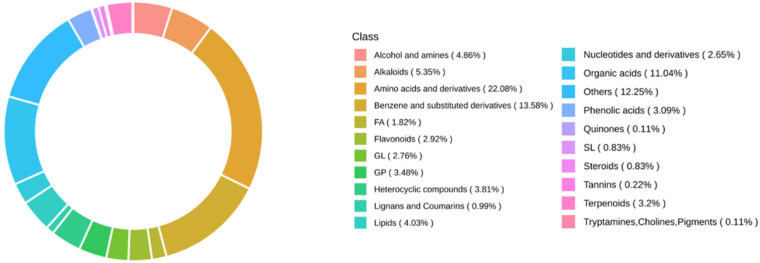
Distribution of key metabolites in sweet (T) and waxy (N) kernels revealed by non-targeted metabolomics analysis.

**Figure 3:**
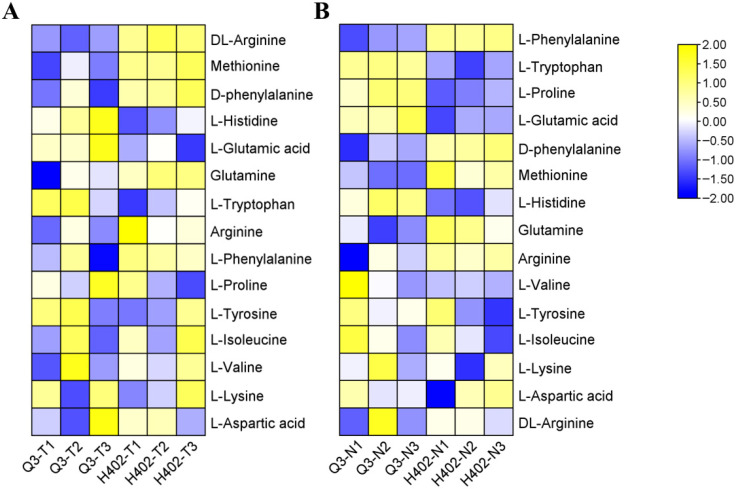
Variation in relative amino acid contents in Q3 and H402 kernels. Relative amino acid contents in sweet (**A**) and waxy (**B**) kernels of Q3 and H402.

Non-targeted metabolomic profiling revealed that protein-related metabolites accounted for the largest proportion of detected compounds, comprising up to 22.08% (Figure 2), hinting the central role of amino acid metabolism was likely involved in both kernel types. A total of fifteen amino acids were identified, with nine exhibiting Variable Importance in Projection (VIP) scores ≥1. Notably, the umami amino acid glutamate was consistently more abundant in both sweet and waxy kernels of Q3 compared to H402 (Figure 3). Furthermore, distinct accumulation patterns were observed for other amino acids (Figure 3), underscoring the differential metabolic characteristics between sweet and waxy kernels.

### 3.3. Differential Gene Expression in Sweet and Waxy Kernels

**Figure 4:**
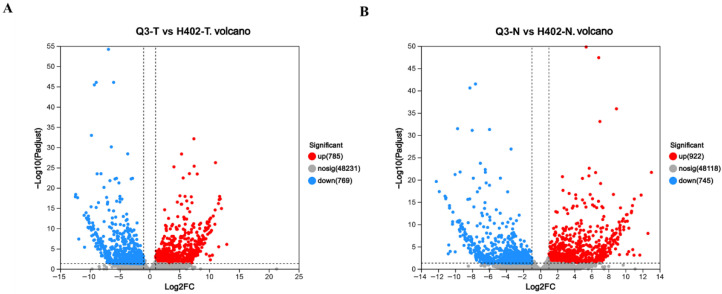
Boxline diagram and volcano plots of differentially expressed genes in kernels of Q3 and H402. (**A**) Volcano plot showing differentially expressed genes in sweet kernels (Q3-T vs. H402-T). (**B**) Volcano plot showing differentially expressed genes in waxy kernels (Q3-N vs. H402-N). Red dots represent significantly upregulated genes; blue dots represent significantly downregulated genes; grey dots indicate genes with no significant differential expression (nosig). The dashed line in the figure represents the threshold values for log2FC and-log10(Pajust).

Given the observed variation in amino acid composition among different maize kernel types across varieties, it is important to understand the underlying transcriptional regulatory mechanisms. To this end, transcriptome sequencing was performed on sweet and waxy kernels from both Q3 and H402. A total of 1554 differentially expressed genes (DEGs) were identified in sweet kernels, compared to 1667 DEGs in waxy kernels (Figure 4), suggesting that the gene regulatory network in waxy kernels may be more complex.

**Figure 5:**
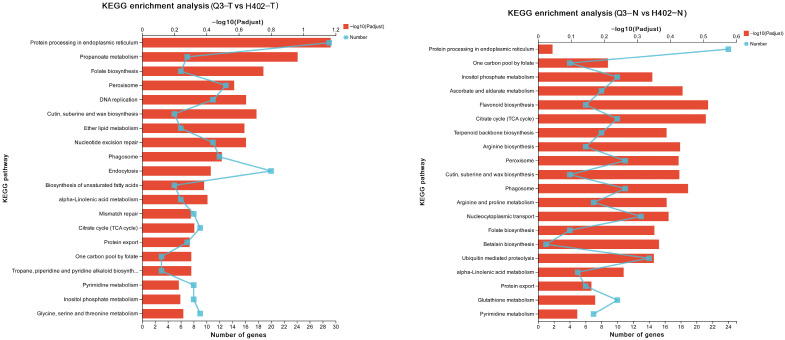
KEGG pathway enrichment analysis of differentially expressed genes in sweet kernels (Q3-T vs. H402-T) and waxy kernels (Q3-N vs. H402-N).

Subsequent KEGG pathway enrichment analysis revealed that DEGs in sweet kernels were primarily enriched in pathways related to endoplasmic reticulum-mediated protein processing. In contrast, DEGs in waxy kernels were mainly enriched in flavonoid biosynthesis and the tricarboxylic acid (TCA) cycle, with protein- and amino acid-related pathways accounting for only a small portion of the enriched categories (Figure 5). These results further support the idea that the metabolic and regulatory landscape in waxy kernels is more intricate than in sweet kernels.

### 3.4. Expression Profiles of Umami Amino Acid Synthesis Genes in Sweet and Waxy Kernels

**Figure 6:**
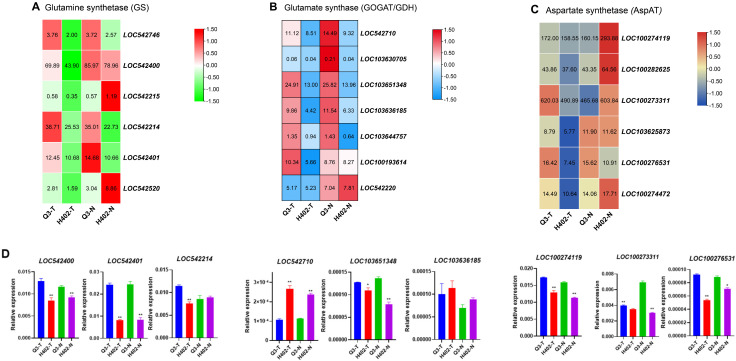
Expression of key genes involved in glutamate/glutamine and aspartate synthesis in sweet and waxy kernels of Q3 and H402. (**A**) Expression of glutamine synthetase (GS) genes in sweet and waxy kernels of Q3 and H402. (**B**) Expression patterns of glutamate synthesis-related genes (GOGAT/GDH) in sweet and waxy kernels of Q3 and H402. (**C**) Expression of aspartate aminotransferase (AspAT) genes in sweet and waxy kernels of Q3 and H402. (**D**) qPCR validation of selected genes from sets (**A**–**C**). Results represent three independent experiments. Data are shown as means ± SD (n = 3) (*t*-test, * *p* < 0.05, ** *p* < 0.01).

Given the pronounced umami flavor of cooked Q3 kernels and prior reports highlighting the critical roles of *GS*, *GOGAT*, and *AspAT* in umami amino acid biosynthesis, these genes were selected for detailed analysis. Transcriptome sequencing identified 19 amino acid synthesis-related genes that were differentially expressed between sweet and waxy kernels of Q3 and H402 (Figure 6). Then, the highly expressed genes were chosen for validation via quantitative PCR (qPCR).

The qPCR results confirmed that the expression of *GS* genes *LOC542400* and *LOC542401* was significantly higher in Q3 than H402, both in sweet and waxy kernels. For another gene *LOC542214*, it showed significantly higher expression in Q3 sweet kernels (Q3-T) than H402-T, and no significant expression differences were observed between the waxy kernels (Q3-N vs. H402-N) (Figure 6A). For *GOGAT* genes, qPCR data aligned well with transcriptome profiles for *LOC103651348*, but discrepancies were observed for *LOC542710* and *LOC103636185*, their expression trend was inconsistent with transcriptome profiles (Figure 6B,D). For *AspAT* genes, qPCR confirmed that the expression levels of *LOC100274119*, *LOC100273311*, and *LOC100276531* were all significantly higher in Q3 than in H402 of both sweet and waxy kernels, which is consistent with transcriptome data (Figure 6C,D).

### 3.5. Protein Translation Related Genes in Sweet and Waxy Kernels

**Figure 7:**
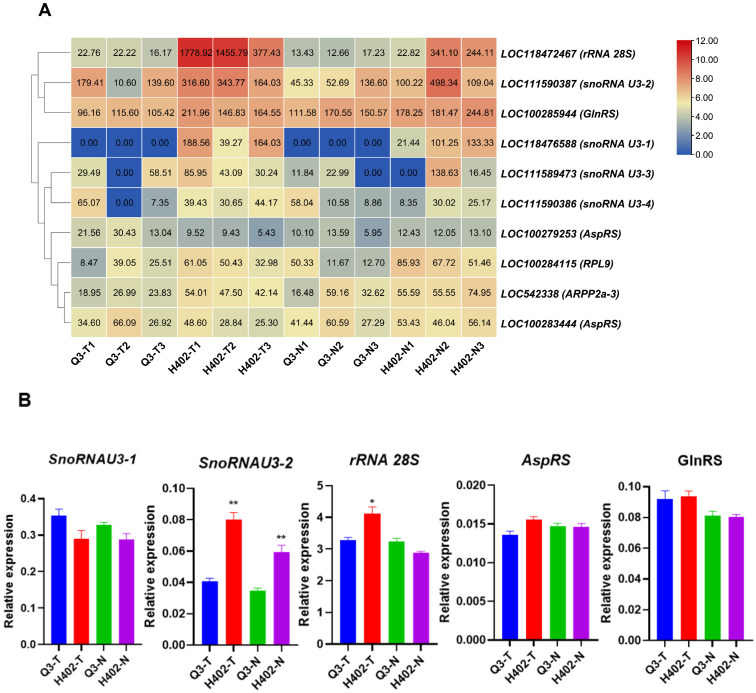
Expression of translation-related genes in sweet and waxy kernels of Q3 and H402. (**A**) Expression profiles of protein translation-related genes in sweet kernels (Q3-T and H402-T) and waxy kernels (Q3-N and H402-N). (**B**) qPCR validation of selected gene expressions. Results represent three independent experiments. Data are presented as means ± SD (n = 3) (*t*-test, * *p* < 0.05, ** *p* < 0.01).

The dynamic balance between protein synthesis and free amino acid pools represents a key regulatory mechanism in plant protein metabolism, where both processes mutually influence each other. Transcriptomic analysis revealed differential expression of four small nucleolar RNA U3 (snoRNA U3) genes, ribosome-related genes, and two aminoacyl-tRNA synthetase genes between H402 and Q3 (Figure 7A). qPCR results confirmed that *snoRNA U3-2* and *rRNA 28S* expression levels were significantly lower in Q3-T than in H402-T kernels, which was consistent with the transcriptomic data. Additionally, expression of two aminoacyl-tRNA genes, *AspRS* and GlnRS, showed no significant difference between the two varieties in either kernel type (Figure 7B).

### 3.6. Transcription Factors Regulatory Network

**Figure 8:**
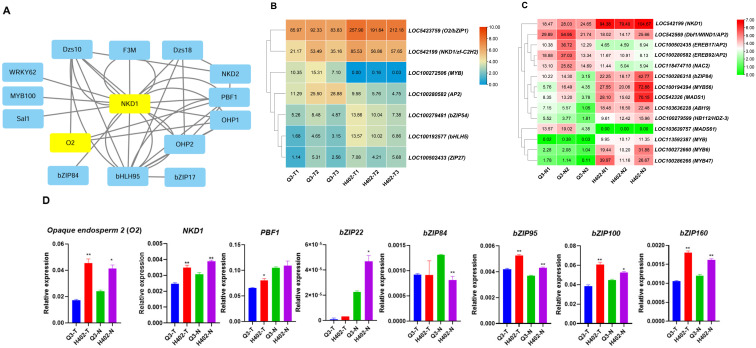
Transcription factor networks in sweet and waxy kernels of Q3 and H402. (**A**) Predicted transcription factor regulatory network based on STRING database analysis. (**B**) Differential expression of transcription factor genes in sweet kernels (Q3-T vs. H402-T). (**C**) Differential expression of transcription factor genes in waxy kernels (Q3-N vs. H402-N). (**D**) qPCR validation of selected genes from sets B and C. Results represent three independent experiments. Data are presented as means ± SD (n = 3) (*t*-test, * *p* < 0.05, ** *p* < 0.01).

To investigate the upstream regulatory pathways of umami amino acid synthetase genes, an association network between TFs and their target genes was constructed using the STRING database, focusing on TFs known to be involved in protein synthesis (Figure 8A). The analysis identified *O2* and *NKD1* as potential core regulators within this network (Figure 8A). Transcriptome data showed that the number of differentially expressed TFs is lower in sweet kernels than in waxy kernels, with distinct expression patterns suggesting a more complex regulatory mechanism in waxy kernels (Figure 8B,C). qPCR results validation showed that *O2*, *NKD1*, *bZIP95*, *bZIP100*, and *bZIP160* were indeed significantly upregulated in kernels of H402 (H402-Tand H402-N) compared to Q3 (Q3-T and Q3-N). Some of above genes expression trends were also observed in transcriptome data. *FBP1* expression level was only significantly higher in H402-T than in Q3-T, and there was no expression difference between kernels of Q3-N and H402-N. Additionally, *bZIP22* and *bZIP84* exhibited no significant difference expression between H402-T and Q3-T, but showed opposite expression trend between H402-N and Q3-N (Figure 8D). These results imply that *bZIP* transcription factors may interact with *NKD1* and *O2* to regulate downstream gene expression, thereby modulating protein synthesis and influencing free amino acid accumulation.

**Figure 9:**
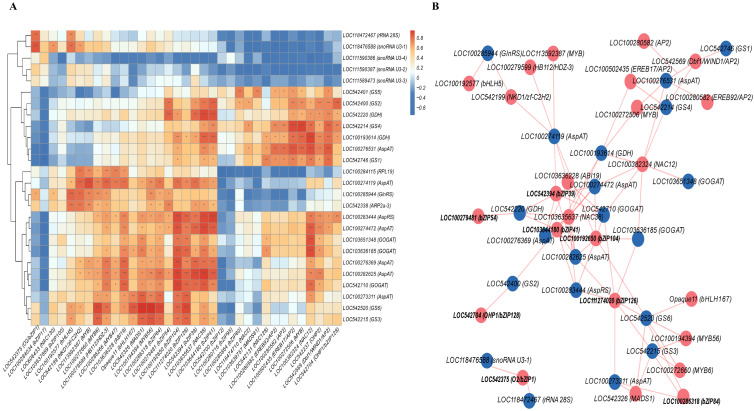
Correlation analysis between transcription factors and potential target genes in sweet and waxy kernels of Q3 and H402. (**A**) Correlation heatmap illustrating relationships between transcription factors and probable target genes in sweet and waxy kernels of Q3 and H402. (**B**) Network map depicting interactions between transcription factors and likely target genes in sweet and waxy kernels of Q3 and H402. (Data are presented as means ± SD (n = 3) (*t*-test, * *p* < 0.05, ** *p* < 0.01, *** *p* < 0.001).

*O2*, a distinctive member of the *bZIP* family, along with nucleolar small RNAs and 28S ribosomal RNA, plays a vital role in the protein synthesis machinery. Correlation analysis revealed that *O2* and seven *bZIPs* positively correlate with *snoRNA U3-2*, *AspAT*, *GOGAT*, *GS*, and *rRNA 28S* expression (Figure 9), while three other *bZIPs* showed negative correlations in both sweet and waxy kernels (Figure 9A). Additionally, other TF families such as *NACs*, *bHLHs*, and *MYBs* also displayed positive correlations with these target genes (Figure 9B). Promoter sequence analysis of key umami amino acid synthesis genes, along with *snoRNA U3-2* and *rRNA 28S* genes, uncovered numerous *bZIP* binding motifs. Given the prevalence of these motifs, it is proposed that *O2* and other *bZIPs* may directly regulate the expression of these target genes, thereby modulating protein and amino acid biosynthesis (Table 7).

## 4. Discussion

It is well known that most amino acids contribute to distinct flavors, adding complexity to food taste profiles [33,34]. The quality of fresh sweet waxy maize is typically assessed using F2 seeds derived from self-pollination of the F1 generation. However, since F2 maize ears exhibit a mixture of different traits, quality evaluation generally considers the ears with sweet and waxy kernels. Current research on the quality formation of F2 maize kernels largely focuses on analyzing the parent lines and F2 varieties, with few studies examining sweet and waxy kernels separately within the F2 generation at milky stage. In this study, the overall quality of fresh maize kernels was first evaluated, followed by separate sampling of sweet and waxy kernels for detailed transcriptomic and metabolomic analyses.

This study demonstrated that Q3 kernels contain a higher total amount of free amino acids compared to the control variety H402, with particularly elevated levels of the umami amino acids glutamate (Glu) and aspartate (Asp) (Figure 1B), which contribute to Q3′s distinctive umami flavor. Numerous studies have highlighted the importance of amino acids in maintaining nitrogen balance in the human body, playing a vital role in health maintenance and chronic disease prevention [35,36]. Fresh corn is rich in free amino acids and they can be directly absorbed by the human body [37]. In addition to essential amino acids, fresh corn also contains significant amounts of semi-essential amino acids such as arginine and histidine, as well as umami and sweet-tasting amino acids (Figure 1 and Figure S1). Consequently, the free amino acid content in fresh corn has attracted increasing attention from breeders. Targeted analysis revealed that Q3 kernels have a more comprehensive amino acid profile, containing 20 types of free amino acids, whereas H402 kernels have only 19, lacking leucine (Figure 1 and Figure S1). Notably, Q3 kernels contain all nine essential amino acids (Figure 1C), making this variety especially beneficial for children’s nutrition and development. Therefore, Q3 corn holds promise as a healthy, nutritious food to support growth in children. These indicate that Q3 contains a more comprehensive and balanced amino acid composition, which is conducive to human absorption and thus regulates human health.

During the filling stage of fresh kernels, the synthesis of primary metabolites is highly active and vigorous [38]. Currently, large amounts of sugars and free amino acids are produced, serving as substrates for protein and polypeptide synthesis [39]. Consequently, differential gene expressions during this stage may lead to variations in metabolite levels, including differences in the accumulation of umami amino acids. Glutamate, a key umami amino acid, comprises the largest proportion of total protein in corn kernels, and it is especially concentrated in the main storage proteins [40]. As a central molecule in nitrogen metabolism, glutamate participates in the biosynthesis of other amino acids such as proline and arginine and significantly influences kernel flavor [3]. Serving as a precursor for essential amino acids like lysine and threonine, aspartate plays a crucial role in nitrogen transport and energy metabolism [41]. Previous studies have shown that glutamate synthesis primarily occurs through the *GDH* and *GS-GOGAT* pathways [42], while aspartate synthesis is mainly driven by *aspartate kinase* (*AK*) and *AspAT* [43]. In this study, these genes were found to be highly expressed in both sweet and waxy kernels of Q3, correlating with the elevated levels of umami amino acids observed in Q3 kernels. Similarly, the essential amino acids synthesis genes expression trends are also consistent with their accumulation traits in Q3 and H402 kernels (Figure S2).

Meanwhile, free amino acids are generally not converted into secondary metabolites but are primarily utilized for protein synthesis, which results in a decline in free amino acid accumulation during the endosperm filling stage. It was observed that *snoRNA U3* and *ribosomal RNA* gene are differentially expressed between Q3 and H402 kernels. The downregulation of these genes may reduce protein synthesis efficiency, thereby lowering the utilization of free amino acids and causing their accumulation.

Studies have shown that the transcription factor *O2*, a core gene specifically expressed in the endosperm, interacts with several other transcription factors such as *Pbf1*, *NAC128*, *NAC130*, and *bZIP22* to jointly regulate maize storage protein synthesis [27,29,30]. Mutations in these genes lead to the accumulation of lysine and alter the levels of 16 other amino acids. Quantitative PCR validation revealed that these genes were significantly downregulated in both sweet and waxy kernels of Q3, while their expression was higher in H402, although transcriptome data did not show statistically significant differences. Notably, *NAC128* and *bZIP22*, which interact with *O2*, exhibited expression patterns similar to *O2* (Figure 8 and Figure S3). The downregulation of these genes likely reduces maize protein synthesis, resulting in an accumulation of free amino acids. In *O2* maize mutants, levels of 17 free amino acids change, with some increasing and others decreasing. However, in Q3 kernels where *O2* is suppressed, nearly all amino acids except glutamine showed significant accumulation. This suggests that, in addition to *O2*, other regulatory pathways may influence free amino acid accumulation.

Furthermore, beyond *O2* as a key *bZIP* family member, many other *bZIPs* are simultaneously expressed lowly in Q3 maize kernels compare to H402, according to transcriptome analysis. Interestingly, studies in Arabidopsis found that the knockout of *AtbZIP63* led to an increase in almost all amino acid levels, while its overexpression caused decreases [44]. Another study in 2019 revealed that *bZIP53* affects amino acid metabolism in Arabidopsis [45,46], although the specific target genes were not examined. *bZIP11* affects amino acid metabolism by regulating the expression of asparagine synthetase1 [47]. The latest research found a *bZIP TF* (*ChCpc1*) mediates amino acid synthesis and autophagy in maize [48]. An increasing number of studies suggest that the *bZIP* family may have a significant regulatory role in controlling amino acid synthesis. Based on correlation analysis, it is proposed that *bZIPs* may negatively regulate transcription by binding to promoters of key amino acid synthesis genes or positively regulate ribosomal RNA genes, thereby influencing amino acid synthesis and accumulation in maize.

In summary, the accumulation of free umami amino acids in kernels is a finely balanced process involving multiple gene regulatory networks. The increase in these amino acids is directly driven by key synthesis genes such as *GS*, *GOGAT*, *GDH*, and *AspAT*. Indirectly, genes like *snoRNA U3-2* and *rRNA 28S* may influence protein translation efficiency, thereby affecting protein formation. Transcription factors including *O2*, *bZIPs*, and *NACs* are known to regulate maize protein genes, suggesting their potential role in amino acid accumulation. Further analysis revealed that the promoter regions of glutamate/aspartate synthesis genes, as well as snoRNA U3 and rRNA 28S, contain multiple *bZIP* binding elements (Table 7). Notably, *O2*, a key member of the *bZIP* family, along with other TFs such as *NAC130*, *NKD1*, *NKD2*, *PBF1*, and *bZIPs*, likely play critical roles in regulating gene expression and amino acid synthesis. The presence of numerous *bZIP* binding sites across these important target genes underscores the complexity of their regulatory interactions involving *snoRNA U3*-*2*, *rRNA 28S*, *GS*, *GDH*, *GOGAT*, and *AspAT*.

## 5. Conclusions

This study is the first to reveal the underlying mechanisms by which the transcription factors *O2* and *bZIPs* may play a positive and central role in free amino acid accumulation in isolated sweet and waxy maize kernels, particularly through protein synthesis. *bZIPs* may enhance free amino acid levels by directly binding to the promoters of key synthesis genes such as *GS*, *GOGAT*, *GDH*, and *AspAT*, thereby upregulating their expression. Additionally, the protein synthesis-related non-coding genes *snoRNA U3-2* and *rRNA 28S* were identified as potential targets of *bZIP* regulation, indirectly promoting amino acid accumulation.

In summary, this research not only uncovers likely mechanisms by which bZIPs regulate free amino acid synthesis and metabolism in maize kernels but also expands the known regulatory network of O2 and the bZIP family. These findings provide valuable genetic resources for improving maize flavor and quality, and offer new avenues for molecular breeding in maize and other crops.

## Figures and Tables

**Table 1:** Liquid chromatography tandem mass spectrometry elution procedure.

Time (min)	A (%)	B (%)
0	95	5
5	35	65
6	1	99
7.5	1	99
7.6	95	5
10	95	5

**Table 2:** Acquisition parameter.

Number	Parent Ion	Daughter Ion	Dwell Time (ms)	Amino Acids	DP	CE
1	132.1	86.4	50	Ile-1	40	14
2	132.1	103.8	50	Ile-2	40	11
3	133.9	74.1	50	Asp-1	30	19
4	133.9	88.0	50	Asp-2	40	13
5	147.1	84.3	50	Lys-1	30	22
6	147.1	130.2	50	Lys-2	17	14
7	106.2	60.2	50	Ser-1	23	16
8	106.2	88.2	50	Ser-2	6	13
9	150.1	132.9	50	Met-1	33	13
10	150.1	104.3	50	Met-2	34	15
11	147.1	130.1	50	Gln-1	29	14
12	147.1	84.1	50	Gln-2	24	23
13	118.1	72.1	50	Val-1	23	15
14	118.1	89.7	50	Val-2	23	9
15	116.2	70.1	50	Pro-1	34	21
16	116.2	59.9	50	Pro-2	34	15
17	90.8	65.0	50	Ala-1	234	26
18	90.8	72.9	50	Ala-2	94	12
19	148.2	84.2	50	Glu-1	15	20
20	148.2	102.1	50	Glu-2	20	14
21	132.1	85.9	50	Leu-1	19	14
22	132.1	104.1	50	Leu-2	53	10
23	76.0	30.0	50	Gly-1	40	14
24	76.0	46.0	50	Gly-2	40	17
25	175.2	70.1	50	Arg-1	32	26
26	175.2	116.2	50	Arg-2	50	19
27	205.1	188.3	50	Trp-1	16	15
28	205.1	86.7	50	Trp-2	20	14
29	182.1	165.1	50	Tyr-1	35	14
30	182.1	136.2	50	Tyr-2	20	19
31	120.1	102.1	50	Thr-1	35	12
32	120.1	74.0	50	Thr-2	32	15
33	156.0	110.1	50	His-1	41	20
34	156.0	95.2	50	His-2	25	21
35	166.1	120.1	50	Phe-1	44	18
36	166.1	148.9	50	Phe-2	41	13
37	133.1	87.2	50	Asn-1	38	14
38	133.1	116.0	50	Asn-2	49	14
39	241.1	152.0	50	Cys-1	33	17
40	241.1	120.1	50	Cys-2	33	24

Note: Amino acid-1: quantitative ion pair, amino acid-2: auxiliary ion pair. DP: de-clustering potential, CE: collision energy.

**Table 3:** Maximum allowable deviation of relative ion abundance during qualitative determination.

Relative ion abundance/%	>50	>20–50	>20–50	≤10
Allowable relative deviation/%	±20	±25	±30	±50

**Table 4:** Information of amino acids and amino acid limit of quantification.

Amino Acid		CAS No.	Molecular Weight	LOQ (μg/kg)
Alanine	Ala	56-41-7	89.09	25
Arginine	Arg	1119-34-2	174.2	12.5
Asparagine	Asn	70-47-3	132.12	50
Aspartic acid	Asp	56-84-8	133.1	50
Cystine	Cys	56-89-3	240.3	50
Glutamine	Gln	56-85-9	146.15	25
Glutamic acid	Glu	56-86-0	147.13	25
Glycine	Gly	56-40-6	75.07	50
Histidine	His	5934-29-2	155.16	12.5
Isoleucine	Ile	73-32-5	131.18	12.5
Leucine	Leu	61-90-5	131.18	12.5
Lysine	Lys	657-27-2	146.19	25
Methionine	Met	63-68-3	149.2	12.5
Phenylalanine	Phe	63-91-2	165.19	12.5
Proline	Pro	147-85-3	115.13	12.5
Serine	Ser	56-45-1	105.09	25
Threonine	Thr	72-19-5	119.13	25
Tyrosine	Tyr	60-18-4	181.19	25
Valine	Val	72-18-4	117.15	12.5
Tryptophan	Trp	73-22-3	204.23	25

**Table 5:** Gradient conditions of mobile phase in T3 column.

Time (min)	A (%)	B (%)
0	95	5
5	35	65
6	1	99
7.5	1	99
7.6	95	5
10	95	5

**Table 6:** AB TripleTOF 6600 mass spectrometry conditions.

	ESI+	ESI−
Duration (min)	10	10
IonSpray Voltage (V)	5000	−4000
Temperature (°C)	550	550
Ion Source Gas1 (psi)	50	50
Ion Source Gas2 (psi)	60	60
Curtain Gas (psi)	35	35
De-clustering Potential (V)	60	−60
MS1 Collision Energy (V)	10	−10
MS2 Collision Energy (V)	30	−30
Collision Energy Spread (V)	15	15
MS1 TOF Masses (Da)	50~1250	50~1250
MS2 TOF Masses (Da)	25~1250	25~1250

**Table 7:** Number of bZIP binding motifs within the promoter regions of their target genes.

Genes IDs	Binding Motifs Numbers (*bZIPs*)	Gene Annotations
*LOC542214/Zm00001eb253820*	25	glutamine synthetase 4(gln4)
*LOC542401/Zm00001eb190340*	32	glutamine synthetase 5 (gln5)
*LOC103651348/Zm00001eb156610*	19	glutamate synthase2/glutamine oxoglutarate aminotransferase2 (GS2/gogat2)
*LOC542710/Zm00001eb329710*	26	ferredoxin-dependent glutamate synthase (Fd-gogat)
*LOC100274119/Zm00001eb238900*	20	aspartate aminotransferase (AspAT)
*LOC100273311/Zm00001eb152450*	17	glutamate-oxaloacetate transaminase 1 (got1)
*LOC100276531/Zm00001eb146400*	17	glutamate-oxaloacetate transaminase 4 (gogat4)
*LOC111590387*	12	small nucleolar RNA U3-2
*LOC11847246*	23	28S ribosomal RNA

## Data Availability

The original contributions presented in the study are included in the article/Supplementary Materials; further inquiries can be directed to the corresponding authors.

## Supplementary Materials

The following supporting information can be downloaded at: https://www.mdpi.com/article/10.3390/foods14213628/s1, Figure S1: Additional free amino acids (A) and crude protein (B) content in maize kernels of Q3 and H402; Figure S2: Essential amino acids synthesis genes in maize kernels of Q3 and H402; Figure S3: bZIP and reported TFs expression profiles in sweet and waxy kernels of Q3 and H402 (A) bZIP expression profiles and reported TFs expression in sweet and waxy kernels of Q3 and H402. (B) qPCR validation of reported TFs expression in sweet and waxy kernels of Q3 and H402.; Table S1: Amino acid content in Q3-T, Q3-N, H402-T, and H403-N; Table S2: All metabolites summary; Table S3: QC data statistics; Table S4: Read counts and TPM of each sample gene/transcript level and different expressed genes (DEGs). Table S5: Reported and concerned TFs expression file in sweet kernels and waxy kernels from Q3 and H402.; Table S6: Reported and concerned TFs targets expression file in sweet kernels and waxy kernels from Q3 and H402; Table S7: Correlation data between TFs and their targets; Table S8: Primers used in this study and CT values. Supplementary File S1: R-square value of each amino acid detection regression equation; Supplementary File S2: Results evaluation of non-targeted metabolites; Supplementary File S3: bZIP target genes promoter sequences.
