# Supplementary material for: Regulatory Mechanisms of Free Umami Amino Acid Accumulation in Fresh Waxy Kernels: Insights from Transcriptome and Metabolomics Analyses
Source: Foods. 2025 Oct 24;14(21):3628. doi: 10.3390/foods14213628 (PMC12609758; doi:10.3390/foods14213628)

Analyte Name: Ile-1
Internal Standard: N/A

**Data File**	Data20231209-YT.wiff	**Result Table**	20231214-YT
**Acquisition Date**	12/10/2023 2:30:36 PM	**Algorithm Used**	MQ4
**Acquisition Method**	20231209-YT.dam	**Instrument Name**	QTRAP 6500+ Low Mass
**Project**	Amino Acid\AA		

Regression Equation: y = 4.79580e9 x + 1.50860e7 (R^2= 0.99283)

Expected Concentration	Number of Values	Mean Calculated Concentration	% Accuracy	Std. Deviation	%CV
0.0078125	1 of 1	5.793e-3	74.2	N/A	N/A
0.0156250	1 of 1	1.545e-2	98.9	N/A	N/A
0.0312500	1 of 1	3.499e-2	112.0	N/A	N/A
0.0625000	1 of 1	7.221e-2	115.5	N/A	N/A
0.1250000	1 of 1	1.349e-1	108.0	N/A	N/A
0.2500000	1 of 1	2.288e-1	91.5	N/A	N/A

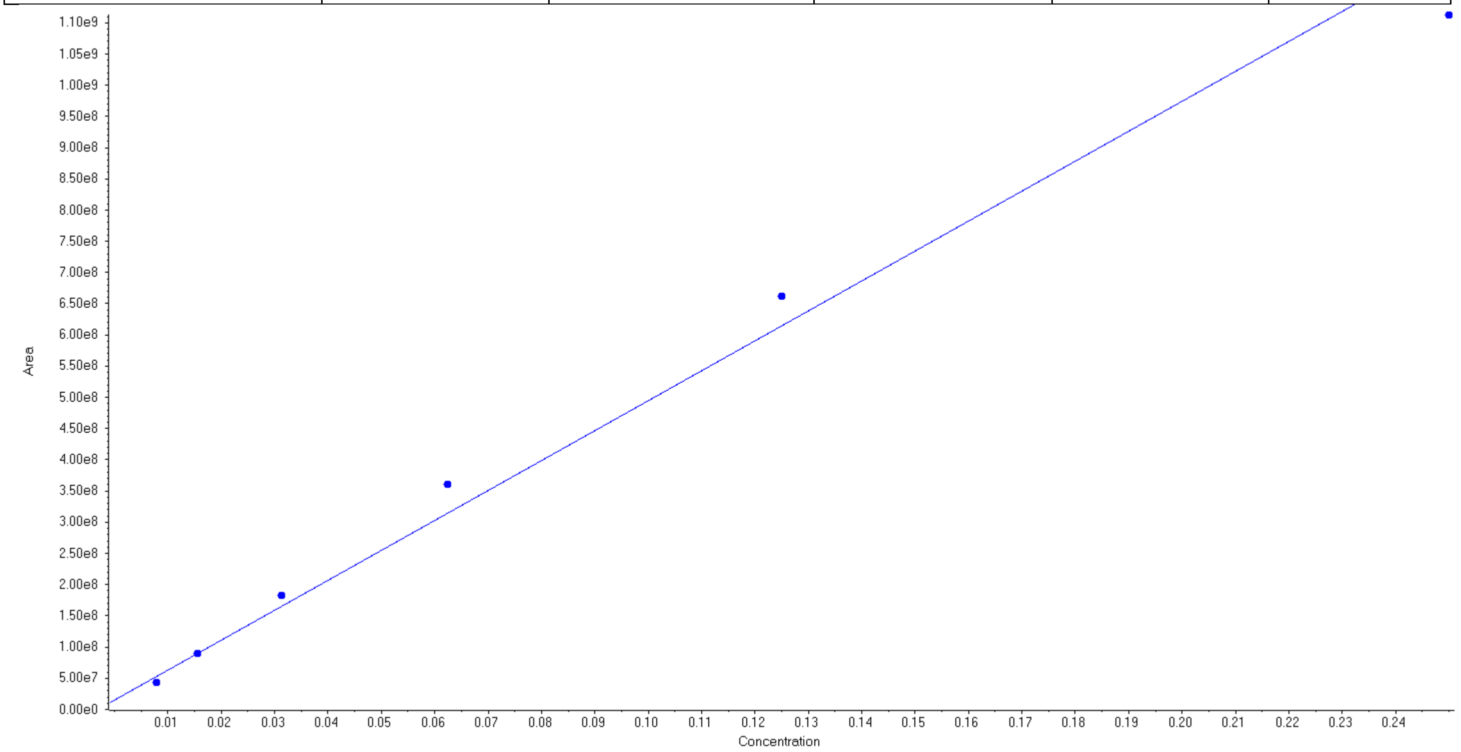

Analyte Name: Asp-1
Internal Standard: N/A

Data File	Data20231209-YT.wiff	Result Table	20231214-YT
Acquisition Date	12/10/2023 2:30:36 PM	Algorithm Used	MQ4
Acquisition Method	20231209-YT.dam	Instrument Name	QTRAP 6500+ Low Mass
Project	Amino Acid\AA		

Regression Equation: y = 7.01097e8 x + -1.23903e6 (R^2 = 0.99785)

Expected Concentration	Number of Values	Mean Calculated Concentration	% Accuracy	Std. Deviation	%CV
0.0078125	1 of 1	9.121e-3	116.8	N/A	N/A
0.0156250	1 of 1	1.533e-2	98.1	N/A	N/A
0.0312500	1 of 1	2.852e-2	91.3	N/A	N/A
0.0625000	1 of 1	5.884e-2	94.2	N/A	N/A
0.1250000	1 of 1	1.189e-1	95.1	N/A	N/A
0.2500000	1 of 1	2.615e-1	104.6	N/A	N/A

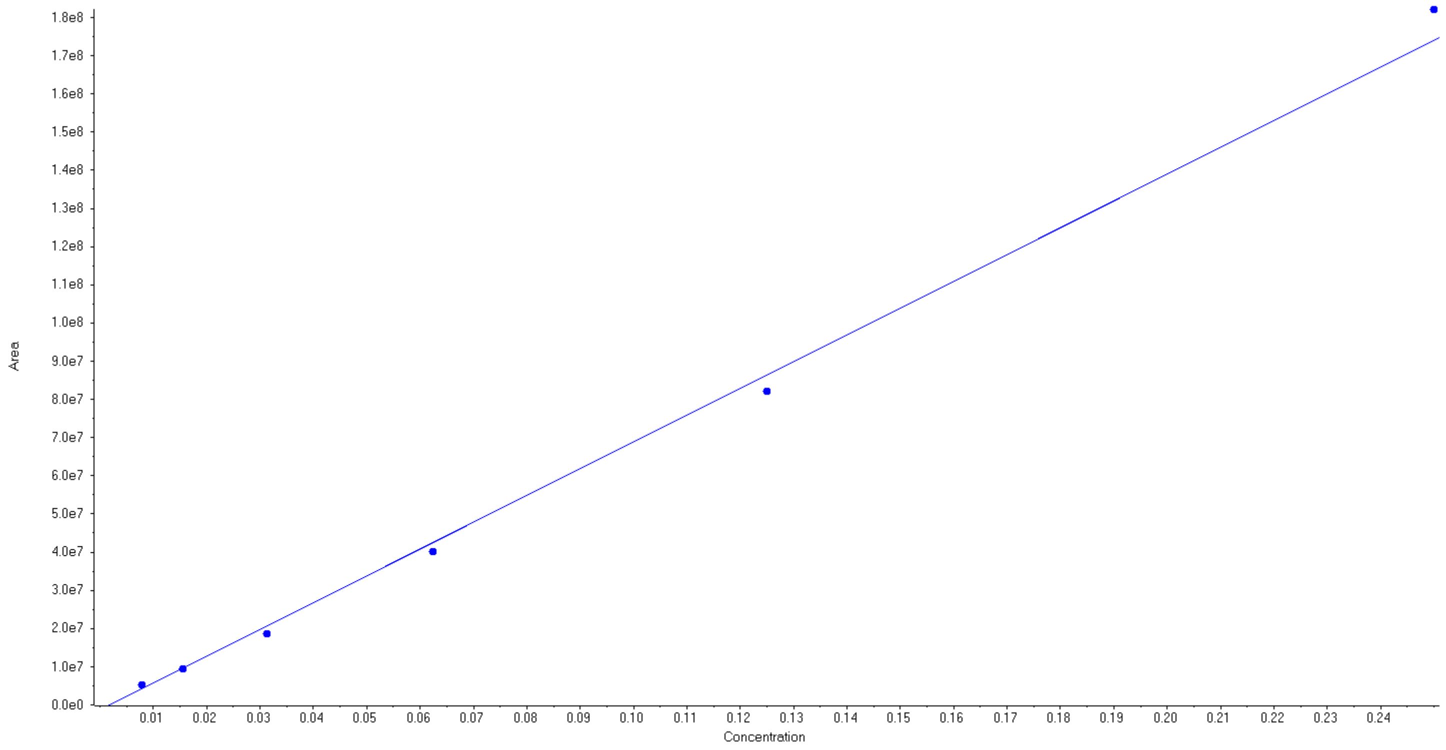

Analyte Name: Lys-1
Internal Standard: N/A

**Data File**	Data20231209-YT.wiff	**Result Table**	20231214-YT
**Acquisition Date**	12/10/2023 2:30:36 PM	**Algorithm Used**	MQ4
**Acquisition Method**	20231209-YT.dam	**Instrument Name**	QTRAP 6500+ Low Mass
**Project**	Amino Acid\AA		

Regression Equation: $y = 5.38883e9\ x + 3.45095e6\ (R^2 = 0.99163)$

Expected Concentration	Number of Values	Mean Calculated Concentration	% Accuracy	Std. Deviation	%CV
0.0078125	1 of 1	6.818e-3	87.3	N/A	N/A
0.0156250	1 of 1	1.416e-2	90.6	N/A	N/A
0.0312500	1 of 1	3.258e-2	104.3	N/A	N/A
0.0625000	1 of 1	7.175e-2	114.8	N/A	N/A
0.1250000	1 of 1	1.407e-1	112.5	N/A	N/A
0.2500000	1 of 1	2.262e-1	90.5	N/A	N/A

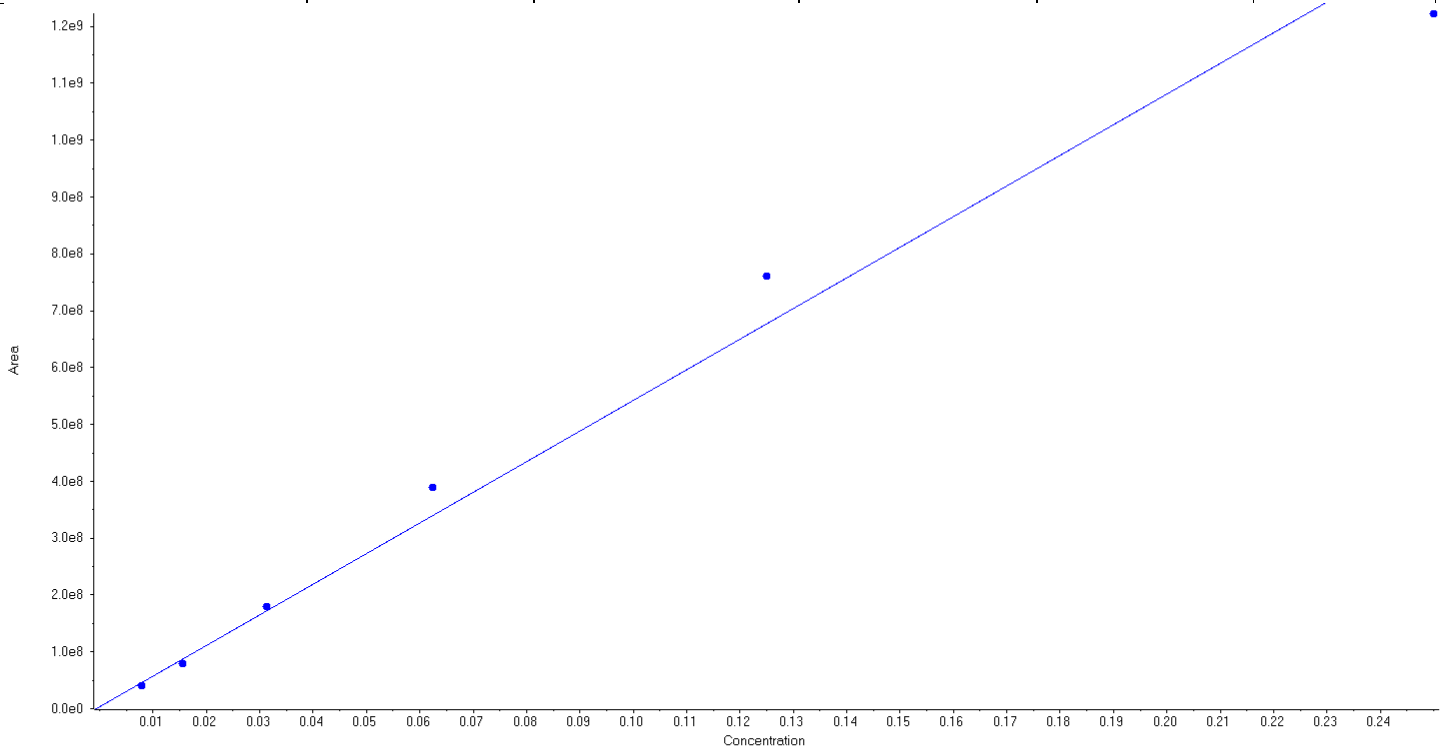

Analyte Name: Ser-1
Internal Standard: N/A

**Data File**	Data20231209-YT.wiff	**Result Table**	20231214-YT
**Acquisition Date**	12/10/2023 2:30:36 PM	**Algorithm Used**	MQ4
**Acquisition Method**	20231209-YT.dam	**Instrument Name**	QTRAP 6500+ Low Mass
**Project**	Amino Acid\AA		

Regression Equation: $y = 5.63852e8\ x + 8.76218e5\ (R^2 = 0.99847)$

Expected Concentration	Number of Values	Mean Calculated Concentration	% Accuracy	Std. Deviation	%CV
0.0078125	1 of 1	9.545e-3	122.2	N/A	N/A
0.0156250	1 of 1	1.314e-2	84.1	N/A	N/A
0.0312500	1 of 1	2.858e-2	91.5	N/A	N/A
0.0625000	1 of 1	6.351e-2	101.6	N/A	N/A
0.1250000	1 of 1	1.242e-1	99.4	N/A	N/A
0.2500000	1 of 1	2.532e-1	101.3	N/A	N/A

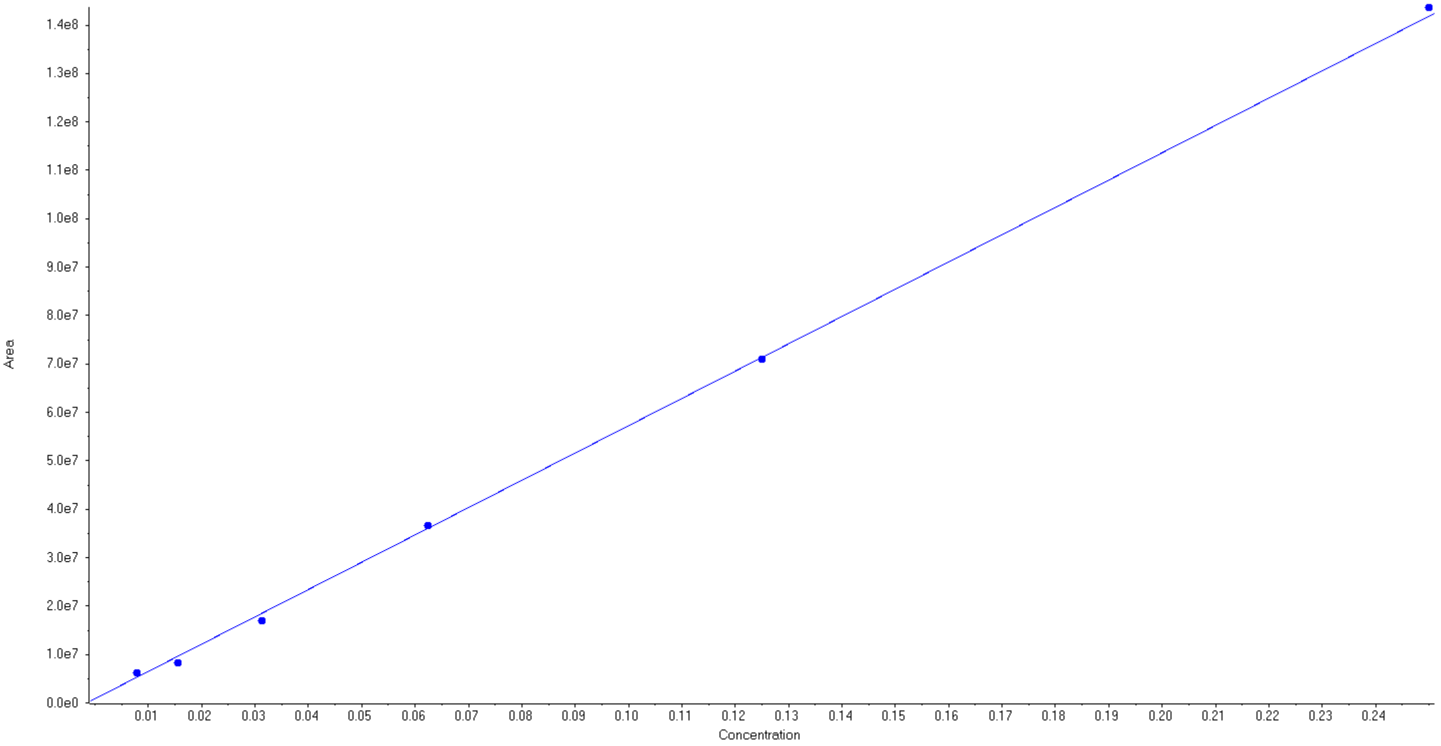

Analyte Name: Met-1
Internal Standard: N/A

**Data File**	Data20231209-YT.wiff	**Result Table**	20231214-YT
**Acquisition Date**	12/10/2023 2:30:36 PM	**Algorithm Used**	MQ4
**Acquisition Method**	20231209-YT.dam	**Instrument Name**	QTRAP 6500+ Low Mass
**Project**	Amino Acid\AA		

Regression Equation: y = 1.92922e9 x + 1.99293e6 (R^2= 0.99948)

Expected Concentration	Number of Values	Mean Calculated Concentration	% Accuracy	Std. Deviation	%CV
0.0078125	1 of 1	6.932e-3	88.7	N/A	N/A
0.0156250	1 of 1	1.464e-2	93.7	N/A	N/A
0.0312500	1 of 1	2.857e-2	91.4	N/A	N/A
0.0625000	1 of 1	6.475e-2	103.6	N/A	N/A
0.1250000	1 of 1	1.299e-1	103.9	N/A	N/A
0.2500000	1 of 1	2.474e-1	99.0	N/A	N/A

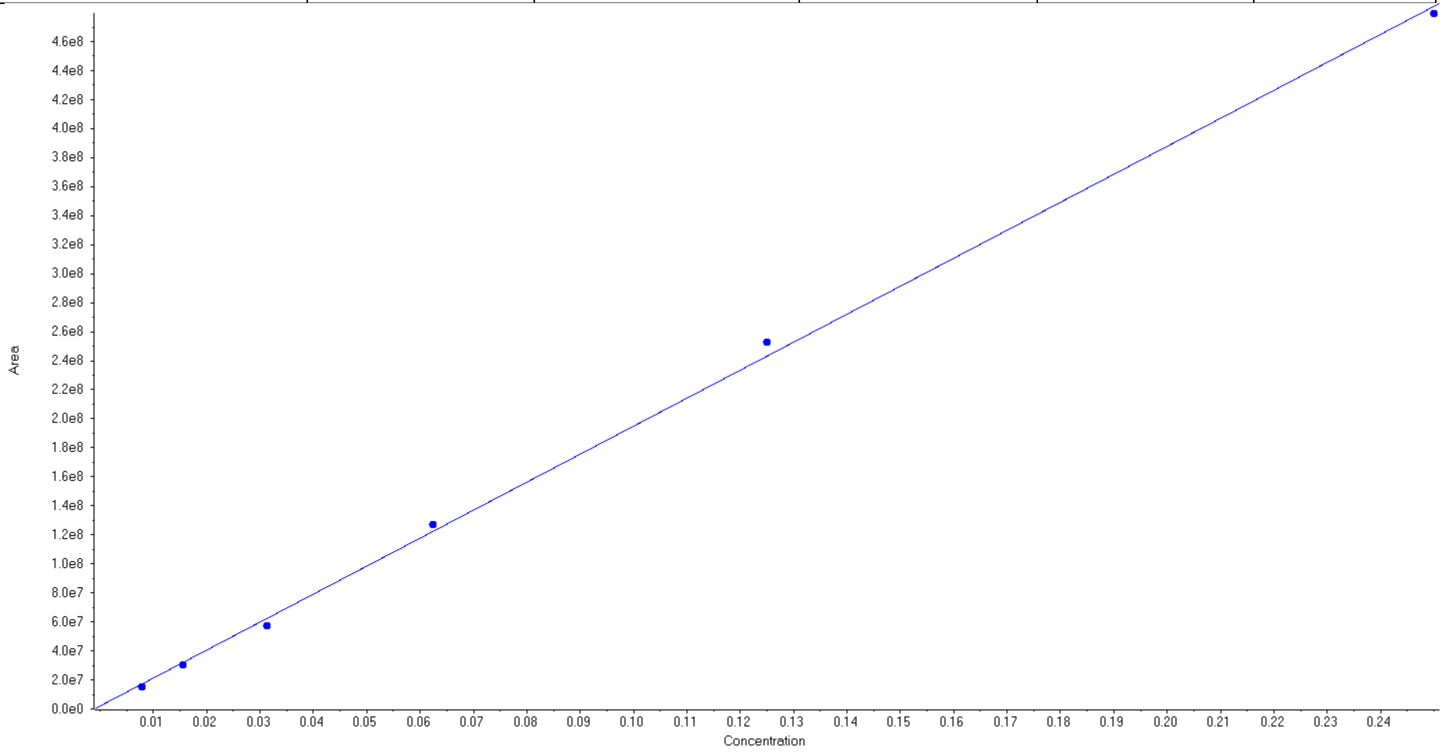

Analyte Name: Gln-1
Internal Standard: N/A

**Data File**	Data20231209-YT.wiff	**Result Table**	20231214-YT
**Acquisition Date**	12/10/2023 2:30:36 PM	**Algorithm Used**	MQ4
**Acquisition Method**	20231209-YT.dam	**Instrument Name**	QTRAP 6500+ Low Mass
**Project**	Amino Acid\AA		

Regression Equation: y = 1.90726e9 x + -6.79365e6 (R^2= 0.99198)

Expected Concentration	Number of Values	Mean Calculated Concentration	% Accuracy	Std. Deviation	%CV
0.0078125	1 of 1	1.053e-2	134.7	N/A	N/A
0.0156250	1 of 1	1.441e-2	92.3	N/A	N/A
0.0312500	1 of 1	2.698e-2	86.3	N/A	N/A
0.0625000	1 of 1	5.417e-2	86.7	N/A	N/A
0.1250000	1 of 1	1.139e-1	91.2	N/A	N/A
0.2500000	1 of 1	2.722e-1	108.9	N/A	N/A

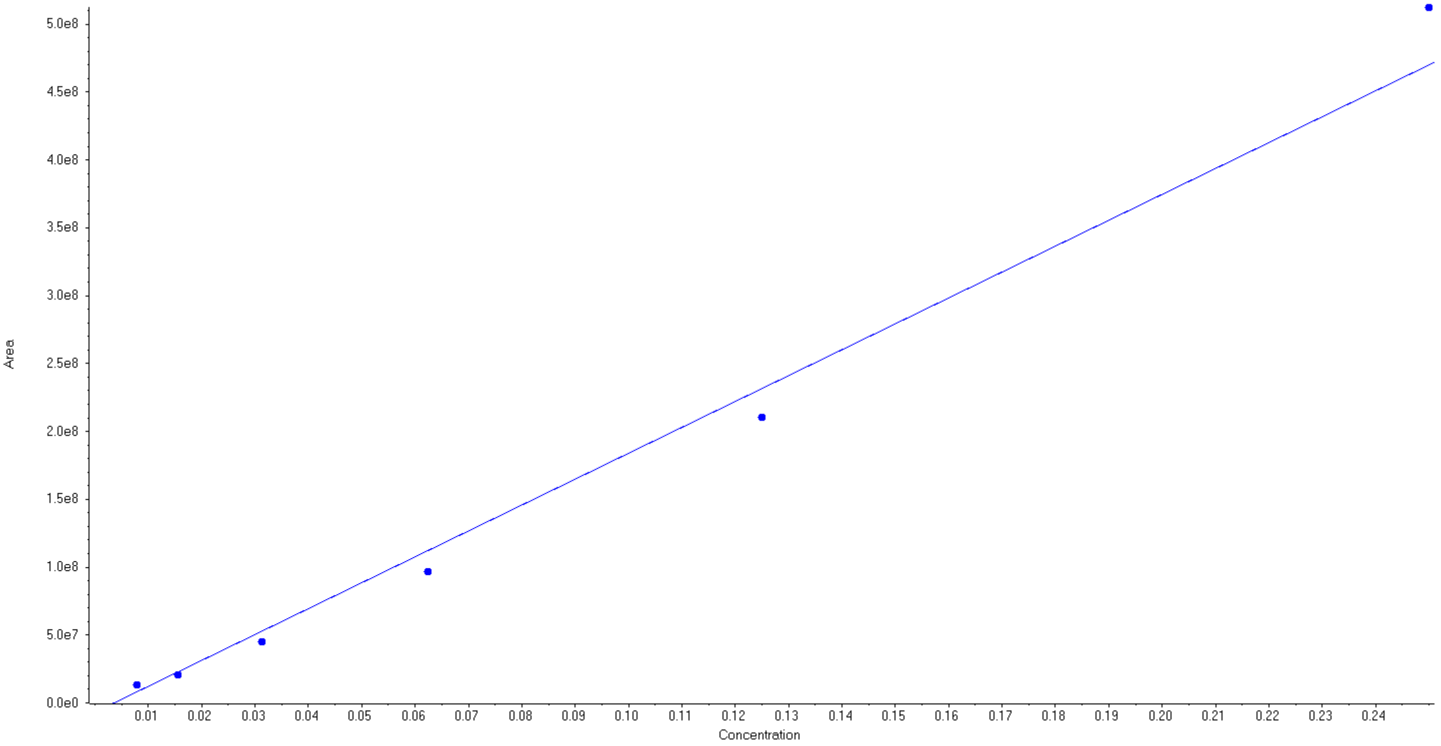

Analyte Name: Val-1
Internal Standard: N/A

**Data File**	Data20231209-YT.wiff	**Result Table**	20231214-YT
**Acquisition Date**	12/10/2023 2:30:36 PM	**Algorithm Used**	MQ4
**Acquisition Method**	20231209-YT.dam	**Instrument Name**	QTRAP 6500+ Low Mass
**Project**	Amino Acid\AA		

Regression Equation: y = 3.39316e9 x + 2.77352e6 (R^2= 0.99869)

Expected Concentration	Number of Values	Mean Calculated Concentration	% Accuracy	Std. Deviation	%CV
0.0078125	1 of 1	7.949e-3	101.8	N/A	N/A
0.0156250	1 of 1	1.508e-2	96.5	N/A	N/A
0.0312500	1 of 1	2.935e-2	93.9	N/A	N/A
0.0625000	1 of 1	6.612e-2	105.8	N/A	N/A
0.1250000	1 of 1	1.314e-1	105.1	N/A	N/A
0.2500000	1 of 1	2.423e-1	96.9	N/A	N/A

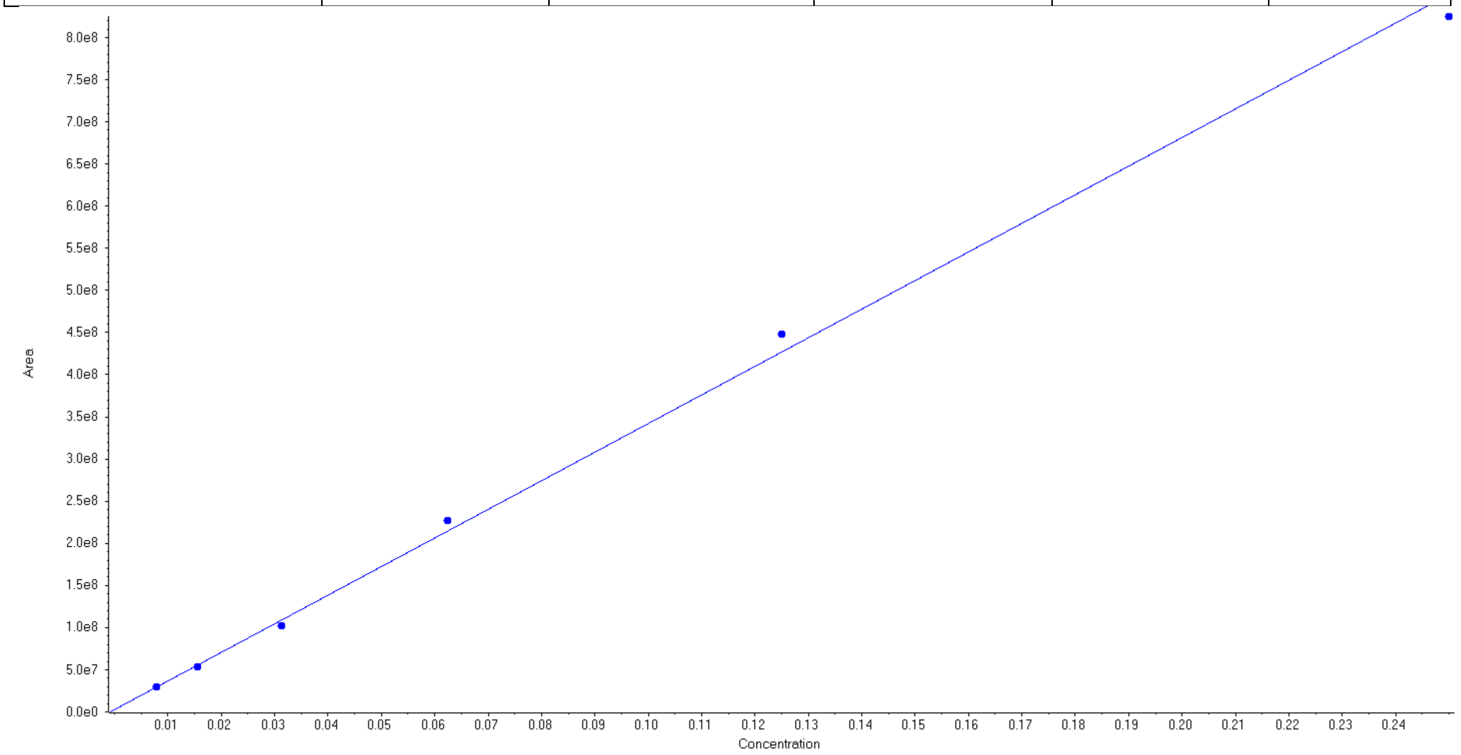

Analyte Name: Pro-1
Internal Standard: N/A

**Data File**	Data20231209-YT.wiff	**Result Table**	20231214-YT
**Acquisition Date**	12/10/2023 2:30:36 PM	**Algorithm Used**	MQ4
**Acquisition Method**	20231209-YT.dam	**Instrument Name**	QTRAP 6500+ Low Mass
**Project**	Amino Acid\AA		

Regression Equation: $y = 8.68471e9\ x + 2.92816e7\ (R^2 = 0.99436)$

Expected Concentration	Number of Values	Mean Calculated Concentration	% Accuracy	Std. Deviation	%CV
0.0078125	1 of 1	6.910e-3	88.4	N/A	N/A
0.0156250	1 of 1	1.455e-2	93.1	N/A	N/A
0.0312500	1 of 1	3.173e-2	101.5	N/A	N/A
0.0625000	1 of 1	7.302e-2	116.8	N/A	N/A
0.1250000	1 of 1	1.343e-1	107.4	N/A	N/A
0.2500000	1 of 1	2.317e-1	92.7	N/A	N/A

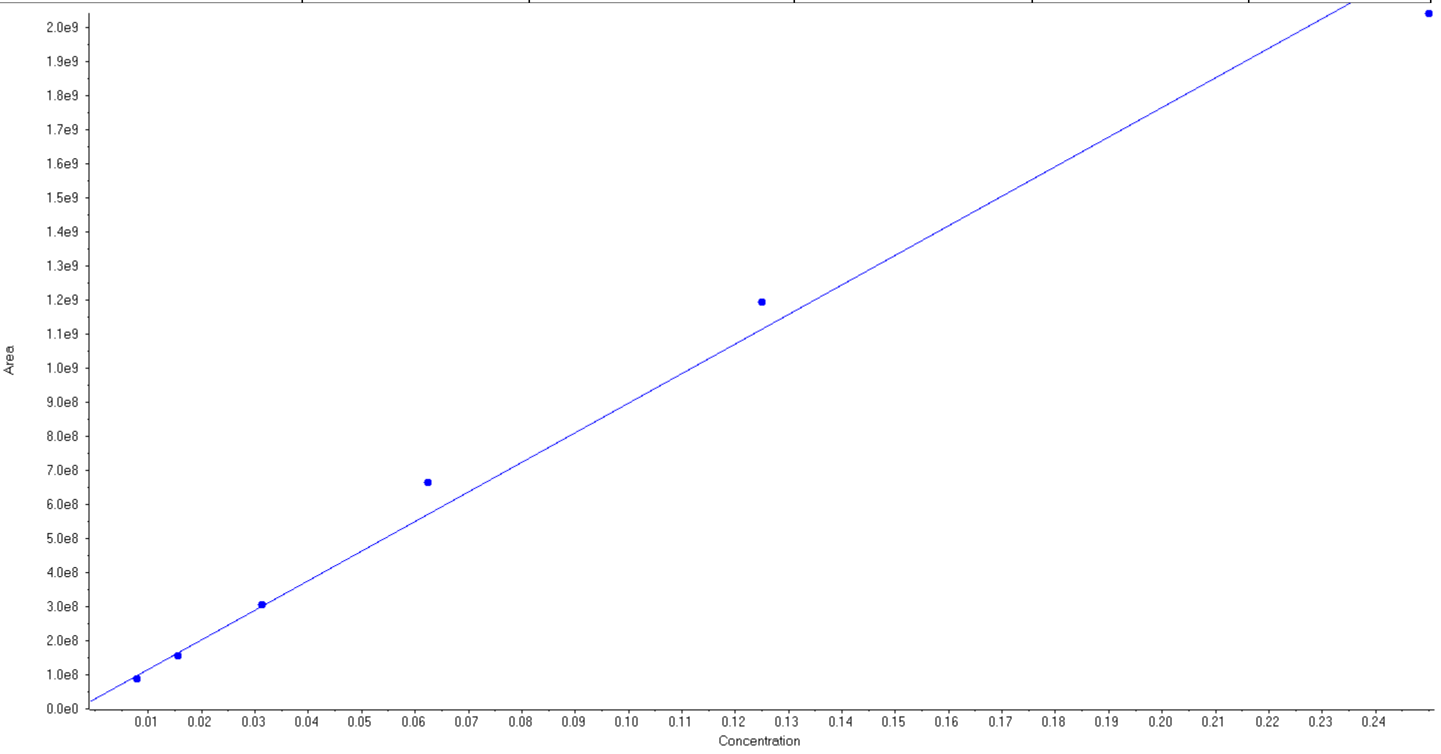

Analyte Name: Ala-1
Internal Standard: N/A

**Data File**	Data20231209-YT.wiff	**Result Table**	20231214-YT
**Acquisition Date**	12/10/2023 2:30:36 PM	**Algorithm Used**	MQ4
**Acquisition Method**	20231209-YT.dam	**Instrument Name**	QTRAP 6500+ Low Mass
**Project**	Amino Acid\AA		

Regression Equation: y = 9.33237e8 x + -2.90630e6 (R^2= 0.99862)

Expected Concentration	Number of Values	Mean Calculated Concentration	% Accuracy	Std. Deviation	%CV
0.0078125	1 of 1	8.854e-3	113.3	N/A	N/A
0.0156250	1 of 1	1.295e-2	82.9	N/A	N/A
0.0312500	1 of 1	3.094e-2	99.0	N/A	N/A
0.0625000	1 of 1	6.407e-2	102.5	N/A	N/A
0.1250000	1 of 1	1.303e-1	104.3	N/A	N/A
0.2500000	1 of 1	2.450e-1	98.0	N/A	N/A

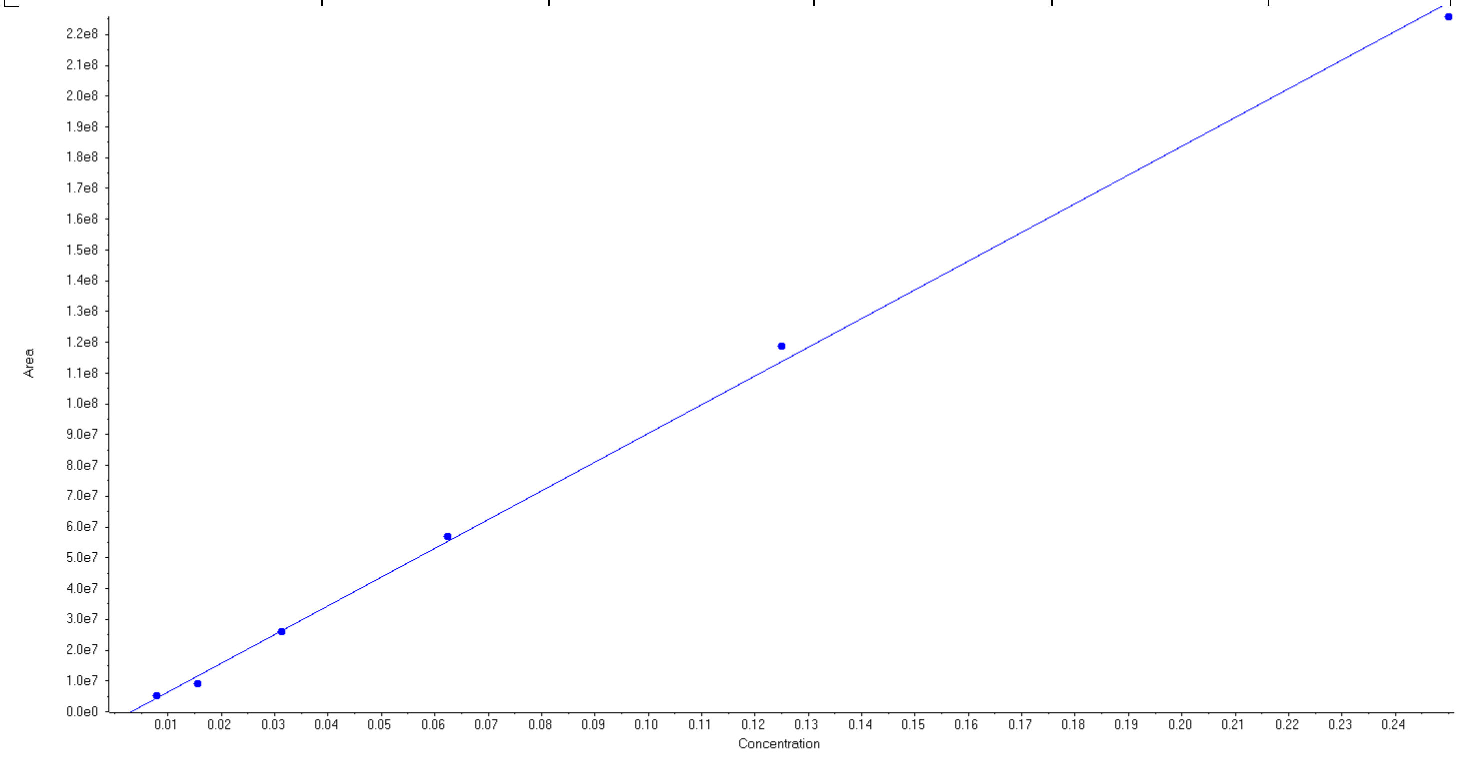

Analyte Name: Glu-1
Internal Standard: N/A

**Data File**	Data20231209-YT.wiff	**Result Table**	20231214-YT
**Acquisition Date**	12/10/2023 2:30:36 PM	**Algorithm Used**	MQ4
**Acquisition Method**	20231209-YT.dam	**Instrument Name**	QTRAP 6500+ Low Mass
**Project**	Amino Acid\AA		

Regression Equation: y = 3.04729e9 x + -8.03731e6 (R^2= 0.99803)

Expected Concentration	Number of Values	Mean Calculated Concentration	% Accuracy	Std. Deviation	%CV
0.0078125	1 of 1	9.609e-3	123.0	N/A	N/A
0.0156250	1 of 1	1.390e-2	89.0	N/A	N/A
0.0312500	1 of 1	2.699e-2	86.4	N/A	N/A
0.0625000	1 of 1	6.100e-2	97.6	N/A	N/A
0.1250000	1 of 1	1.294e-1	103.5	N/A	N/A
0.2500000	1 of 1	2.513e-1	100.5	N/A	N/A

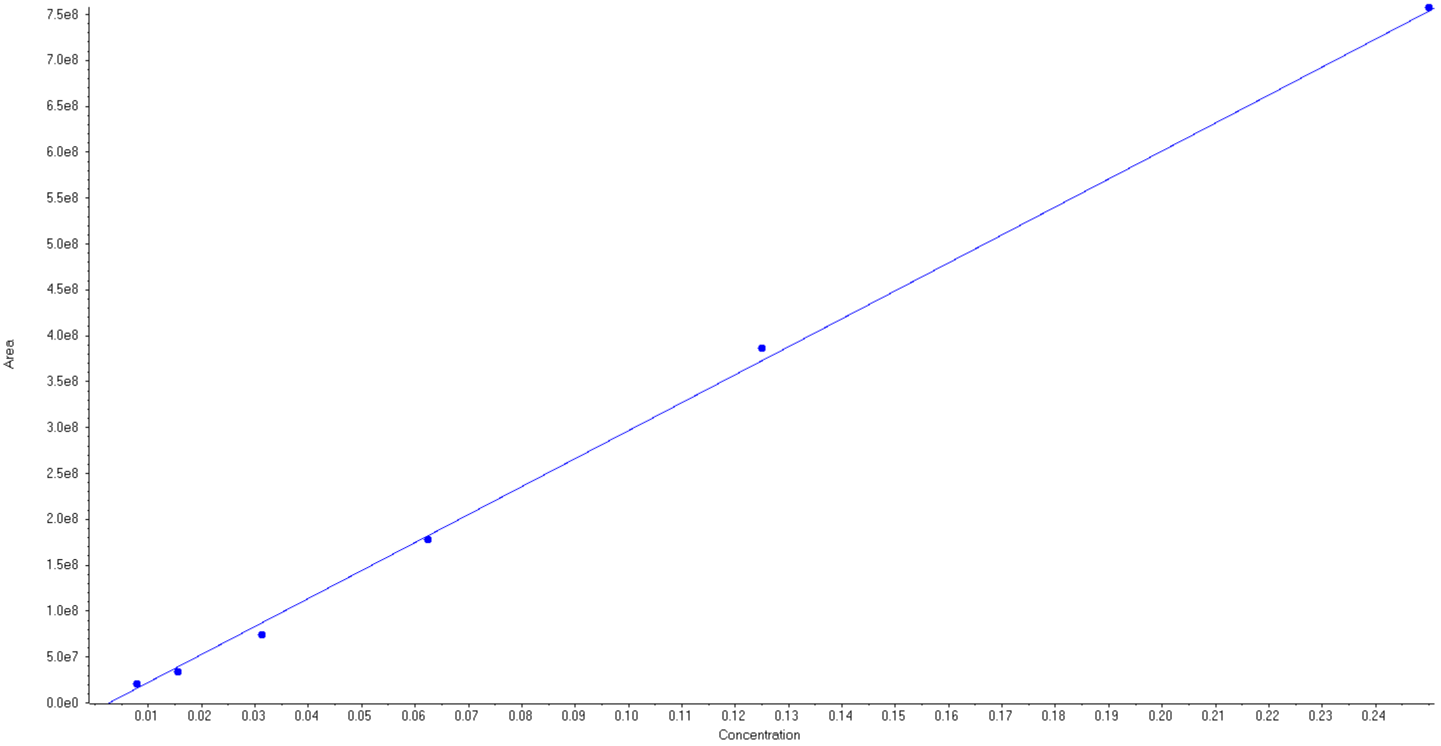

Analyte Name: Leu-1
Internal Standard: N/A

**Data File**	Data20231209-YT.wiff	**Result Table**	20231214-YT
**Acquisition Date**	12/10/2023 2:30:36 PM	**Algorithm Used**	MQ4
**Acquisition Method**	20231209-YT.dam	**Instrument Name**	QTRAP 6500+ Low Mass
**Project**	Amino Acid\AA		

Regression Equation: y = 5.35570e9 x + 1.24887e8 (R^2= 0.99130)

Expected Concentration	Number of Values	Mean Calculated Concentration	% Accuracy	Std. Deviation	%CV
0.0078125	0 of 1	N/A	N/A	N/A	N/A
0.0156250	0 of 1	N/A	N/A	N/A	N/A
0.0312500	1 of 1	2.664e-2	85.2	N/A	N/A
0.0625000	1 of 1	6.894e-2	110.3	N/A	N/A
0.1250000	1 of 1	1.380e-1	110.4	N/A	N/A
0.2500000	1 of 1	2.352e-1	94.1	N/A	N/A

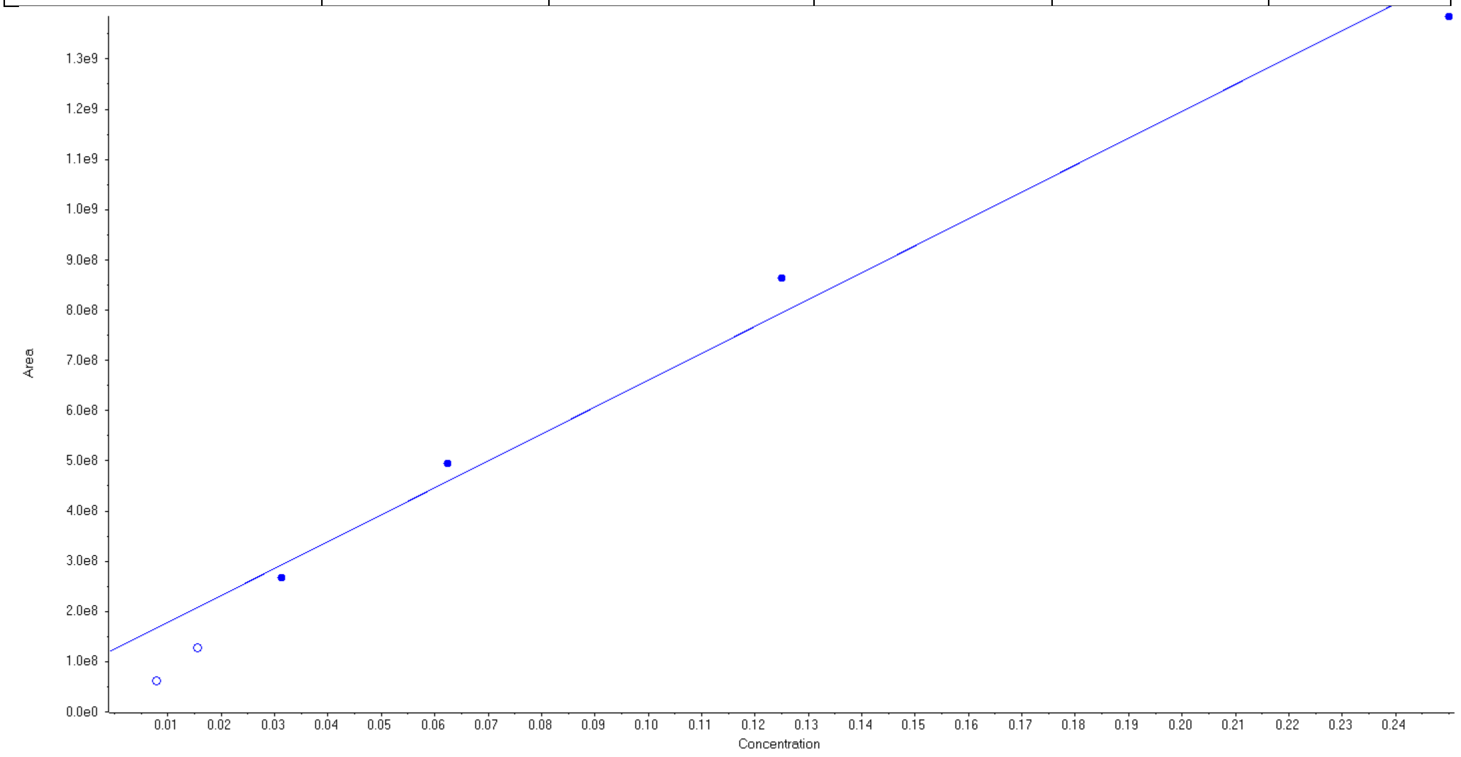

Analyte Name: Gly-1
Internal Standard: N/A

**Data File**	Data20231209-YT.wiff	**Result Table**	20231214-YT
**Acquisition Date**	12/10/2023 2:30:36 PM	**Algorithm Used**	MQ4
**Acquisition Method**	20231209-YT.dam	**Instrument Name**	QTRAP 6500+ Low Mass
**Project**	Amino Acid\AA		

Regression Equation: y = 2.46247e7 x + 6.37752e4 (R^2= 0.99769)

Expected Concentration	Number of Values	Mean Calculated Concentration	% Accuracy	Std. Deviation	%CV
0.0078125	1 of 1	9.980e-3	127.7	N/A	N/A
0.0156250	1 of 1	1.269e-2	81.2	N/A	N/A
0.0312500	1 of 1	2.910e-2	93.1	N/A	N/A
0.0625000	1 of 1	6.067e-2	97.1	N/A	N/A
0.1250000	1 of 1	1.224e-1	97.9	N/A	N/A
0.2500000	1 of 1	2.573e-1	102.9	N/A	N/A

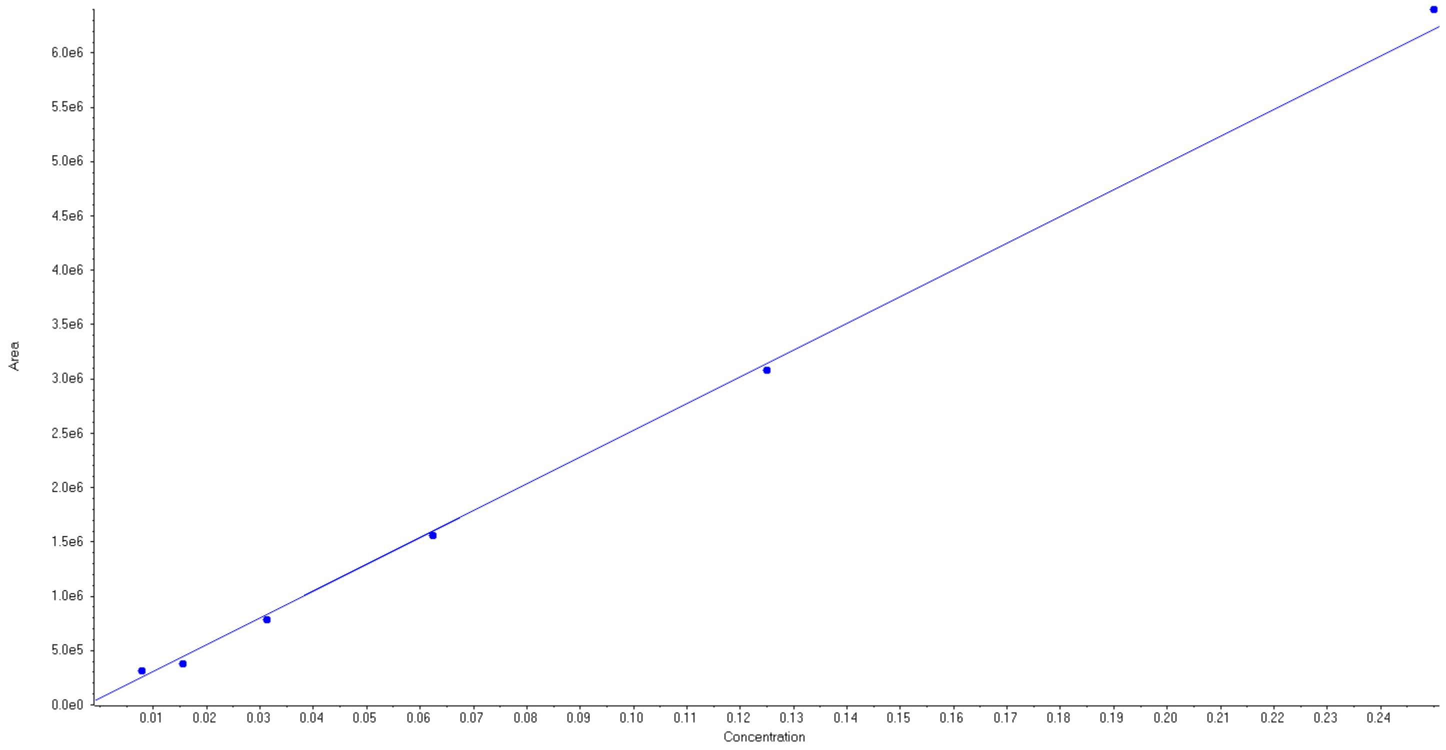

Analyte Name: Arg-1
Internal Standard: N/A

**Data File**	Data20231209-YT.wiff	**Result Table**	20231214-YT
**Acquisition Date**	12/10/2023 2:30:36 PM	**Algorithm Used**	MQ4
**Acquisition Method**	20231209-YT.dam	**Instrument Name**	QTRAP 6500+ Low Mass
**Project**	Amino Acid\AA		

Regression Equation: $y = 6.27988e9\ x + 5.92508e7\ (R^2 = 0.99471)$

Expected Concentration	Number of Values	Mean Calculated Concentration	% Accuracy	Std. Deviation	%CV
0.0078125	0 of 1	N/A	N/A	N/A	N/A
0.0156250	1 of 1	1.286e-2	82.3	N/A	N/A
0.0312500	1 of 1	3.546e-2	113.5	N/A	N/A
0.0625000	0 of 1	N/A	N/A	N/A	N/A
0.1250000	1 of 1	1.370e-1	109.6	N/A	N/A
0.2500000	1 of 1	2.366e-1	94.6	N/A	N/A

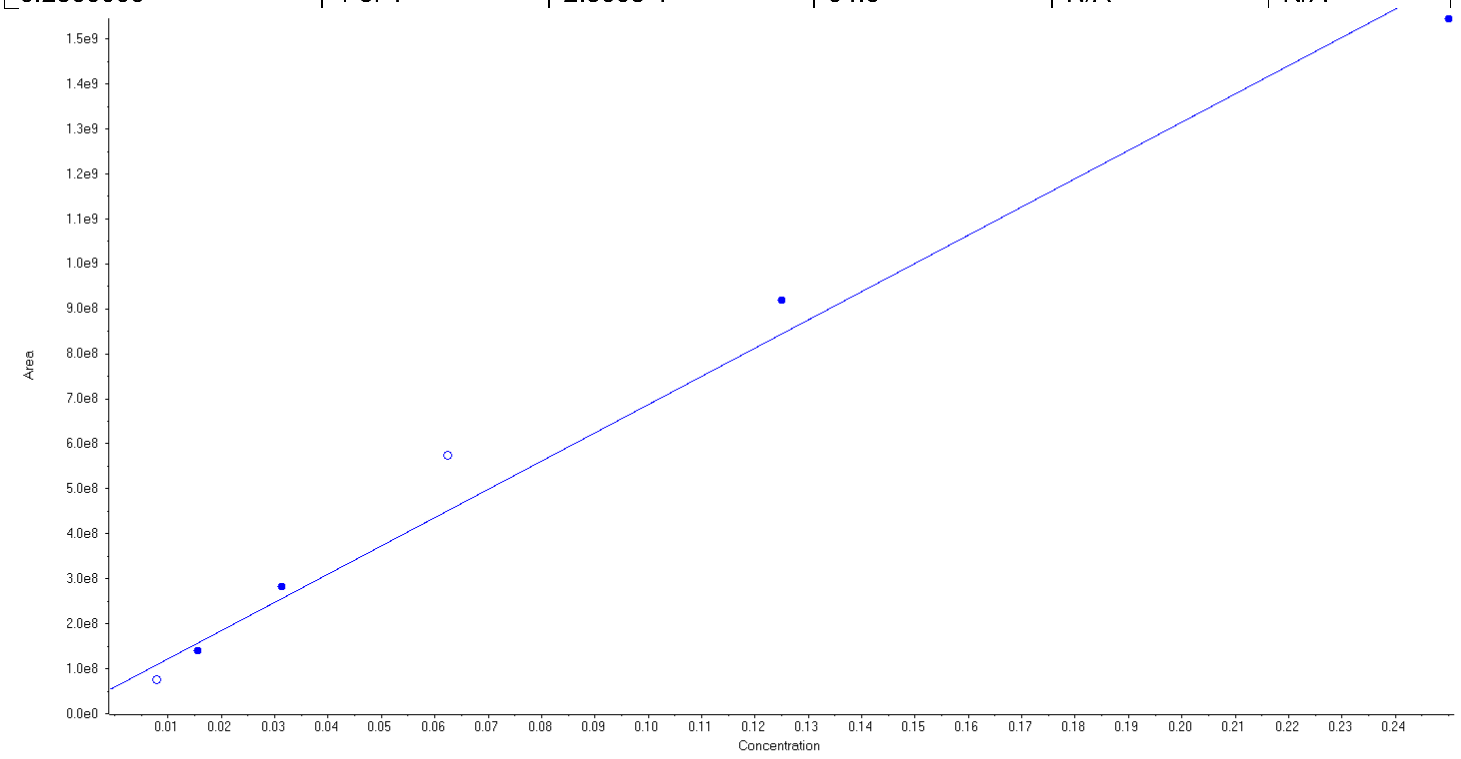

Analyte Name: Trp-1
Internal Standard: N/A

**Data File**	Data20231209-YT.wiff	**Result Table**	20231214-YT
**Acquisition Date**	12/10/2023 2:30:36 PM	**Algorithm Used**	MQ4
**Acquisition Method**	20231209-YT.dam	**Instrument Name**	QTRAP 6500+ Low Mass
**Project**	Amino Acid\AA		

Regression Equation: y = 1.19682e10 x + 4.73940e7 (R^2= 0.99908)

Expected Concentration	Number of Values	Mean Calculated Concentration	% Accuracy	Std. Deviation	%CV
0.0078125	1 of 1	6.795e-3	87.0	N/A	N/A
0.0156250	1 of 1	1.773e-2	113.5	N/A	N/A
0.0312500	0 of 1	N/A	N/A	N/A	N/A
0.0625000	0 of 1	N/A	N/A	N/A	N/A
0.1250000	0 of 1	N/A	N/A	N/A	N/A
0.2500000	1 of 1	2.489e-1	99.6	N/A	N/A

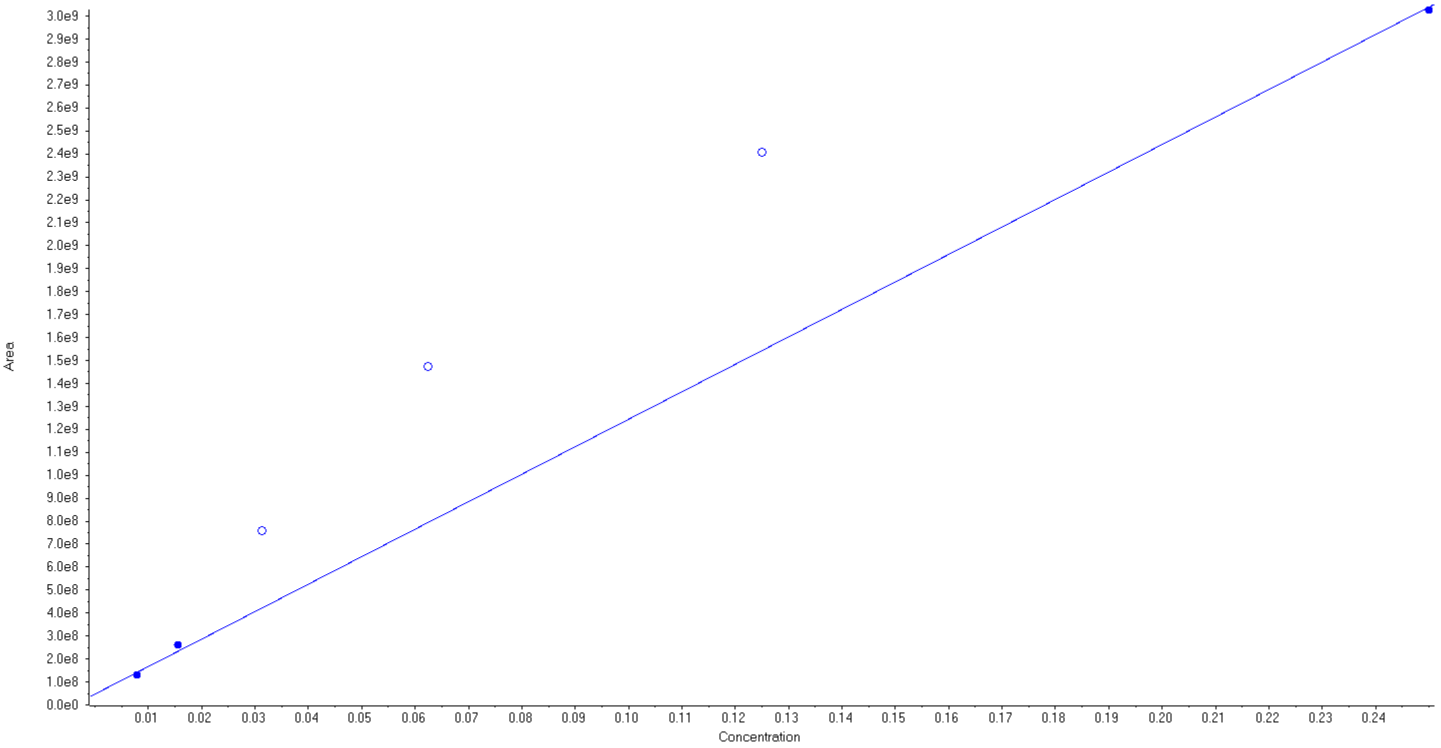

Analyte Name: Tyr-1
Internal Standard: N/A

**Data File**	Data20231209-YT.wiff	**Result Table**	20231214-YT
**Acquisition Date**	12/10/2023 2:30:36 PM	**Algorithm Used**	MQ4
**Acquisition Method**	20231209-YT.dam	**Instrument Name**	QTRAP 6500+ Low Mass
**Project**	Amino Acid\AA		

Regression Equation: y = 7.19353e8 x + -5.98058e5 (R^2 = 0.99906)

Expected Concentration	Number of Values	Mean Calculated Concentration	% Accuracy	Std. Deviation	%CV
0.0078125	1 of 1	8.695e-3	111.3	N/A	N/A
0.0156250	1 of 1	1.547e-2	99.0	N/A	N/A
0.0312500	1 of 1	2.937e-2	94.0	N/A	N/A
0.0625000	1 of 1	5.967e-2	95.5	N/A	N/A
0.1250000	1 of 1	1.216e-1	97.3	N/A	N/A
0.2500000	1 of 1	2.574e-1	103.0	N/A	N/A

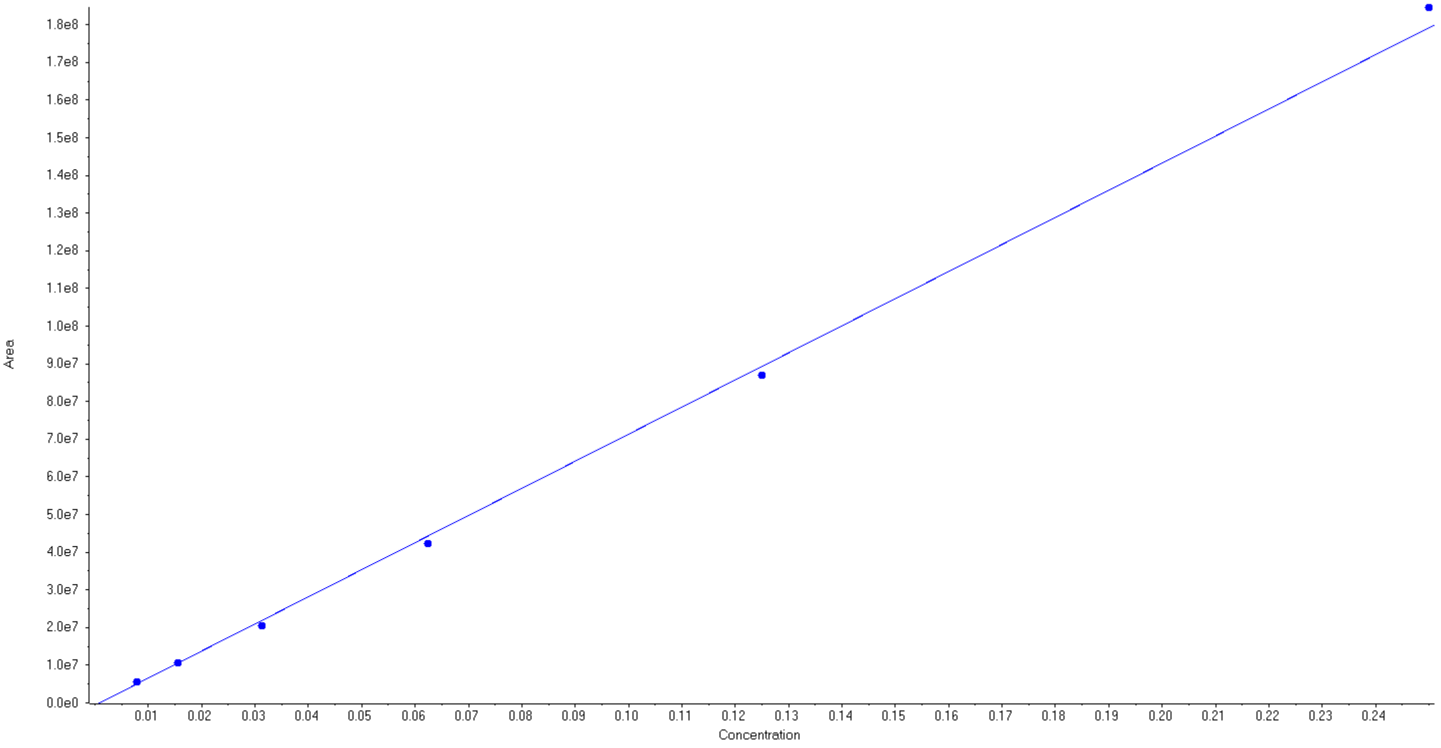

Analyte Name: Thr-1
Internal Standard: N/A

Data File	Data20231209-YT.wiff	Result Table	20231214-YT
Acquisition Date	12/10/2023 2:30:36 PM	Algorithm Used	MQ4
Acquisition Method	20231209-YT.dam	Instrument Name	QTRAP 6500+ Low Mass
Project	Amino Acid\AA		

Regression Equation: y = 5.94334e8 x + -2.25586e6 (R^2 = 0.99001)

Expected Concentration	Number of Values	Mean Calculated Concentration	% Accuracy	Std. Deviation	%CV
0.0078125	1 of 1	1.047e-2	134.0	N/A	N/A
0.0156250	1 of 1	1.494e-2	95.6	N/A	N/A
0.0312500	1 of 1	2.674e-2	85.6	N/A	N/A
0.0625000	1 of 1	5.380e-2	86.1	N/A	N/A
0.1250000	1 of 1	1.106e-1	88.5	N/A	N/A
0.2500000	1 of 1	2.756e-1	110.3	N/A	N/A

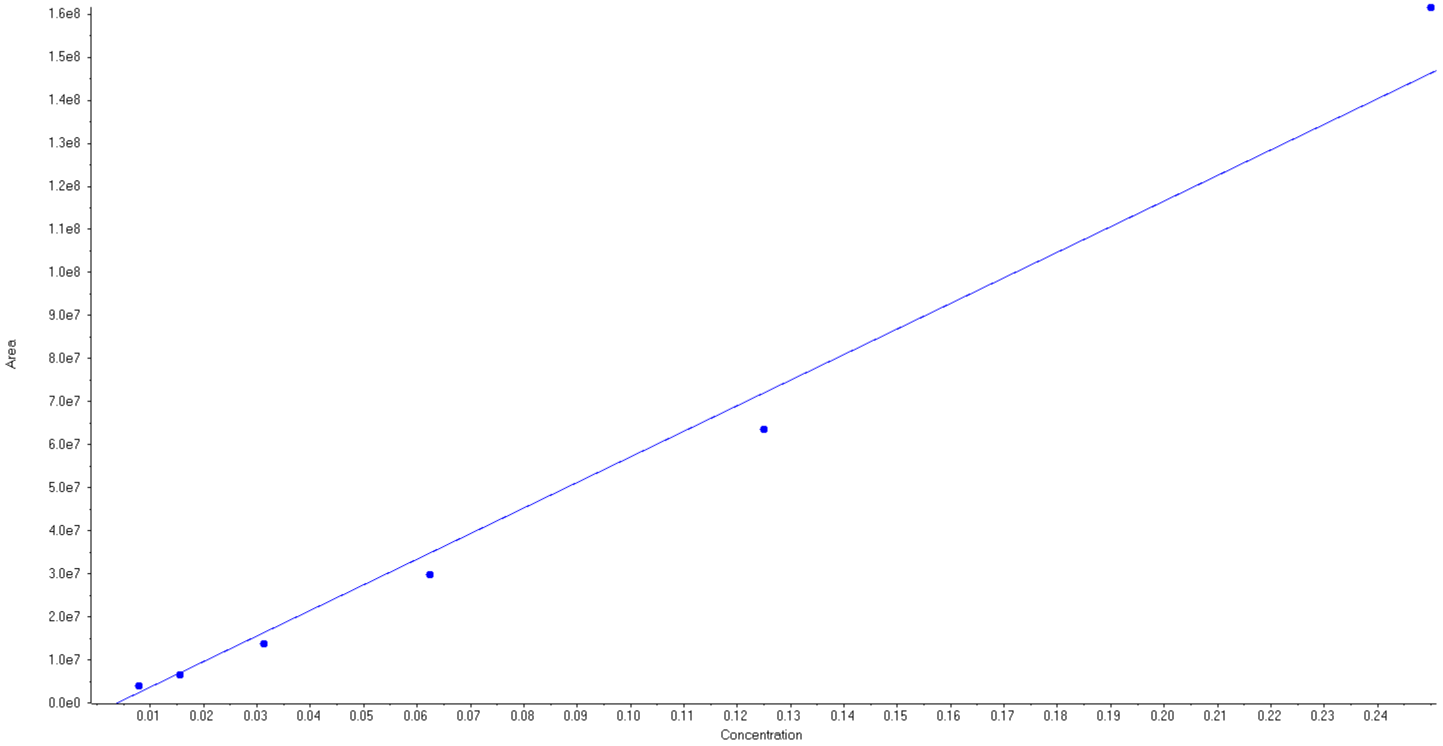

Analyte Name: His-1
Internal Standard: N/A

**Data File**	Data20231209-YT.wiff	**Result Table**	20231214-YT
**Acquisition Date**	12/10/2023 2:30:36 PM	**Algorithm Used**	MQ4
**Acquisition Method**	20231209-YT.dam	**Instrument Name**	QTRAP 6500+ Low Mass
**Project**	Amino Acid\AA		

Regression Equation: y = 8.82977e9 x + 1.29028e7 (R^2 = 0.99452)

Expected Concentration	Number of Values	Mean Calculated Concentration	% Accuracy	Std. Deviation	%CV
0.0078125	1 of 1	7.553e-3	96.7	N/A	N/A
0.0156250	1 of 1	1.434e-2	91.8	N/A	N/A
0.0312500	1 of 1	3.087e-2	98.8	N/A	N/A
0.0625000	1 of 1	6.723e-2	107.6	N/A	N/A
0.1250000	1 of 1	1.408e-1	112.6	N/A	N/A
0.2500000	1 of 1	2.314e-1	92.6	N/A	N/A

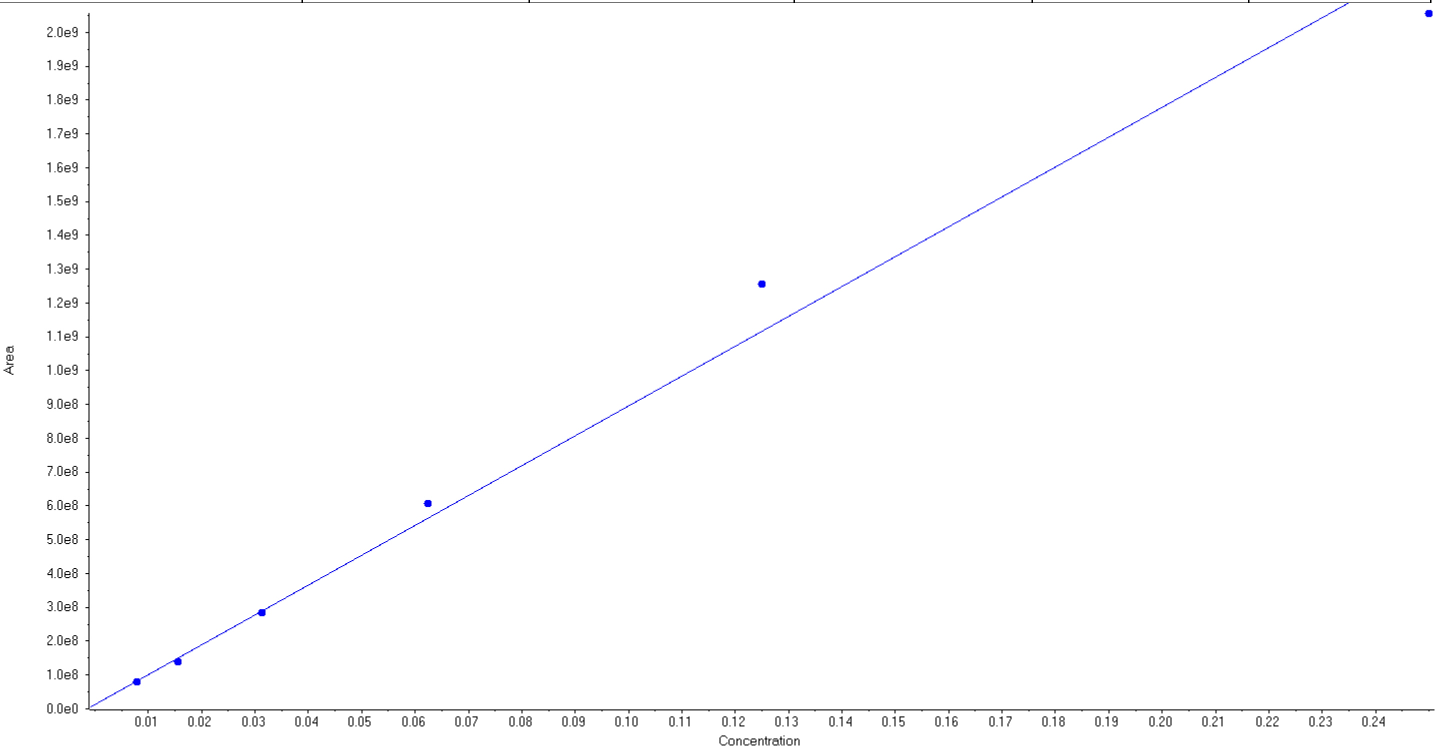

Analyte Name: Phe-1
Internal Standard: N/A

**Data File**	Data20231209-YT.wiff	**Result Table**	20231214-YT
**Acquisition Date**	12/10/2023 2:30:36 PM	**Algorithm Used**	MQ4
**Acquisition Method**	20231209-YT.dam	**Instrument Name**	QTRAP 6500+ Low Mass
**Project**	Amino Acid\AA		

Regression Equation: y = 1.48056e10 x + 3.17538e8 (R^2= 0.99040)

Expected Concentration	Number of Values	Mean Calculated Concentration	% Accuracy	Std. Deviation	%CV
0.0078125	0 of 1	N/A	N/A	N/A	N/A
0.0156250	0 of 1	N/A	N/A	N/A	N/A
0.0312500	1 of 1	2.892e-2	92.5	N/A	N/A
0.0625000	1 of 1	6.949e-2	111.2	N/A	N/A
0.1250000	1 of 1	1.203e-1	96.3	N/A	N/A
0.2500000	0 of 1	N/A	N/A	N/A	N/A

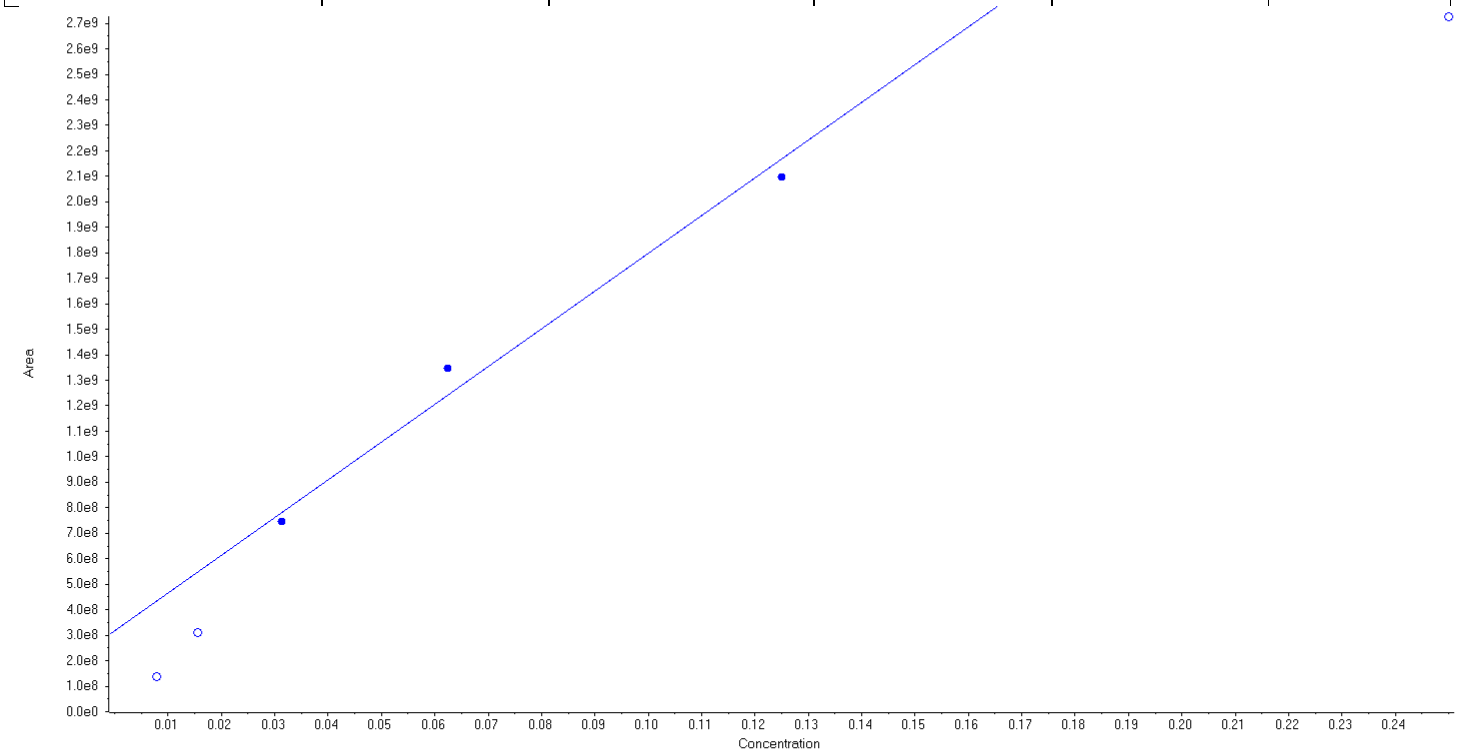

Analyte Name: Asn-1
Internal Standard: N/A

**Data File**	Data20231209-YT.wiff	**Result Table**	20231214-YT
**Acquisition Date**	12/10/2023 2:30:36 PM	**Algorithm Used**	MQ4
**Acquisition Method**	20231209-YT.dam	**Instrument Name**	QTRAP 6500+ Low Mass
**Project**	Amino Acid\AA		

Regression Equation: y = 3.31706e8 x + -1.12606e6 (R^2 = 0.99624)

Expected Concentration	Number of Values	Mean Calculated Concentration	% Accuracy	Std. Deviation	%CV
0.0078125	1 of 1	9.866e-3	126.3	N/A	N/A
0.0156250	1 of 1	1.444e-2	92.4	N/A	N/A
0.0312500	1 of 1	2.777e-2	88.9	N/A	N/A
0.0625000	1 of 1	5.770e-2	92.3	N/A	N/A
0.1250000	1 of 1	1.179e-1	94.3	N/A	N/A
0.2500000	1 of 1	2.645e-1	105.8	N/A	N/A

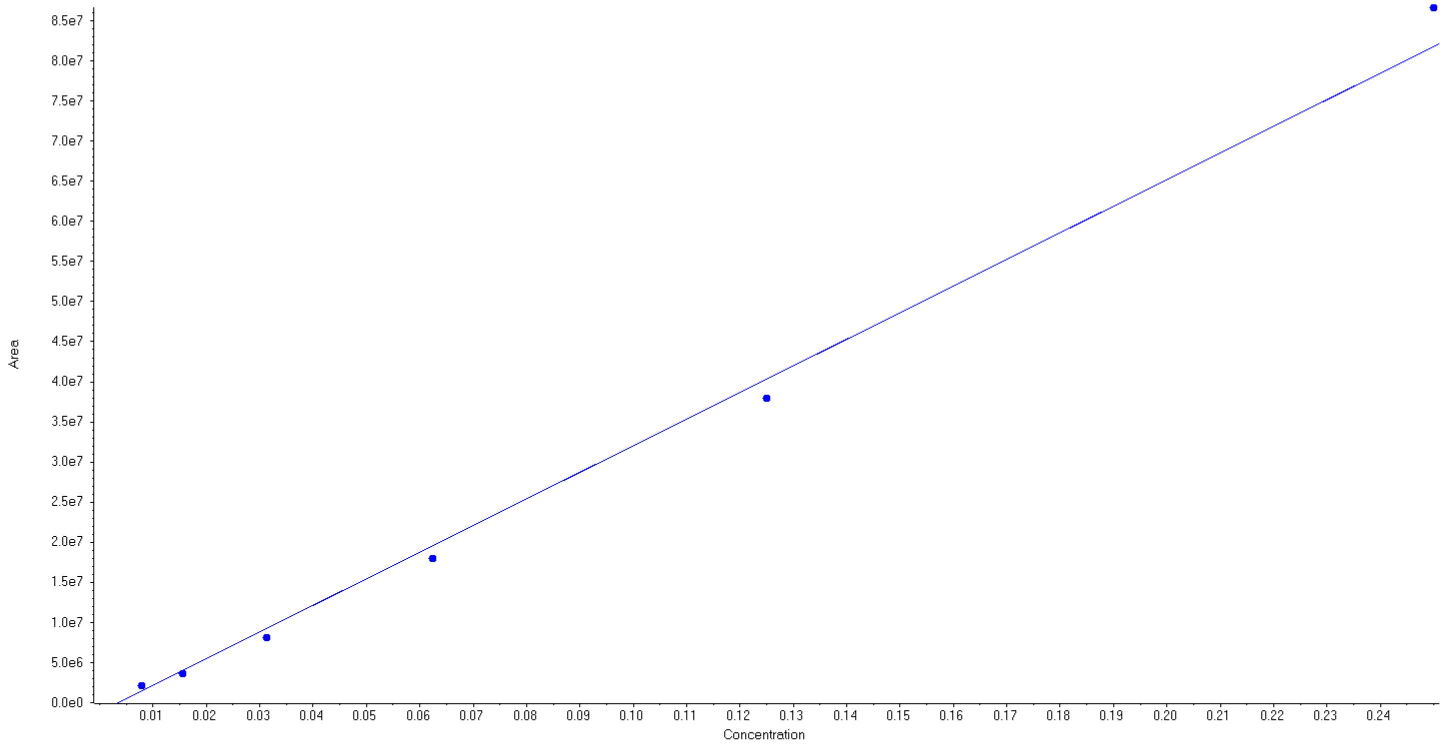

Analyte Name: Cys-1
Internal Standard: N/A

**Data File**	Data20231209-YT.wiff	**Result Table**	20231214-Cys
**Acquisition Date**	12/10/2023 2:30:36 PM	**Algorithm Used**	MQ4
**Acquisition Method**	20231209-YT.dam	**Instrument Name**	QTRAP 6500+ Low Mass
**Project**	Amino Acid\AA		

Regression Equation: y = 2.68638e9 x + -1.09829e7 (R^2 = 0.99741)

Expected Concentration	Number of Values	Mean Calculated Concentration	% Accuracy	Std. Deviation	%CV
0.0078125	1 of 1	9.624e-3	123.2	N/A	N/A
0.0156250	1 of 1	1.499e-2	96.0	N/A	N/A
0.0312500	1 of 1	2.694e-2	86.2	N/A	N/A
0.0625000	1 of 1	5.703e-2	91.2	N/A	N/A
0.1250000	1 of 1	1.250e-1	100.0	N/A	N/A
0.2500000	1 of 1	2.586e-1	103.5	N/A	N/A

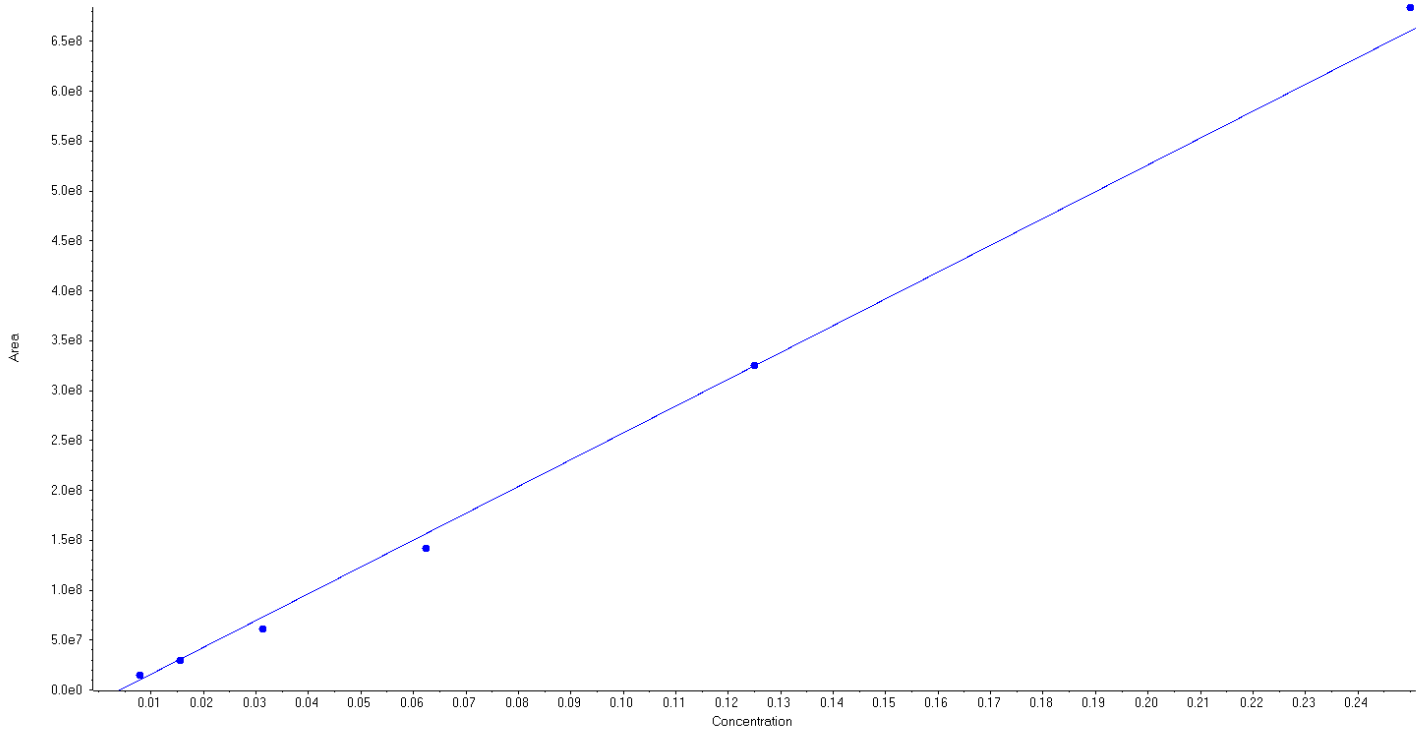

Supplement: Supplementary file 1 [file foods-14-03628-s001.zip › Supplementary file1.pdf]
