# Supplementary material for: Regulatory Mechanisms of Free Umami Amino Acid Accumulation in Fresh Waxy Kernels: Insights from Transcriptome and Metabolomics Analyses
Source: Foods. 2025 Oct 24;14(21):3628. doi: 10.3390/foods14213628 (PMC12609758; doi:10.3390/foods14213628)
Supplement: Supplementary file 1 [file foods-14-03628-s001.zip › Supplementary file2.pdf]

Supplementary Method for Detection of Non-targeted Metabolites

1. Results evaluation

1.1 Sample quality control analysis

The quality control sample (QC) is prepared by mixing the sample extract and used to analyze the repeatability of the sample under the same treatment method. In the process of instrumental analysis, one quality control sample is usually inserted into every 10 test and analysis samples to monitor the repeatability of the analysis process.

1.2 Diagram of total ion flow

The repeatability of metabolite extraction and detection, i.e. technical duplication, can be determined by overlapping the total ion flow diagram (TIC diagram) of mass spectrometry detection and analysis of different QC samples. The high stability of the instrument provides an important guarantee for the repeatability and reliability of the data.

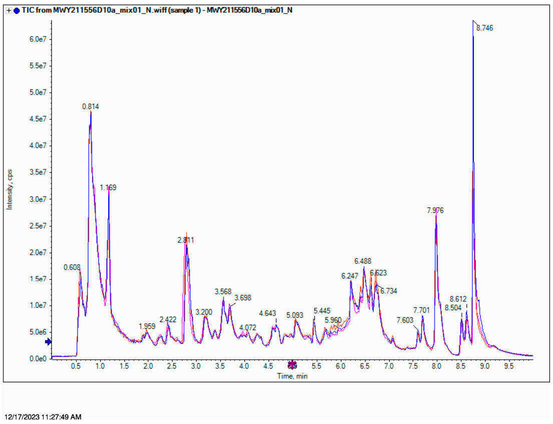

(a)MWY-21-1556D-10-a_neg_QC_TIC

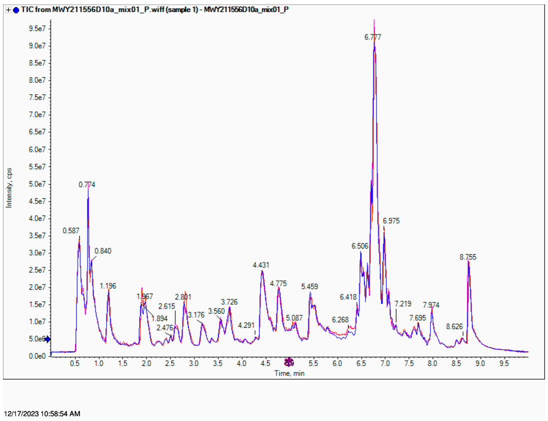

(b)MWY-21-1556D-10-a_pos_QC_TIC

Figure: TIC overlap diagram of QC sample mass spectrometry detection

Note: Overlap diagram of total ion flow diagram (TIC diagram) detected by mass spectrometry of QC samples. The results showed that the total ion current curve of metabolite detection had high overlap, i.e. the retention time and peak intensity were consistent, indicating that the signal stability was good when mass spectrometry was used to detect the same sample at different times. The high stability of the instrument provides an important guarantee for the repeatability and reliability of the data.

1.3 Peak appearance of internal standard in blank samples

The blank sample is interspersed in the whole experimental process, and its

peak situation can reflect whether there is residue in the detection process. It can be seen from the figure below that no obvious peak of internal standard in the blank sample was detected, indicating that there were less substance residues and the cross contamination between samples was within the controllable range.

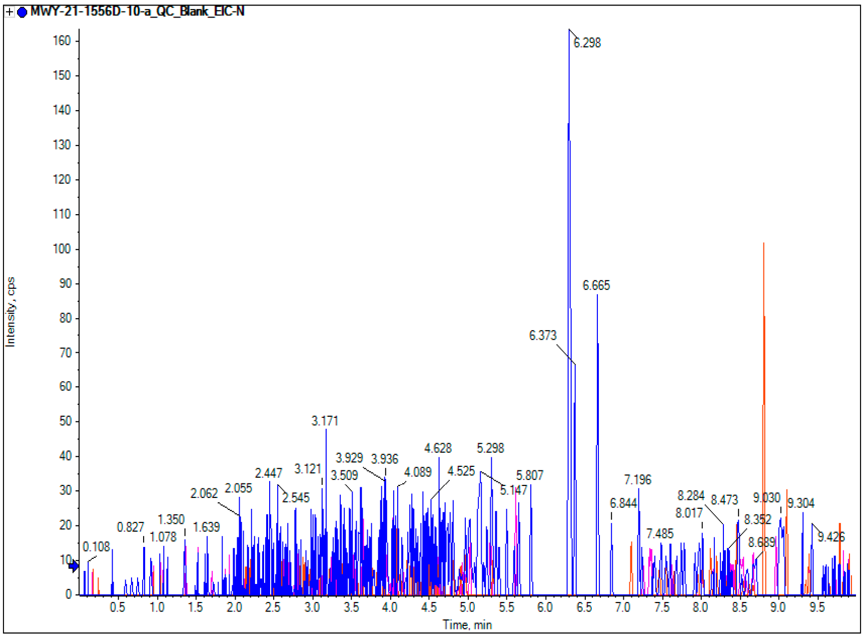

12/17/2023 2:08:16 PM

Figure: EIC diagram of internal standard in blank samples

Note: the signals in the above EIC diagram are noise peaks, and the internal standard substance has no obvious signal peak at the corresponding time.

1.4 QC sample correlation analysis

Pearson correlation analysis was performed on QC samples. The higher the correlation of QC samples (|R| is closer to 1), the better the stability of the whole detection process and the higher the data quality.

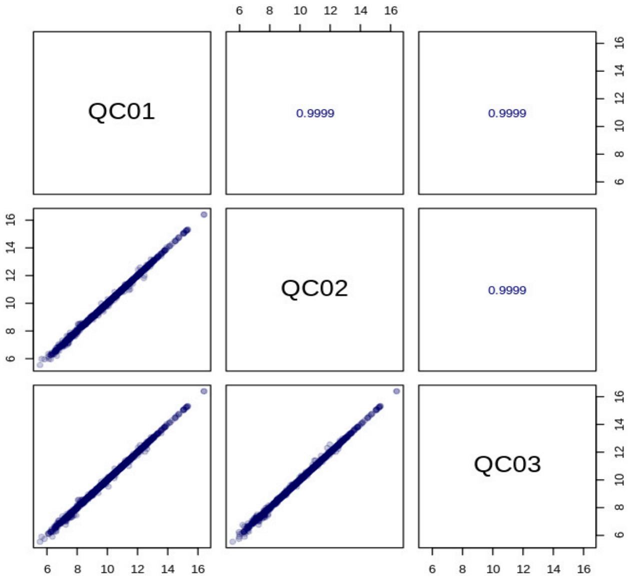

Figure: QC sample correlation chart

Note: the diagonal grid represents the name of QC sample; The lower left corner of the diagonal is the correlation scatter diagram of the corresponding QC sample, and the abscissa and ordinate are the metabolite content (log processing), and each point in the diagram represents a metabolite; The upper right corner of the diagonal is the Pearson correlation coefficient of the corresponding QC sample.

1.5 CV value distribution of all samples

CV value is the coefficient of variation, which is the ratio of the standard deviation of the original data to the average of the original data, and can reflect the degree of data dispersion. The empirical cumulative distribution function (ECDF) can be used to analyze the frequency of the occurrence of CVs of substances smaller than the reference value. The higher the proportion of substances with lower CV values in QC samples, the more stable the experimental data. When the CV value of QC sample is less than 0.3, the proportion of substances is higher than 75%, indicating that the experimental data is very stable.

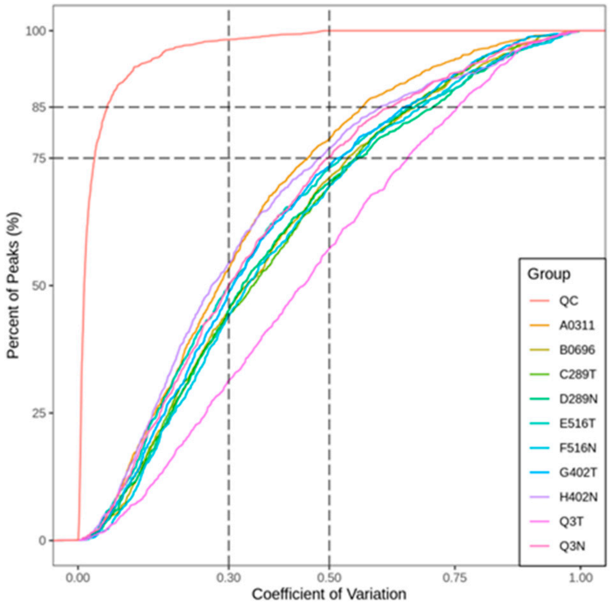

Figure: CV distribution of samples in each group

Note: The names of the detection groups are G402T and H402N, which actually represent maize samples H402-T and H402-N respectively used in this manuscript. Equally, the names of the detection groups are Q3T and Q3N, which represent Q3-T and Q3-N, respectively. The other groups of maize samples are not used in this article. The abscissa represents the CV value, the ordinate represents the proportion of the number of substances less than the corresponding CV value in the total number of substances, different colors represent different grouped samples, QC is the quality control sample, in which the CV values corresponding to the two reference lines perpendicular to the X axis are 0.3 and 0.5, and the number of substances corresponding to the two reference lines parallel to the X axis accounts for 75% and 85% of the total number of substances.
