# Supplementary material for: Regulatory Mechanisms of Free Umami Amino Acid Accumulation in Fresh Waxy Kernels: Insights from Transcriptome and Metabolomics Analyses
Source: Foods. 2025 Oct 24;14(21):3628. doi: 10.3390/foods14213628 (PMC12609758; doi:10.3390/foods14213628)
Supplement: Supplementary file 1 [file foods-14-03628-s001.zip › Supplementary file3.pdf]

>Zm00001eb253820_T001 - Up_Stream_Len 3000bp 1)

LOC542214 glutamine synthetase 4/gln4

tgcatgctctctctctctatctatctctctctctctgcctcactagtcagaaaaaagaatcg
atcttgctcggtgcccccagccgccttctcatggcgtctccccgtcgaacaacgggatttgg
gatttggtgatgagggggtctctgtttttttatttatttcggtgatcgagtggactttatt
tgtttgttctccagttttaaaaaaaaaaatctgctattttggctcgattattcgcccatgaa
tatttggcttcagtttaggatatgcttcaagagacggacagatgcagccctgtgcccctgta
cctcttgtttgtcctgctgctgctgcggcagtctgtggccctgtgctcaccggccaccgcgc
cgcttatatggtgttattactgtaccactggccaccgccagatgacctgttggctaaaagac
ctaatcgcacgtcttacctcttacgcgttaattgacccccccccccccccccccttgtgcta
gtattagtacaccaaagatgcatgatttgttgttagaaacacgtcttggagtgctctgctc
cgaggaatatggtggccagtttgttctgttcagtttgttgttggcgatttgatcctatatta
atgcatgccttttcgggtggttttcaggatcggtggatctggcatggatctcaggagcaaag
caagggtaagataagattcccagcactagtaggaggaccttgggatacttatcacttatata
tctctgaagtcgtcatccgctgagctggtctttggcttatataccagagctctgctcaccac
taccactgcaaacgatttatttttttcttctatttcgtaaaaaataaaaaattcatactgat
ctggtggttgttggtggtgcatggtgctggtgcagaccctctccggcccggtgaccgatccc
agcaagctgcccaagtggaactacgacggctccagcacgggccaggcccccggcgaggacag
cgaggtcatcctgtagtaagtgtcatggcatgccaagcttggatgcgttcttttgtttgttt
tgttttgttttgttttactagcaaacatgggaacgatccttatctcttgggcatgtggcacc
ctgcagcccgcaggccatcttcaaggacccattcaggagggcaacaacatccttgtaagtg
tatacttgtaccatccaagtggcatgaaccatacggttccgtgttatctactgctactgttc
attggtccacctcagatcagtttatctttgtgaagaagggaggaaaaaaaacgtgtcttgtt
tcaccaagtaaactaaaatccctataaatctccctttttaatatgcctgatgcataggctga
agtatcttgatgcagcactagtggttcctgatgcaaatcctttaccctgaacctttgttcag
gtatatgcaataacgcgaattttataacacgatgacattggttgctatgagcttgtatagat
atttttttttgtagttaggtgtgatcacccagtgtaccaaacccaaggtttaggaatccaac
aattggtccttccctctcagcttgtctaacagtagggaccagttttaaggtgctgaacagtg
aactggtttgacccacaggtgatgtgcgattgctacaccccagccggcgagccaatccccac
caacaagaggtacaacgccgccaagatcttcagcagccctgaggtcgccgccgaggagccgt
ggtatgcaaatctctctcccctcccctcccttcccttccccccggtatatgtggcgtaaagc
tgaaacgatctacatctactacccctgactgactgactgaccatgatttgatttgttctttg
caaataaatggcaggtatggtattgagcaggagtacaccctcctccagaaggacaccaactg
gccccttgggtggcccatcggtggcttccccggccctcaggtacacatgacatgatcaccgc
ttgcgcttcacggatgaatttgcttgcttgcttgccgaagacaccaccggttcgatgacaaa
gtacgccgttcgctctccgtgtttttagggtccttactactgtggaatcggcgccgaaaagt
cgttcggccgcgacatcgtggacgcccactacaaggcctgcttgtatgcgggcatcaacatc

agtggcatcaacggggaggtgatgccagggcaggtgagagtgtgagacgctgctgctgacag
cttcttctgtatccctgaatctaccattttctgaccatggcgaaacactggttggttggtt
ggttggttggtttcagtgggagttccaagtcgggccttccgtgggtatttcttcaggcgacc
aggtctgggtcgctcgctacattcttgaggtatggttggttgaccatgtatgtatgtaattt
atgtaacgaaaacgaagcatggacaatgatgcggtaacctgtgctccctttgtttgtttgt
ttgtttttttacagaggatcacggagatcgccggtgtggtggtgacgttcgacccgaagccg
atcccgggcgactggaacggcgccggcgcgcacaccaactacagcacggagtcgatgaggaa
ggagggcgggtacgaggtgatcaaggcggccatcgagaagctgaagctgcggcacagggagc
acatcgcggcctacggcgagggcaacgagcgccggctcaccggcaggcacgagaccgccgac
atcaacacgttcagctgggtacgtacgtgtcttgtccctcccccctggagcctggaatctcgg
ctccgagaacctgcgcccaactgatggatgttatgcgctgcgcttgtggcggtgcagggcgt
ggccaaccgcggcgcgtcggtgcgcgtgggccgggagacggagcagaacggcaagggctact
tcgaggaccgccgcccggcgtccaacatggacccctacgtggtcacctccatgatcgccgag
accaccatcatctggaagccctgaGCGCCGCGGCCGTTGCGTTGCAGGGTCCCCGAAGCGAT
TGCAAAGCCATTGTTCCTTCCGTTCCGTTTGCTTATTATTGTTATTATCTAGCTAGATCATC
TGGGGGTCAGGTCGTCGTGGTGTGCCAAAACAGAACACAGAAAGAGGAAGAAGAAAAAAAAA
ACAAGACGTGTGGCGTTTATGTTATATCATCCAATAATGGCCGTACCACACTTGTGGTACCT
TTCAGTACGTATGCTTGCATTGTATGAGGGGTCGCTTTCCTACGCCCT(5’UTR)

>Zm00001eb190340_T001 - Up_Stream_Len 3000bp

LOC542401 glutamine synthetase 5

acgtgtggaacgtacaagtatacgtattttgttggtttttttttactttttacccggctgg
acgccaaccaactggtttcccgtccttggtcgtcgccattatatagccgccctcctcccggg
ccgatctcttcgtctcaatcccacgccacatcctcccctccttcctccttgggttcccagcc
cgtgcgcccgcctgtcgcagtcgcaccgcagccgccggccatggcctgcctcaccgacctcg
tcaacctcaacctctcggacaccacagagaagatcatcgccgagtacatatggtacggcgct
cgtttccgtcggccgccctctcatggcgtttcccccgtccgggaacggggttgggagagggg
ctctggctctgttttttttttcctgatcaagtggcttcatctgttcattttccgagtaaaaat
ctgttatatttgctcgcttattcgcccatgaatatctaacctgagtatcgggtaattccctg
cccacgggtccctcaccggccaccgcgccgcttatacggtgttgtcgctggccaccgccaga
tgatctgttggctgaaagacctaatcggatgtcttacacgttaattgatcccccccctgatgc
cagtattagttcccaaaagaagcaagtttttttttgttgttaattgttataataaacacgtc
tttggattgctctgctccgaggaatatggcgaggagttgttcggtttgtcgtcgacatgatt
tgatcctatcttaacgcgtgcctttttttggggtgcaggatcggtggatctggcatggatctc
aggagcaaagccagggtaagataacagattcccagcactgggaggacccccccgggatactgg
tggttgtgactcgtcgtcacctatcacttatccctgagctcatccgctgatctgttatttgg
cttatacatacatcagagctctgctcaccactatcactgcaattatttttgtttctctgttc
tacttcgtagtaaaatcatactgatctggttggttctggtgcagaccctcccgggcccggtg

accgatcccagcaagctgcccaagtggaactacgacggctccagcaccggccaggcccccgg
cgaggacagcgaggtcatcctgtagtaagtgtcatggcacgccaggcttggacgcattcctt
tctgtttgctttactgaacgatcctttctcttgggcatgtgctgcagcccgcaggccatctt
caaggacccattcaggaggggcaacaacatccttgtaagtttgcacttctaccatccaagtg
gcatgaaccatacggttccgtgttatttactgctacagtgcctactgttcattggtccaact
cagatcagttcatctttgcgaagaaaaaagaattgtgtcttgttgcaccaagtagactaaaa
gttgtacataccctacaaataaattagatttttttttaaatctgcctgaactatcttgacgc
agcaccagtggttcctgacgcaaattgttttaccctgaacctttgttcaagtatatgcaata
ttgcgaattttataacgcgggtgacattgttgttatgagcgtatatagatattttgtttttt
gtagctaggtgtgatcactcagtgtaccaaaccatggtttaggaatccaattgtctaatagt
agtaggggccgcatttaggtgttgattgaactggtttgacccgcaggtcatgtgcgattgct
acaccccagctggcgagccaattcccaccaacaagaggtacagcgccgccaagatcttcagc
agccttgaggtcgctgccgaggagccctggtacgcgaatctctctcccctccacctccaccg
atatctgtgatgaaaggctgaaacgatctatctctgacgatgcttgtttcttgcaaataaat
gacaggtatggtatcgagcaggagtacaccctccttcagaaggacaccaactggcccctcgg
gtggcctattggcggcttccctggccctcaggtacattcactaatttgcttgcttaaggctt
cttctgtcgaagtttgaagtttcacctaaatgacgtccactgttccgtgcttagggtcctta
ctactgtggaatcggcgcggagaaatcgttcgggcgtgacatagtcgacgcccactacaagg
cctgcctgtacgcaggcatcaacatcagtggcatcaacggggaggtcatgccggggcaggtg
agcagcgcgcggctagacctggcaaaaaccttgttctgtatcagcttccgtctttgttgacc
atggcgttgcggctgggttggtttcagtgggagttccaggtcggaccgtccgtcggcatctc
ttcgggcgatcaggtgtgggttgctcgctacattcttgaggtacgacgccacgtctttctct
gggctagaacatttgtaggacgacgccgtctcgtcttgtcttgtccaagcatgtgtgatttg
tgcgctgcatttttttttttgtttacagaggatcaccgagatcgccggcgtggtggtgacgtt
cgacccgaagccgatcccgggcgactggaacggcgcgggcgcccacaccaactacagcaccg
agtccatgaggaaggagggcgggtacgaggtgatcaaggcggccatcgagaagctgaagctg
cggcacaaggagcacatcgcggcctacggcgagggcaacgagcgccggctcaccggcaggca
cgagaccgccgacatcaacaccttcagctgggtacgcttctaattgtgcaactgctgtaccg
tggaggatcggcgaaacccgtgtgcctgccaactgttgttgctttgtgccgttgcagggagt
cgccaaccgtggcgcgtcggtgcgcgtgggccgcgagacggagcagaacggcaagggctact
tcgaggaccgccggccggcgtccaacatggacccctacgtggtcacctccatgatcgccgag
accaccatcgtctggaagccctgaGGCACCCCGTGGCCGTGTCGTGTCGGTTTGCTCCGCGT
ACGGCGCTGGCCGTTGCATCGCAGGGCCCAGCGGTTGCGCAACTATTTTCCCTTCCCCGTTC
TGTTTGCTTGTACTACTACTCTACCGCTAGTCCTGCATAGCATTTTAGCTAGAACACAACAA
CAGCCAAAAAAAAGTATTGTTGCTTGCTTCGACGCTTGCCACCACTTCCATTCCATGCCGTC
CGTCCGCTTCCTTCCTGTGTAATCCTCCTCCAATAATAGACGTGCCATGTTGCATCCTCTAT
TCCTCTGCATTGTATAAAAGTGGTGTAATTCTTTTGCTACGCCTCCAATGTCTGTGGCTTTT
AGCTGCTGATGCGATGTCAGATTCTGTCACGGACGATTCGTTTCTTTTCCTTCCGATAATTC

AGCGCCGCCGGTCATCT(5’UTR)

>Zm00001eb156610_T001 + Up_Stream_Len 3000bp

LOC103651348 glutamate synthase 1/gogat2 - glutamate synthase2

gaggcactgttgttatcctcggaaaaacagggcggaactttgctgctggcatgagtggaggc
attgcctatgtttatgatgttgatgggaaattcagtgctcgctgcaacaatgagttagttga
tctatatcatgtggaggaagaggatgacatcatcacattgaaaatgatgattgagcaacatc
gtcgcaacactgagagtgttttggctagagatatactctccgattttgataaccttcttcca
aaatttgtaaaagtatatccaagggactataagagggttctagaaagcatgaaggtggaaaa
ggctgttgctaagcatgcaaaggagcctaagatggcaaatggaatttctgtgacaactaagg
tgaggcacattttcaagtggcaactttgtttcacttttcatcacgaattcttacttagctat
tgtaaactattaattcatcatacctaaacatttctagtttagttatgatttcgtattcattt
aatagagaactagttgcacaaactttttatccatcagaaacagctaataatataatttcagtt
cttggaagctttcattgactacttgtatcaactttcatgtacagaaagtacaacctgatcag
tcagcaagccgaccaacacatgttgccaatgccaaaaagtacaggggctttattacatatga
gcgagagagtatttcatacagagatccaaaagagcgtgttagagattggaaggaagttgcaa
tcgaatctacacctggtccactattaaatacacaatctgctcgttgcatggactgtggcact
cctttctgtcatcaggtgatatttctatttttctgtttattatcatatttattcaaattaa
attatctttttccatcatggttaagaagggcgggcctggtgcagcggtagagcctaccgtct
gtaaccggaaggtcccgggttcgagcctcagcctctgcatattatgtgggtaaggcttgacg
tttaaagataccattccctagaccccgcacagtgcgggaagcctaagatggatacattttct
tgatgctaaattgaagttgttgtttccctacagtacttctttgaactgtgatgcaacttgtt
tgttccttttcttatctgctgatgataagattttggaactctttttcaacatttttgtttt
actttcacaggaaagctcaggtgctggttgtcctcttggaaataagatcccagaattcaatg
aattagttcaccagaacagatggcgcgaagcattggatcgactacttgagacaaacaacttc
cctgaattcactggacgagtttgccctgctccttgtgaggggtcatgtgttcttgggattat
tgagaatccggtgtctatcaaaagtatagaatgtgcaatcatagacaaaggttttgaagagg
gatggatgattccacgacctccacttcaaagaacagggtatgtactttaaagtttttaaact
attcatttatttttatccgtgtttgcattttaatttcattactaggtagtacctcggttatg
ctcattaacaatgaaatgatcttattacagaaggaaggtggccattgttggcagtggaccat
ctggtttggctgctgcagatcaactaaataaaatgggccacttcgtaactgtgttcgaacgt
gcagatcgaattggaggtttaatgatgtatggtgtgccaaacatgaagacagacaagattgg
cgttgtccagcgccgtgttaatttaatggctgaagagggtgtcacatttgtggtgaatgcca
atgttggtagtgatcctttgtactcgattgaacgtctacgttctgaaaatgatgcggttatt
ttggcttgtggagcaacaaaaccgaggtaacagactaaaggcaccagataaattttggttga
tactacctgtgtatttgtttgttaagacacagatggtatatcatttgtaactacaaaagaca
cgatttaacatatacttcaaatagttgtgctgtattttgacctatggggatgtctttaacag
agatctcaccatccctggacgcgagctatctggagttcattttgcaatggaatttctccatg

caaatacgaaaagtttactcgacagcaacctggaggatggtaaatatatctccgcccgtggg
aaaaaggtggtggttattggtggaggtgatacaggcacagattgcattggcacatctattag
gcatggttgcaccagccttgtaaatcttgaactcctgtcaaaaccaccaagcactagagctg
ctgacaacccatggccccaggtatgtgtctccctttgcagttatcacacttttttagagtact
gcatttatccttatttccagcttggccttttgcttttagtggccaagaattttccgtgttga
ctatgggcatcaagaagctgctaccaagtttgggaaggatccaagaacttacggagtcttga
caaagcgttttattggcgatggaaatggcaaggtgaaggcccttgaggtagtgcgtgtgaag
tgggagaaagtagatggaagatttcaactcaaggagatggaaggatcagaagagattattga
ggctgatctcgtcctcctagctatgggattcttgggtcctgaagcggttagtacccctcaag
gctattctagttcctaagtacccttgttctagtgcctaaagtagtagagtccgtcttttctt
ccctgcagacaatagctgagaaattgggtttggagaaggacaacagatcaaacttcaaagcg
cagtttggagacttcgctaccagtgtcgatggtgtgtttgccgccggggactgcagacgtgg
acaatcactggtggtttgggccatcacggaggggcggcaggctgctgcagcggtagacaagt
acttgtcaaggtatgatcagaatgctgctgctacaggcgacagcactccctccgggacaggg
ctcgttcagccggttgcggcatagTAGCATAAACTGGAGCAAGTTTTACGTTTGTCAGTGTA
TCCTGTGGTTCCACACAGCAAACACCGTTCTTGTAGTTGTCTAAAAGAAACAGAGAAATCCG
GGCTCATTAAGGAATGAAAGCCCTTCTATATTTTTTTTGACGATAGATAAACAGTTGAAAGT
TTTGGTGGCTATTGTCTCTTTTTTCTGCTGCATGTGGTTTTGTAGTCCAAACAATGTACAGT
TTCCCCTTTCTTAAATAGATGTAAATTTGTCGTGTGATATGTCGGTTATTTTCTATGTGTTT
GCAAGCGCGACTGAGCCACCAGTCTGTATGAGGCCGAGTCTCGGTTTCGGTCGACTTATACC
GTTATACGAATACGTTATACTAGGTGTGTTAGATTTTGGATAGTTGGGC(5’UTR)

>Zm00001eb329710_T001 - Up_Stream_Len 3000bp

LOC542710 ferredoxin-dependent glutamate synthase 1/ZmFd-GOGAT/ferredoxin-dependent glutamate synthase1/fgs1

cactacacacttatcatatttactggatgaatgcactaggcctaataacacttcaattcagt
tctaaatttaatggaatgctggtgcatttcaggccatcatactatgagctcttattcaattg
cttgcaaatggttctctcagagaccctgctgtatggggtgaatccgagattttatgagtact
tatttttgccttgtcattaactgttctattacacagatatcaggtcatgatggtggtactgg
agctagcccaatcagctccatcaaacatgctgggggtccgtgggaacttggtcttacagaaa
caaatcaggtttcctacattttttttgctttgatcgttttctatgaaattattttgtgtt
gtaatgctagttaacttcctccagacacttatacaaaatggattgagagagagggtagtact
tagggtggacggtggattcaggagtggtcaagatgttcttattgctgctgctatgggagctg
atgaatatggctttggttctgtagccatgatagctactggatgtgtcatggcacggatttgt
cacacgaataattgtccagttggagtggctagtcaggtagtgagaacacaaattattttacc
ttcctcttattggactacctcttacattctttgctcttgtagagagaagagcttcgtgcaag
gtttcctggtgttcctggtgatcttgttaactacttcctctttgttgccgaggaggtctgtc
acgattctcattactgcttctgtttggcttagactctgccttgttcctttattgatctgcag

gacctgcctatttttagggataattgtaacttctgaaaattggttaattcatttaggttcg
agctgcattagcccagttgggttatgagaagttggatgacatcattgggcgaacagatttgc
ttaaaccaaagcacatctcattggtgaaaacgcagcacattgatcttggatacctcctatca
gtatgtattggcagccattattatttgaagtgttagcttatcttccatgtgctttctatttt
aatcaatggttcttacatcagaatgctggattgcctgaatggagcagctcccaaattaggag
ccaggacgtccacactaatggccctgttcttgatgagacaatccttgcagatcctgaggttg
tgttttcgataagttctcttagttaaatggactaacttgactaatggctatccactaatcct
gaattttcagtatcatcaatcaaagcaacttatcattccatgttcatgaaactagaactgct
gtatttttggtccatgcatggccttaacttgtctgaaatgaattgcagttgctaggaaccca
tgttttatctgcttgaagcaccattatttacattatctaatttgcagatagctgatgctatt
gagaatgagaaggaagtttccaaggcatttcaaatctataatgttgacagagctgtctgtgg
tcgggtagcaggtgtgattgccaagaagtatggagacacaggttttgctggccagctcaaca
tcacgtatgttgaatctgtcattttacatatgtttatttataacaacagtaggaacttaatc
tttttttggacatttcaggttcaatggaagtgcagggcagtcctttggttgtttcctgactc
ctggaatgaacattcggctagttggggaggccaatgattatgttggaaaggtattaaatgtt
gaacccttgaaacttttctctgggaaactatatgaaatttgaaacttctttttttttttggt
atttttcagggtatggctggtggagaactggtggtagttcctgtagacaaaacaggatttgt
tcctgaggatgctactatagttggaaacacttgtctgtatggagctacaggtggtcaggtat
ttgtgagaggcaaggcaggagaaagatttgctgttagaaactctctatgtcaggcagtggtt
gagggcaccggagatcattgctgtgagtacatgactggtggctgcgtagtcgtacttggcaa
gtaagtagaaccaacagaattctcttttttttcgaaaaaacgcaggaggactgcgttttaga
tatatattaagaagagagagacaaaggtctaagagacccaaaaacacacaacatactcccta
tggaggccagtaacactcgtacatgaaaggatccataaaaaacacccacactctccctacaa
caacctatacaggggcagccaagaaggagagccctttggctccagcaatttctcagtctct
tctttcctcatctgcatgctccataatcagaggaaggctaggagaccctcctccataaaaaa
taactctattacgatgcttccaaatcatccacgactgttggcaattatctccttttcctccc
atccttacacatgcacatttcttaatttgcagagctgggaggaatgttgcagctggaatgac
tggtggtcttgcctacattctagatgaggatgatacacttgtcccaaaggttcacaagctcc
gaattttaagtttacctcgtgtaattcttttgatgtattgtttgccgttgacatgacaccac
atgatgtgattttggttggtgtttttataactttctactttcttaataataggtcaacaagg
aaatcgtcaaaatgcagagagtgaatgctccagctgggcagatgcagctaaagggcttgatt
gaggcctatgttgtaagctgcttttcgttgcaagtatctttttgtagttgtgtattgaatat
cgtgcattcccttacttctgctacttttatgtgtttcaaaaggataaaacgggcagcgagaa
aggtattgcgattttgagggaatgggaggcctatttgccgctcttctggcaactggtaccgc
ccagtgaagaagactcgcctgaggcttgtgcagagtttgagagagtacttgccaagcaagca
accacccaattgtctgcgaagtgaTCGAATCAGACTACAGGAAGGTGGTAGGTGTTTTCTAT
TTGACACAAAGTGATCTCATCTTATCACTTCAAGAAAATCGAAGACCATAACCGGCGCTCTG
AGGCACCGAAGTGCCCGTCTGAAGACTGCAACTTTGTGGAGGCTCTGCCCAGGCCATCTTTT

TGAGTTGAGCTAACAGAACCGAGGGTTCTTCTCGACGCTCGTGCACCTAGGTAACCCCTGTA
AATTGGTTTGCTGACGCTAGGCTGCAAGGGGCCTGCCCTGTTTGAACTGCTGTACTGTACAT
GATTCTTGGCCGTTGCCCTGTGTAATTATGTTTTGGTTAGCTCGTAAATTTATACGCATCCA
ATGGAGGCATACAATGCAAGTCGCAAGCGCAATAGTACGGTGCATGATAAACTGTACGGCCA
GGTTGGATTGGAATGTTTGCTTCGTGAATGAACGAAACCGGCGTCATGCCTGTGGTGTGACC
GCTCTGGTTGGCATCCAATGTTTCTTTTTTGCAGAATCCAATGGTTTCGCAGCAAAAGCTGG
AATCCAATGGTTGGCGCGGCAACATCTATTTCAATATACAAGGTTTAAACAAGAATTCCGTG
ATCTGCTGCTGCGAAACGTAAAGCGTGGGCCAGGGAACAAATGTTCCATTTATTTATTCTGA
AGGGTAGTGCCACGACCACGAGATACATGTGTGAATGTACTTTGTATGCCAATCATTAATCG
ACCATCTAAGACCTTGTTTAGATGTCTACGTATCTACTTCAATTCGATGTATGTATCTACTT
CAATTCGAGGTATGACCGTGTTCGATTGTAGGGGATTTATAATAGCAAAAAAAACTCAATGT
AGGGATAGCAATTTAATCCACAAAT(5’UTR)

>Zm00001eb238900_T002 - Up_Stream_Len 3000bp

LOC100274119 aspartate aminotransferase/glutamate-oxaloacetic transaminase3/AspT/AspAT/got3

tcctgtatgtattaaacttttgatggatttgttactaaatctcatggtcatggcctaccacc
tacaaatttagcttcacttgttaatttggtaacaattcagcctttctaatttagagttattg
tgggcatttatagttctcttgatacccagccaccagccagataaaatttggaaatggaacca
tgagagattattttgtgctcagtgaatctggtctgctgagtgctgacaatttgtctgaaatt
ccttttgtttatgcttttctgtcatctgcagaatgctccagatggttcattcttttttgcttc
atgcatgtgctcataatcccaccggtgtagatcctacggaggaacaatggagagaaatatcc
catcagttcaaggtaatgggaactgaactggggaacaaaaaagttgtatttcctgtgatcct
acaagccacacctaccagatttcactcttgttcctctataggtgaaaaaacattttccattc
tttgacatggcataccaagggtttgccagtggtgatccagagagagatgccaaggcaatccg
aattttccttgaagatggacaccaaattggatgtgctcagtcatacgcaaagaacatgggac
tttatggacaaagagcaggatgcctgaggtattcagttgactagtgccttgctattgtatac
atatagtgttgtcacctgctgtacttcttagtaaattgacatgtgtgatcttttggggcttg
aattaaagaaaaacctgttttacaatttgagtgcaccttactgcattccctctctatctaag
tggatgatattaaatataatgtgctccattgtttgaacttttggtcaaatagtaactatact
gtgaccacttttactttttagataatggtactgcgctcatcactatatgtgtgtttgttacc
aatattgcagtattttgtgtgaggatgagatgcaagcagttgctgtcaagagccaactgcaa
cagatcgcaagaccaatgtacagcaacccacctgttcatggtgcactggttgtttctataat
cctcagtgatccagaattgaagagtttgtggttaaaagaagtcaaggtaaatcgtgaatcct
acataagtacttaatgttcttatatggctaggtggttaaatctattgttagtgaataaagaa
agaatgtggtcaacacgctattgattgtttcagggtatggctgatcgtatcattggaatgcg
gaaggcacttaaggaaaatcttgaaaagctaggttcacctttgtcatgggatcatatcacta
atcaggtaaatacaactgtgagaaatatcggttccatgtttctgaaatctgtatatagttat

tgacatttagaatgacgtgtatattatactagttttcaacattgaccagctggttatcccca
aggctgaggttgcctggtttgtattgtaatgttgttttgagttcagttccgtgcgtggttaa
atcactaacaaatataaagccctgttgagctggccaaccttgggactatcagcctggaatag
gaactgacaaacaagggtatctagtgtttcatttacttaatgctttgaacatagatagtatg
agatacacgatcgttgattttgtagtgacatcttagcagtaggtgtggtaattgtgatgat
atataagtatcttttagaattaagaggaatcatttactgatagtttcatgaagcaaaaaaat
gctcattcatgttggctaccaaaatgttgagttttacattctaggaaacaagtacttcattc
caaattataaagacgatttgcttttctaaataagggcagccctaataccatggtgaaagttg
tttcactgagttatctcttttatagactccactcatgtcggagctccggcactgtgtcttta
ttttttgctttctagatatatttcttttattacgcagctagatacagtgtatatctaaatat
atagtaaaagctatgtattgtaagaagccaaaatgtcttacaatttggaatggatgaagtgc
aatggaaggactgtacccaaacttttgaagttccaaatgaaaagctgacgcatattttaatt
ttttttttctcgaaaaacgcaggagagctgcgcatcattgtattaataagaagaggaaatta
aggtccaagaggaccagtacaaaaaaccacactatggtggccaacaacaagcataaataaaa
ctatccataggactaggatacaaaaaggcgaccaaggaaaaaaattaggtcgggcctgctgc
gcctagagcagccgcaagcccaaggcttccgaggtgtttggcaccagccagcacccataagg
atagctcgtcgatgaagcttctatgcaccttgccaatggttggcaaatctccatcgaaaata
actctgtttctatgcaaccaaaggcaccatgctcccaaaataatggcactgttgactccctt
tctcttgcttctgtgcacctgaatggtcacaagtccccaccaatcagcaaaattagtatctc
atcttgcatattttaattttaagtgtgtatagttactaaccaggacgaatctcatcttgtg
ttgctgaatttactgatacatttgttggatagcaaaagatgtccttgaaacatgtcctttcc
ggtgcggatttacgttttgtgcgtattagtatgtggtgaacttgtatctctgaacaaagggt
acatctctgtacagattggaatgttctgctacagtgggatgacacctgaacaagttgaccgt
ttaacaaatgaataccacatttacatgacccgcaatgggaggataaggtatagcaattactg
atttttttacatcacgatgattcattgaacagtttattagaatgaatctaacttcatccct
tgcccaatttcagcatggctggtgttacgacaggaaatgttggttacctagcaaatgcaatt
catgaggttaccaaaccaaattgaGTTAGGGTCCTACCTTCTTTGGTCGATGGAAGCTGATG
GAATGAGACTGTGAAGCGGCGTTTCCCCCCCTCTGTTCCTGACAGAAATAAGTTTAGCCTGC
TGCTACCATATACATGGACACAGACTCAGATGTAGACACTCAAGTTTTGAGAGCTGTGATCC
TGTTTCATTTAATCAAAAACAATTTTGTTTTTTTTTCATCTTGTCATGGCAAATGTTACACC
AGCTGCAAAATAATTACAATAATTGATCTTGATCCAGCGTCACAGTTACTTCGCAG(5’UTR)

>Zm00001eb152450_T001 + Up_Stream_Len 3000bp

LOC100273311 Aspartate aminotransferase/aspartate aminotransferase/glutamate-oxaloacetate transaminase1/got1

ccacaaccaacctggggaaatcacccaaaagtcttcaccctatctggcttgaacgttaggag
ctaccgctattatgatcctgcaacatgcagccttcacttcgaaggtatggatttcattttca
tgctagatctattagatatgctataataatgtgagtatgctagtcaaattgacaactgtgtt

atatttttttatattcgttaatttttttatggctgtcattgaccaaattttgtgttgctttg
caggactcctggaagacctcggttctgctccttcaggttcaattgtactgctgcatgcctgt
gctcacaaccctactggagtagatcctaccatcgaacagtgggaacagattaggcagctgat
gagatcaaaatcactgcttccgttctttgacagtgcctatcaagtatgcagatgtgcagagc
aaaagtgacttgacattgcaaaaaacacatctggatcctatacactcatcattcacattttt
ttgttaacctgacagggctttgcaagtggaagtcttgacaaagatgctcagtcagtgcgtat
gtttgttgctgatggtggtgaacttctcatggctcagagctacgctaagaacatgggattgt
atggagagcgtgttggcgctttgagcattgtaagataccttcactcttgttcttgttgattt
cacttccgatcatcaggaaatcaaggttgttggcatcttcctctggtgttcagatatattcc
tatgtttaccatccaacttgtgagcttaatgctataggagcgattgatgtgtggaaaattct
gccacttgtgttcaaatttgtattttatctccctacttgttacttgagatatgtcagttcat
atagctcgtatctgtttctttatctgaaatcagaagtgcattgctatggatgggttgagttg
tcaatcacctgtagtcactggtgactacaattttagccttttgccaggattgaattctttcg
catttgtagaatattatattaatgtagaaattgtttttaatgtacagtggacaaccatttac
ttccatcagttccagagatatttgactagttaaggatagtcatgactttatgccttatctgt
taaatgatgatgttatttctcctacattatgcccttttgaacagtcttttaggctgcagagt
gtcctattcatagtaactcttatgtttcaggtatgtaaaagtgccgatgtagctgttagggt
tgaaagtcaactcaaacttgtcatcaggcctatgtattcaaaccctcctcttcatggtgcct
ctatcgttgctaccatactcagggacaggtatcattcttgctattgcaatgccatattcaga
ggtgggtccaagagttatacaaagtggttacttcctgttgattgattctatttattaacttc
cagcgagatgttcaacgaatggactctggaactgaaggccatggctgataggatcattaaca
tgaggcaacaactatttaatgcgctgaaatccagaggtacttgtttctgtcaattgctattt
ttctacaaaatatgattaaatgttgtttgcaaagtaacttacccttttctcaattaccattt
ctttcatggtcttgtattgcaactcttggtgcaaatgtttttcttttgtcctggattaatgt
agctgcttaaggcaagctatgtagctactgctgcatccaagatctcttggagtcttgggtta
tcagcaactattaaaataagagaactttctctttttaccttctcagttgatttatcaagaaa
ttgcatctgaccttaaaatagttatcaatctgtgtgacttatttggggggagcactattaatc
tgtgcttggaatgtttctgtttttatctgaattgaaaggctataatgtcacattctttttg
caccagaaaagaaattaccattatcgaatctcaatgtattgttcctttcttttgtctttcgt
ttcgggtttcgggtcatagcacgatggcactcagtctcgttaataacgtgcaggaacccctg
gtgattggagccatatcattaagcaaattgggatgtttactttcactgggctgaatagcgaa
caagtcgcattcatgaggcaggaataccacatttatatgacatctgatgggtaatttgtcta
ttcaacttttataagtatattgtgtttcctgtatttttgtagtctagttgctacttaacagt
ggctactttggacttcttattaatcttatttgcctttccccccttaagggctagtttgggagt
ccaaaaactggaggggaattgtttggacttcttattaatcttattttagcccctccaatccc
ctccggttttgtggctcccaaactagccgtaagttggggtataggcttaggtacttgtctta
atatgttactccctctgtttttaaatatatgacatttagaaaagctaataggacacgctact
tagttataaaaatgaatcattagcttgtcctacacttcgtagtacttttaagatcatatagc

attgaacaaactggatctgcactatttgacattgatctaaaacgaggtttcattttctcact
accacatggcctttccacatgagttggagtggtttagtgtggaatgtaaactaagggctact
ttgtcaactcaactctattttcccaagggttttccattttctaagggaaaatgaacgaattt
ctcttggggaaatgaaaatcccttgtaaaaatggggtttccaaactagccctaaatttcata
ttaaaccactctaactctaacagtacacacaagaataaggccaacacatgggtctccaaaca
agccctataatggaagtcccaactgtcatagctatattgacaatgtatatattctttttgca
ggaggatcagcatggccggtttgagcatgaggactgtgccccatcttgcagatgccatacac
gctgcagttactcaactgaaatgaGGATAGTATCGCAGCTTTCGTGAATAAAACCTGAATCA
CCCACAACAATGTTCTAAGTACTCAGCCAGTGGTATTTACTGGTTGACCTACTGTAGTTTGC
GTCGGAATAGATATGTTTTTTTACTCTTCGTGGGGCAGTTTTGTACTGGTGGATTCATAAGG
ACTCTGATTATGGTGCGTTCGGAACTTATAATAATAAGCACATGAAATTTTGCTTCAGGTGC
AGTGATGCTGTCTCATTAAGTTATTAGTTATTAGGTTGCTGTCTTATTAAGTTATCAGGTCG
CAGCTGGGTTGAAGCGGAGCACCTGTCTTGATTTATCTCTTCTCTTTTCTAGAGAGCTTCTG
TCATTCGTGTGCCCTTTTATATCATTTTGCTCTATCAATGATTATCCTACAATCTGCAGTAG
TTTTGATTCCGTTTTGTTCTTTGCCTGCTGTACTGGCTCAGAAGTACGGTAATGCTCGCAAG
CTTTGCGGTTGTTGATTTAGGGACTGAAAGGATCTGTTTAGAAAGGTGTCGATTTGGAGACT
CAAAAGGATCAAGACCTTCCAAGGAAGGTGAATTGAGTTTCTCTAAATTTTCTTGTATGAAT
TAAAAAGTTTTGTATCAT(5’UTR)

>Zm00001eb146400_T002 - Up_Stream_Len 3000bp

LOC100276531 glutamate-oxaloacetate transaminase4/got4

catctcagcaaagtgtccatcagatacacgttcgttcgtttttttgccgcgatcggttggttg
cttccgtttcttggatctgaatgagtgaacttaccggggaactgttcgcttccatggatggc
tgcgtccgcagagcgtccgggaggacaaaacacgcggcgaggaccaggatggcgattgtgcg
ggaggaggcagtggacacgtccatcagcccaagggtgagcgcgctgcggccgtccaaaacca
tggccatcaccgatcaggccatggcgctgcggcaggccggcgtgccggttatcggtctagcc
gcgggggagccagacttcgacacgccgcccgcgatcgcggaggtgaggggtgcaattagccc
gcggtataatttcctacagtgagaataacactgaatgcagttgttaaataaaacataatctt
tctagttgaacacttgatttagatattcagcaagaatgtgtcttgttatcaaattgggttat
ttcaggtaaacattcttgttgcttacctagttaccttatccaggccgggatggatgcaatta
ggaatggttatacaagatacactcctaatgctgggactttggagctgaggaaggctatttgt
actaaactccagggtgtgtggatgtatgaagttatgaacggacgtctgttttcgcagagcta
cctttcgatcgatgaaactgaatgacttcgtgtttaattatgactgtacagaggagaacggg
gtatcctacctcccagatgaggtgctggtgagcaatggagctaagcaatgcatcacacaagc
tgtgcttgcagtttgctcacctggtgatgaggtgaccactttttcctgctacaccgtagtgc
atcaaacttctactgctgatgctatattagaatctatatatgtgagcacaagtaccattttt
cctaggtcatgctttgggaagttgtcgaggattatgtttttggtctaattttagaatgaca
gatgtctaaaccgaaccttgtcttgggatttgaaaatgcagcctctgggaattagactatgt

gattgtgcaggtatgtgacttgtggatgatcgtcggtcgacattagggttttgttgatatgg
ctatataattgtaggacaatttttttttgtttcacatcaccagagcaatttatgcactagca
gattaccacattatgcagaataattaatggtcaggatgacagatattggtggcatgtcctca
gatgattattgcttgtagcattagaatattcggtgctgatgtttagcttaacaatgtcaagg
gatccctttgtttcaggttttgattccagccccatattgggtcagttatcctgagatggcta
gactggctgatgcgactccagtgattcttcctacaaacatatcagagaatttcttgttaagg
ccagagttgcttaccgacaagatcaatgagaaatcgaggctcttaattctctgctctccatc
taatccaacagggtcagtatatccaaaggagttgcttgagaaaatagctgatatagtcaaga
agcatccgaggcttttggtaatttctttggtctatatggaacttctaaccaacctttaaatt
tcacttgagcatcatgaaatatcattttatttatgttttgcaggttttatctgatgaaattt
atgagcatattatctatcagcctgcaaaacacacaagctttgcttcactacctggaatgtgg
gaaagaacattaactgtaaatggtttttctaaggtagatatatttgctctttactttgttac
cccctctcctgaaatgcttgtgcactttattagctttatttattgcccttgtcataaatatt
agaatgcaccttttcagaactagatacctaatttattccaaaaccaaaacttggacattaag
taatataactaagttctgaaatatttgatgatggaacttttacaggcttttgcaatgacggg
ttggcgtcttggttaccttgcagcccctaaacattttgttgcagcatgtggaaagatccaaa
gccaggtgaaacgattcatttccagtgtttctttatctctaatatgttaaaggttaagtgat
gtggtactgcacttctagtaaaactaatatttgtaagatcgtctccagcagttcacatatat
ggtcactcaaaacactgttttacactgtagactacattattcatagagtaaagtttgaatat
aaggatgaagatgagtaagctactggagatagcctaagtatgcaaaactatttcttgtgtag
cattgcctgctctaacagtctagcttctcttctgcaaattcagtcaagtattgttgttatcg
tcttgatgccaatgtcccacgtgttttctgcagttcacctcaggtgccagcagcatatcaca
gaaggcaggggttgccgctctgaatcttggctatgctggtggtgaagcagtatcaactatgg
tcaaagcattccaggaacgccgtgactatcttgtgaaaaatttcaaggaactgcctggtgtg
aaaatatcagaacctcaggtaaaagaagatattacgatggttcttctgaaacctagttccac
acttgtataaaatggctaaatgataaagaaaatccaaacctaattatcaatttgcagggtgc
cttttatttgttcattgacttcagcgcctactatgggtctgaagtagaggggtttggcacta
tcaagaattccgagtccctttgcatcttcctattagagaaagcacaggtgtgaaccagcaat
ttatttctatgcattcatctgtgaataatgttgaaataacataactgctatttgtggattgc
actttaggttgcccttgtccctggtgacgcatttggggatgacaagtgcattcgtatttcat
atgctgcatccctaactacacttcagactgcgatgtcaaaattaaaggaagctgttgctctg
ctcaagccctgtgttgctgcgtagCAAGATCACTAAATTGCAATAAGGAGTGACCAAGGCAC
CTTTAACTAACCTTTCACATTATTTTAAACACAATGTTCGAATTGTTGGGCAGACTTGGTCT
GTTGTTTTTATATGTAACATTATGGATTTATGATGTGTACATGCAAATAAAAATGTTTTGTT
TAGAAAAGGCTAGGTTGTGGTACT(5’UTR)

>LOC118476588 small nucleolar RNA U3-2 + Up_Stream_Len 3000bp
AAACCCAGGTAGAGGAGTTATAAGTTATGTGGTTAATATAGGAAATTAAGTATTACCTGCAG
CTTTAGTTGCTTGGATAATTTTCTGTATATTTAATTTCTCAGGTGACATTATTGCTTGATCT

TTTCATATCATGCAAGGTTTGACATTTGTGAATACTATATTAAAATAATCAAATTAACACTA
ACTTGCCTTTCAGTTCACCACTTGCAAATAAAGCAACATCATTTTTTTTGAGAAAATAAAAT
ATAAGTTAGGACTTTCTCTAAACAAATAAAAGGCTCCCTCAGGTCATAGATACTGTCATTTA
GGACAAGATCTGGTGAAACATCAATAACATATTTATTTGGATTAGAAACATGTTACTTATAT
GTTTAGTTTTTTATAATTTTGTTTGAAAATGCTTGCAAAATCTCATGATTTGAAGAATTTTT
GGAATATATTCTAGAAGAAATTAGTGGTCAAAGGTAAACATTGAAACCGTGGAAAGGCAAAT
GTGGCAAACTATTTTTCACCAGAGGGAGTAAATCCTTAGTTTTGATCACTCTAACCATGCAG
TACATATACTATCTCTTCTTCTGCTGATGATTCCAAATGATTTACACAATGTTCTAATTCCC
AAACATACATGCCATGTTCTAATTCCCAAACATACGTGCTATGTTCTAATGAATCTGCAGAG
ACATTTGTACGTGCTGATGGGGCATTCATCCCATTTGCTCATGACTTTGACATCTCCACAGT
CACAACTACAGTTAGGGGTGTTGGTGATATTGGGGATGTTAAGGTTATAGATCTGCAGTGTC
CACTTAATAGATTAATAGGGAGGCGAGTATGCAAAATCGGCAGAAGTTCTGGCCACACGACT
GGAACTGTGATGGCTTATGCCCTTGAGTACAACGACGAGAAAGGAATAAGCTTTTTCACTGA
CCTCCTTGTCGTCGGTGAGAACCGCCAAACATTTGATCTAGAAGGTGACAGTGGAAGCCTCA
TTATCCTGACTGGCCAGGACAGCGAGAAGCCGCGTCCCATCGGGATAATATGGGGTGGCACA
GCAAACCGTGGGAGGCTAAAGCTTAGGTGTGACCATGGCCCTCAAAATTGGACCAGTGGAGT
TGATCTTGGCCGCCTTCTGGATCGTCTTGAACTTGATCTTATCATAACCAGCGAATCACTCA
AAGGTTCGGCATTCTCCTTGCTTTCAGTTATATGTGTTTTACTCGTTTTTGCATCCCTTTTC
AGATACTGGAAAATCCACTGTGATGTACGCTTCTGTTGCTTTTTGTTCCACGACAGATAACT
GACATAATTTATCCTCGATAAAGATGCCGTGCAGCAGCAAAGGCGCGCGTTGGCGGCTGCAG
CTAATTCTGCTGCTGGGGAGTCGTCGACAGCAGCAGCTCCCGTCCTAGAAGAGAAAGTGGAG
GAGATCTTCGAGCCTCTGGGGATCAAGATTGAGCAGCTGCGTCGGCACGACGTTTCGGCCTC
TGAAGCGGAGGAGGCAGCTGGGATCAATGTGGAAGAGCGCCAGTTCATCTCAAACTTTGTGG
GCAGGTCCCCTGTGCGTGATGACCAGGGTGCTCCAAGGCAAATTGCGAACCTGAACAATCCG
TCAGAGGAAGAGCTCGCCATGTTGCTGCACCTAGGCGACCAGTAGCCCAAGCGGCTTCGCAG
ACAGAATCCGACCTCGACGGGAAACGGCGAAGCGCTCATTTCAGTGTCTCTACATCGAAGAG
TCGCAAGTAGTCCGAAGAAGGTCACCTGGAGTTCAGCAAACTGAACCTGCATCAGTAGCTTA
GACCAGCAGCTTCCTGGCACAAACATTATAATGAACTATGCTGAAACTTGATTCAGCACTTA
CATGATTTGGGTGGTATTCCCTTCAGTTTTGCTGTGCTCGTGAAGACTTTAATATGTACAAT
GCTGAGTCCCTGTATCAAAACAATGCAGGTAGTGATAAAATCGTATTGATCACCTTCTCTTT
TCTACCATTATCGGTGGAGTTGTCTCAAGATGACTAGATCAGCTTCTGGCAGGCCTGTCTAG
TGCCCAATGCAACTAGGGCGATGGTACAGGCCCCCAATTTTTTGAGTTTTCTGAAATAGATA
ATATAAGAAGGCAAATATTGTTTGATGTTTTGTAAAATAAGCTGCAAAATCTTGACTTGAAA
ATAGATAGTATGAGAGGATAAGAATATGATAATAGATTAAATATAAAAAGATCCATTTTATA
TATTTTTTTACCGCTATATATAGGTGAGCATACCACCAAAACTAAAAAAAAAACCTCAGAGA
GACAGAGACGGCCCTTGCTTCTGGAAGCCTGATGAGTATTCTTAGAAGTTAGAACGCTGTTT
TTTACGGGTAGGACACATAGCACTTGCTCCCTCCATCCCAAATAGTGAATCTGATGGACAGT
ATTTAGCGTTAATATGTTAGCATCCTAATCATAGTAGCCTTTATTATTTGGGGGTTTGAAGC

AAAGCCCTGTTCAGAAAATTGAGGGAAAGGTTTATTTTTCCTTTTTACAGGCGGACGGGTTT
GATGGGTTTGATGCAGTAGCAAGAGTCGGGGTTGCTGCTGACAATCGGGGAATCGTCTGCAG
CCACAATGTGTTGGCTGCACGCAGGAGAACCTGACTTCTATGACCCGCTCAACGAGACAACG
ACTATGAGGGGCTGTAAATGGGAGGATTTGCAGCAGGATGTATAGAATTTTTTTCCCTGTCA
GCATCACCGGCAACGCAACCATGGTCCATAGGTATCAAGATCCAGCATTGCACTTGGGCCAA
AGGTTGAGCTCTGCGAAGTGTGAACTACAATCATCTAGATGGGCTCGACAGAGGTCAGGCTA
ATGCAGCCTCGATGGGCTCGACCTGAAAGCCCACACATAGGATGCGACCCAGTACACGATTT
GCCGGACACTCCCACCAATTAGTACCACCTCGGTCGCTCGATTGGGTAGTAGCCAGCTTAAA
AGTTCAGCTCTGGAGTCCGGTAGC

>LOC118472467 28S ribosomal RNA - Up_Stream_Len 3000bp

GGATGGCCCGTTGCTCGATGCGTTTTCCGTTTCTCCCGCGCTCGGTGGACCTTCGGTCGCCG
TCCTCGCAAGCAGACCCGCCGCGCCCAGCGCGGAGGGATGCTTTGGATGGCTCGATCGTAGC
GGCTACGCTGGCGCATGAGTTGTCTTGGACCCGTGACTGCTTGGAGGACCCCCGCTGCCGTG
CGGCCGACTCCCGGCGCCCGTGTCCCATCGCTCGTGCGGGCATCCCGTGCCTGCTGCGTTGA
GAAGTGCTTGCGTGCTGCTACCCGTCCCACGGGAAGCCGTGCTCGATACACGTTGCCTTCGT
CGAGCTCACCCCCCGGGGTGCGGCTCGTCGGCTCGAGAGCGCCCGCGGCGTTTGCCTCGTGC
CGCCGTCAGCCTATGGCCGGCGGCACCGAGGACACCTCGCTGGCGCTTTTGGTCTCGGATGT
GGCTCACGCTGAAGGCCGGAGACGCGTTGGCGTCACGCGCCCAAGAATCGGTCCGCCCGAAC
GAACGACGGCCAGCCCGGCACGACGCCTCCGCGCGTAGGCCGGCGCTGGCCCGTCTGCGAGG
ACGTGCTACCTGGTTGATCCTGCCAGTAGTCATATGCTTGTCTCAAAGATTAAGCCATGCAT
GTGCAAGTATGAACTAATTCGAACTGTGAAACTGCGAATGGCTCATTAAATCAGTTATAGTT
TGTTTGATGGTACGTGCTACTCGGATAACCGTAGTAATTCTAGAGCTAATACGTGCAACAAA
CCCCGACTTCCGGGAGGGGCGCATTTATTAGATAAAAGGCTGACGCGGGCTCTGCCCGCCGA
TCCGATGATTCATGATAACTTGACGGATCGCACGGCCTTCGTGCCGGCGACGCATCATTCAA
ATTTCTGCCCTATCAACTTTCGATGGTAGGATAGGGGCCTACCATGGTGGTGACGGGTGACG
GAGAATTAGGGTTCGATTCCGGAGAGGGAGCCTGAGAAACGGCTACCACATCCAAGGAAGGC
AGCAGGCGCGCAAATTACCCAATCCTGACACGGGGAGGTAGTGACAATAAATAACAATACCG
GGCGCGTTAGTGTCTGGTAATTGGAATGAGTACAATCTAAATCCCTTAACGAGGATCCATTG
GAGGGCAAGTCTGGTGCCAGCAGCCGCGGTAATTCCAGCTCCAATAGCGTATATTTAAGTTG
TTGCAGTTAAAAAGCTCGTAGTTGGACCTTGGGCCGGGCCGGCCGGTCCGCCTCACGGCGAG
AACCGACCGGCTCGACCCTTCTGCCGGCGATGCGCTCCTGGCCTTAACTGGCCGGGTCGTGC
CTCCGGCGCCGTTACTTTGAAGAAATTAGAGTGCTCAAAGCAAGCCATCGCTCTGGATACAT
TAGCATGGGATAACATCATAGGATTCCGGTCCTATTGTGTTGGCCTTCGGGATCGGAGTAAT
GATTAATAGGGACAGTCGGGGGCATTCGTATTTCATAGTCAGAGGTGAAATTCTTGGATTTA
TGAAAGACGAACAACTGCGAAAGCATTTGCCAAGGATGTTTTCATTAATCAAGAACGAAAGT
TGGGGGCTCGAAGACGATCAGATACCGTCCTAGTCTCAACCATAAACGATGCCGACCAGGGA

TCAGCGGGTGTTACTAATAGGACCCCGCTGGCACCTTATGAGAAATCAAAGTCTTTGGGTTC
CGGGGGGAGTATGGTCGCAAGGCTGAAACTTAAAGGAATTGACGGAAGGGCACCACCAGGCG
TGGAGCCTGCGGCTTAATTTGACTCAACACGGGGAAACTTACCAGGTCCAGACATAGCAAGG
ATTGACAGACTGAGAGCTCTTTCTTGATTCTATGGGTGGTGGTGCATGGCCGTTCTTAGTTG
GTGGAGCGATTTGTCTGGTTAATTCCGTTAACGAACGAGACCTCAGCCTGCTAACTAGCTAT
GCGGAGCCATCCCTCCGTAGTTAGCTTCTTAGAGGGACTATGGCCGTTTAGGCCACGGAAGT
TTGAGGCAATAACAGGTCTGTGATGCCCTTAGATGTTCTGGGCCGCACGCGCGCTACACTGA
TGTATCCAACGAGTATATAGCCTTGGCCGACAGGCCCGGGTAATCTTGGGAAATTTCATCGT
GATGGGGATAGATCATTGCAATTGTTGGTCTTCAACGAGGAATGCCTAGTAAGCGCGAGTCA
TCAGCTCGCGTTGACTACGTCCCTGCCCTTTGTACACACCGCCCGTCGCTCCTACCGATTGA
ATGGTCCGGTGAAGTGTTCGGATCGCGGCGACGGGGGCGGTTCGCCGCCCCCGACGTCGCGA
GAAGTCCATTGAACCTTATCATTTAGAGGAAGGAGAAGTCGTAACAAGGTTTCCGTAGGTGA
ACCTGCGGAAGGATCATTGCCGTGACCCTTAAACAAAACAGACCGCGAACGAGTCACCCGTG
CCGCCGGGCTCCGGCCCGGCACGCTGCCCCCCCCGAACCTCCCGCGGGGAAGGGGGGTGCC
GCGAAAAAGAACCCACGGCGCCCCGGGCGCCAAGGAACACCAGTACTACCTCCTGCCCCGCG
GAGCGGTCGGCCCGCCTTCCGCTCCCAGGGCAGCGGTTACACCTTAATCGACACGACTCTCG
GCAACGGATATCTCGGCTCTCGCATCGATGAAGAACGTAGCAAAATGCGATACCTGGTGTGA
ATTGCAGAATCCCGCGAACCATCGAGTTTTTGAACGCAAGTTGCGCCCGAAGCCTTCTGGCG
GAGGGCACGTCTGCCTGGGCGTCACGCCAAAAGACACTCCCAACACCCCCCCGCGGGGCGAG
GGACGTGGCGTCTGGCCCCCCGCGCCGCAGGGCGAGGTGGGCCGAAGCAGGGGCTGCCGGCG
AACCGCGCCGGGCGCAGCACGTGGTGGGCGACATCAAGTTGTTCTCGGTGCAGCGTCACGGC
GCGCGGCCGGACATTCGGCCCTAAGGACCCATCGAGCGACCGAGCTTGCCCTCGGACCG
