# Supplementary figures and images for: Regulatory Mechanisms of Free Umami Amino Acid Accumulation in Fresh Waxy Kernels: Insights from Transcriptome and Metabolomics Analyses
Source: Foods. 2025 Oct 24;14(21):3628. doi: 10.3390/foods14213628 (PMC12609758; doi:10.3390/foods14213628)

**A**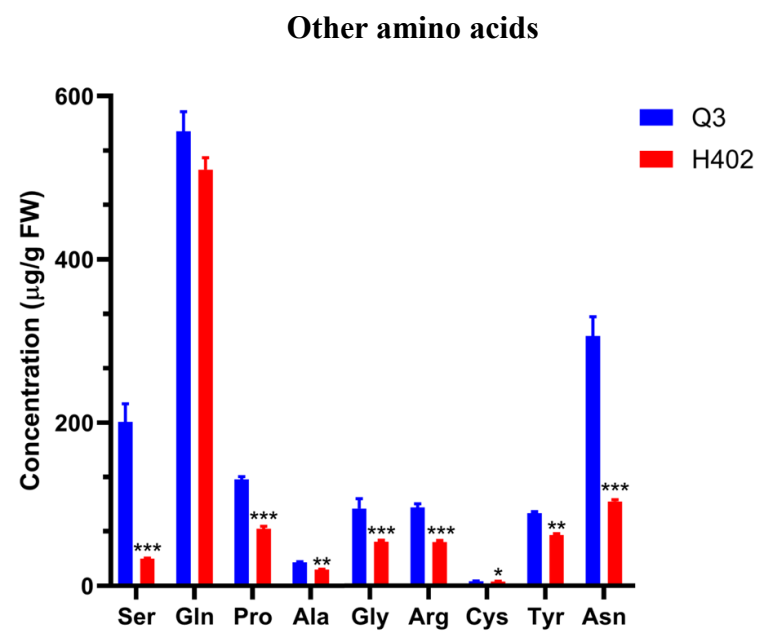**B**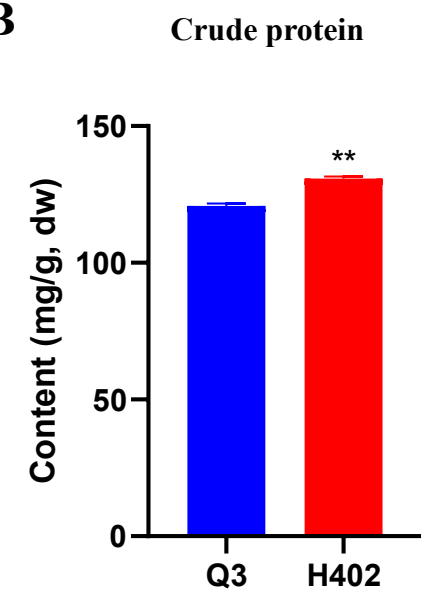**Figure S1**

Supplement: Supplementary file 1 [file foods-14-03628-s001.zip › Supplementary Figure1.pdf]

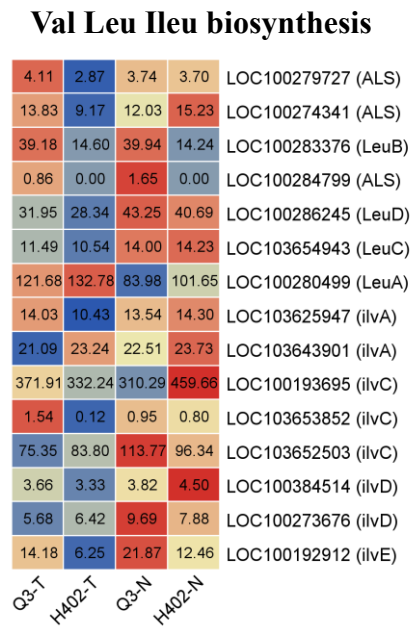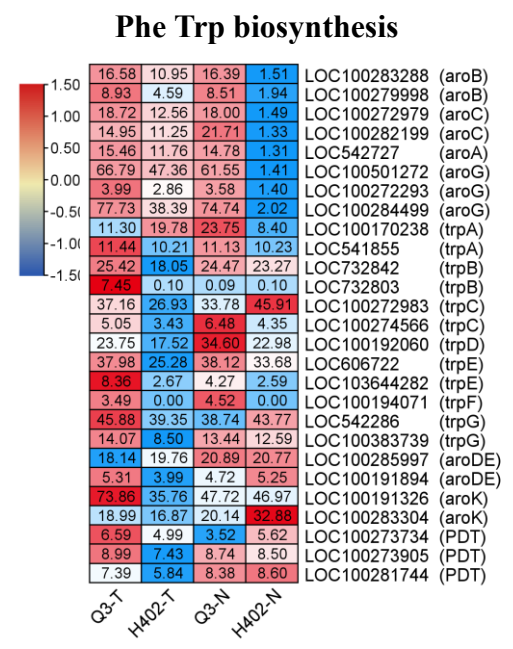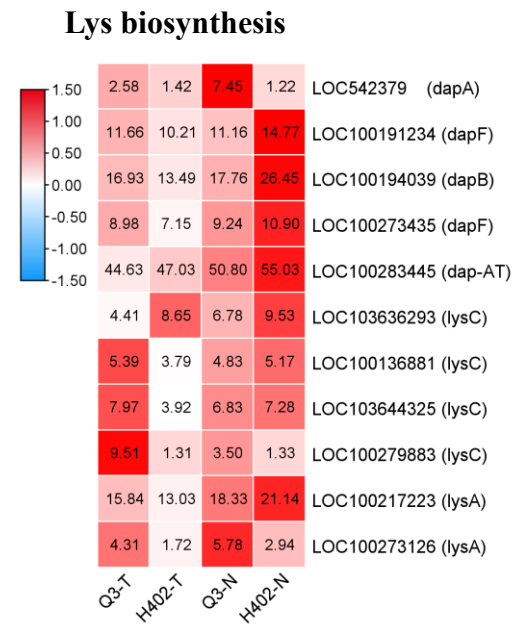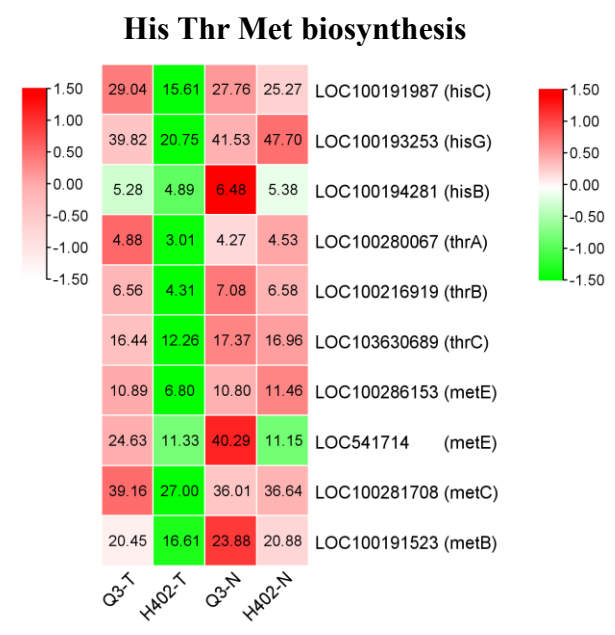

Figure S2

Supplement: Supplementary file 1 [file foods-14-03628-s001.zip › Supplementary Figure2.pdf]

A

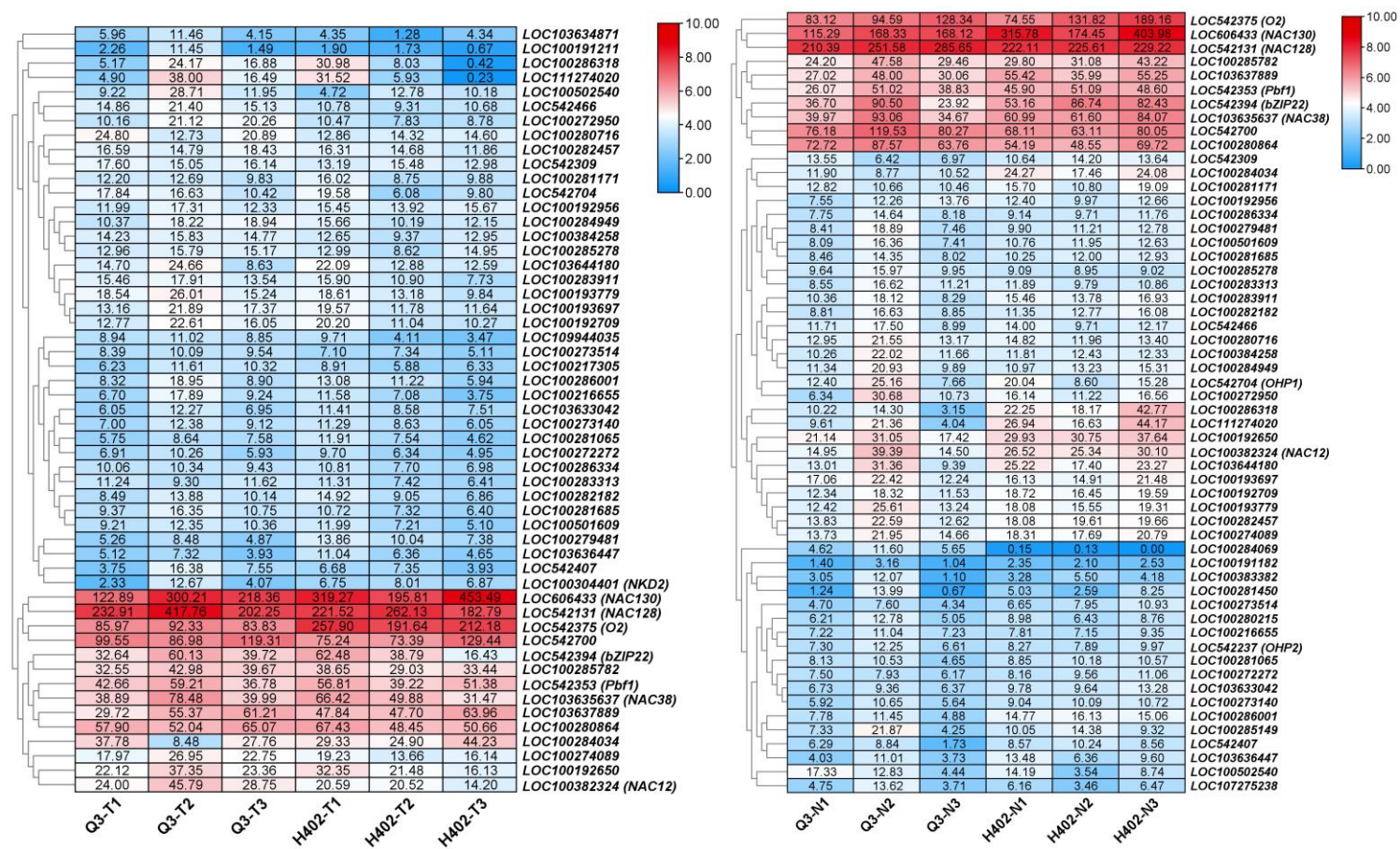

B

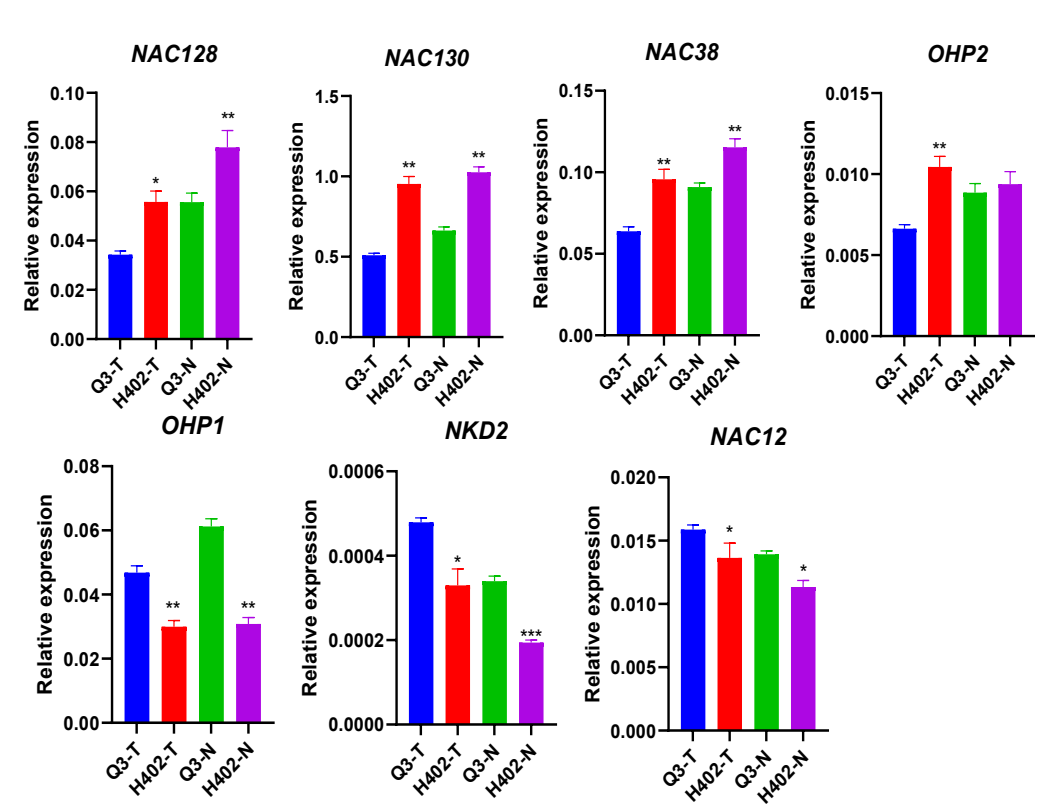

Figure S3

Supplement: Supplementary file 1 [file foods-14-03628-s001.zip › Supplementary Figure3.pdf]
